# Prediction of DNA binding motifs from 3D models of transcription factors; identifying TLX3 regulated genes

**DOI:** 10.1093/nar/gku1228

**Published:** 2014-11-26

**Authors:** Mario Pujato, Fabien Kieken, Amanda A. Skiles, Nikos Tapinos, Andras Fiser

**Affiliations:** 1 Department of Systems and Computational Biology, Albert Einstein College of Medicine, 1300 Morris Park Ave., Bronx, NY 10461, USA; 2 Department of Biochemistry, Albert Einstein College of Medicine, 1300 Morris Park Ave., Bronx, NY 10461, USA; 3 Macromolecular Therapeutics Development, Albert Einstein College of Medicine, 1300 Morris Park Ave., Bronx, NY 10461, USA; 4 Molecular Neuroscience Laboratory, Geisinger Clinic, 100 North Academy Avenue, Danville, PA 17822, USA

## Abstract

Proper cell functioning depends on the precise spatio-temporal expression of its genetic material. Gene expression is controlled to a great extent by sequence-specific transcription factors (TFs). Our current knowledge on where and how TFs bind and associate to regulate gene expression is incomplete. A structure-based computational algorithm (TF2DNA) is developed to identify binding specificities of TFs. The method constructs homology models of TFs bound to DNA and assesses the relative binding affinity for all possible DNA sequences using a knowledge-based potential, after optimization in a molecular mechanics force field. TF2DNA predictions were benchmarked against experimentally determined binding motifs. Success rates range from 45% to 81% and primarily depend on the sequence identity of aligned target sequences and template structures, TF2DNA was used to predict 1321 motifs for 1825 putative human TF proteins, facilitating the reconstruction of most of the human gene regulatory network. As an illustration, the predicted DNA binding site for the poorly characterized T-cell leukemia homeobox 3 (TLX3) TF was confirmed with gel shift assay experiments. TLX3 motif searches in human promoter regions identified a group of genes enriched in functions relating to hematopoiesis, tissue morphology, endocrine system and connective tissue development and function.

## INTRODUCTION

Gene regulation depends to a great extent on site-specific transcription factors (TFs) that recognize and bind specific DNA sequences in or near promoter regions of genes. TFs often act in concert to modulate the transcriptional activity of RNA polymerase II (1,2). Extensive knowledge of TF binding specificities provides insight into gene regulatory network architectures and functions (3), making it possible to study network level phenomena, such as mutational robustness (4) or subfunctionalization upon gene duplications (5).

Several high-throughput experimental techniques have been developed to determine TF binding specificity, such as protein binding microarrays, mechanically induced trapping of molecular interactions, high-throughput SELEX procedures and several more, which have been comprehensively reviewed by Stormo and Zhao (3). All these techniques are providing *in vitro* binding specificities that are not trivial to transfer for *in vivo* conditions, where a combined effect of additional interactions with co-factors, with enhancers, the accessibility of chromatin and the combinatorial nature of multiple TF binding sites can all influence binding (2). A limited collection of experimentally determined TF binding motifs are cataloged in databases, such as JASPAR (6), UniPROBE (7) and TRANSFAC (8).

Computational techniques have been developed to augment our knowledge about TF binding specificities and, currently, there are close to 200 sequence-based (9) and around 17 structure-based (10) algorithms in the literature. Sequence-based methods exploit statistical (11,12) or enumerative approaches to identify TF binding sites from ChIP-chip, ChIP-seq, promoter or genomic sequences (13–16). The prediction accuracies of nine of these sequence-based algorithms were compared on TF binding data sets from RegulonDB (17) using the Motif Tool for Assessment Platform, showing similar performances (9,18). Structure-based algorithms take advantage of known 3D structures of TF-DNA complexes. These algorithms have a variety of implementations, including the use of crystal structures and computational models obtained from homology modeling or computational docking techniques. Threading and various types of enumeration of bound DNA sequences can be used to explore possible binding complexes. Structure-based methods also differ in the level of structural optimization employed and in the type of scoring function used to evaluate binding affinity (19–24). Despite the fact that experimental structures cover only about 1% of a typical genome, accurate computational models could be built for about half of the genome (25). Nevertheless, the use of homology models for TF binding site prediction has only been anecdotally used (19–24) and has not been explored systematically.

Until now, the primary focus of structure-based methods was to recapitulate binding as observed in experimentally solved crystallographic structures (10). Additionally, all structure-based methods are described as protocols and no software packages are available to allow calculations for TFs of interest.

We developed the TF2DNA program for the prediction of TF binding preferences. TF2DNA is based on a novel structure-based computational method for the determination of TF regulatory sites. TF2DNA builds a homology model of a provided TF sequence using the most similar available template TF structure, from a manually curated structural collection of TF-DNA complexes. Starting from the homology model, the algorithm enumerates and constructs TF-DNA structural models for every possible DNA sequence. Possible steric clashes at TF-DNA interfaces are resolved and proper alignment of side chains and nucleotides is achieved by applying an energy minimization protocol in a molecular mechanics force field. Finally, an atomistic knowledge-based potential is used to obtain the relative binding affinities in every complex structure.

The accuracy of TF2DNA was benchmarked by comparing TF binding motif predictions to a set of 311 experimentally verified Position Weight Matrix (PWM) models of TF motifs, obtained from the JASPAR and UniPROBE databases. TF2DNA correctly predicts motifs in 81.4% of the cases where target-template sequence similarities are greater than 40%. Below 40% target-template sequence identity the success rate is 44.6%.

Subsequently, TF2DNA was used to predict 1321 binding preferences of 1825 putative human TF sequences, for which accurate homology models could be constructed. Given the benchmarked accuracy of the method, we estimate that about 945 of these motifs should be correct. As an anecdotal experimental validation of the approach, we functionally characterized the human T-cell leukemia homeobox 3 TF (TLX3). The predicted DNA binding motif for TLX3 was experimentally confirmed using gel shift assays. The TLX3 motif was searched within all human promoter sequences, identifying 2173 potentially regulated genes The 1000 best-ranking TLX3-regulated genes were used for functional enrichment analyses. In qualitative agreement with earlier studies (26,27) these genes fall into broad functional categories of hematopoiesis, tissue morphology, endocrine system and connective tissue development and function. We believe that the current method is broadly applicable for similar functional characterization of other TFs of interest or for the analysis of genome-wide TF regulation studies.

## MATERIALS AND METHODS

### Collection of curated TF-DNA structural templates

A manually curated collection of TF domains in complex with DNA was obtained from the available crystal structures in the Protein Data Bank (PDB) (28). The following protocol was used: (i) All PDB structures were collected that contain both protein and DNA molecules and had a crystallographic resolution better than 2.5 Å; (ii) Entries were manually filtered and proteins that are not sequence-specific TFs were removed; (iii) Redundancy was removed at 90% sequence identity using the cd-hit clustering program (29); (iv) Protein chains were separated into single files except in case of obligate TF homodimers. We kept only the part of DNA molecules that make contact with the protein, requiring a maximum distance cutoff of 4.5 Å between any two atoms of the protein and the DNA. We also kept crystal water molecules intact on the interface, since in many cases these mediate protein-DNA interactions; (v) Complexes with unnatural and missing bases were removed. Ultimately, the curation process resulted in 171 high quality TF-DNA complexes.

### Building homology models of TF-DNA complexes

For each TF sequence (target), we built homology models using our collection of curated TF-DNA complexes as templates. Target to template sequence alignments were obtained using hidden Markov models (HMM). HMMs were constructed using the package HHalign (30), with the following settings: 3 Psi-Blast iterations with *e*-value cutoff of 10^−4^ and profile construction using a minimum sequence identity with target sequences of 30%. HHalign also uses secondary structure information to produce a more sensitive local alignment. Each target TF sequence is aligned to all TF sequences in our curated structural collection. The resulting hits are filtered by requiring full coverage of the DNA binding site residues in the template and an HHalign probability score higher than 70%. Protein homology models were built with Modeller in complex with DNA (31). All DNA bases in the TF-DNA complex homology model were swapped in all possible combinations. In this way, 4*^k^* TF-DNA derived models are generated, where *k* is the length of the DNA motif in contact with the protein in the crystal structure (within 4.5 Å between of any protein-DNA atomic pair). For computational feasibility, we restricted the modeling exercise to templates where the DNA had at most nine base pairs contacting the protein. The original nucleic acid bases were stripped from the coordinate file and replaced by those corresponding to the desired sequence using the program psfgen from NAMD. The triad of one nitrogen and two carbons attached to the sugar ring were retained to maintain the directionality and planarity of the base. TF structures remained untouched. To optimize the interactions between interfacing atoms, the resulting TF-DNA complexes are minimized for 100 steps using the conjugate gradient algorithm in NAMD 2.6 with the CHARMM force field (32). The simulation took place in vacuum, retaining all interface water molecules resolved in the crystal structure.

### Obtaining binding site preferences

An all-atom, distance-dependent knowledge-based potential (33) was used to obtain scores (*S*_RV_) that estimate the free energy of binding of the modeled TF bound to each of the 4*^k^* possible k-mers. The potential function considers protein and nucleic acid heavy atoms in a residue-specific manner and maps the observed distances *d_ij_*, between atoms *i* and *j*, to a set of distance bins. The following energy function parameters were used: (i) a maximum cutoff distance of 10 Å, considered between any two interface atoms, (ii) a 3 Å distance for the first bin and (iii) 1 Å distance for the remaining 7 bins, giving a total of 8 distance bins. The potential was trained using the database of 171 TF-DNA crystal structures. Each complex was re-modeled with Modeller using its own PDB structure as template and their TF sequences as targets to generate a ‘self-modeled’ version of the database.

The knowledge-based potential scores, *S*_RV_, are normalized in the following way:

\begin{equation*} S_{{\rm norm}} = (S_{{\rm RV}} - S_{RV}^{{\rm lowest}} )/(S_{{\rm RV}{\rm }}^{{\rm highest}} - S_{{\rm RV}}^{{\rm lowest}{\rm }} ), \end{equation*}

where 0 represents the strongest protein-DNA association and 1 the weakest. The normalized scores are later transformed into relative binding affinities, *K_a_*, by considering the bimolecular binding reaction $(T + D\mathop \leftrightarrow \limits^{K_a } TD),$ where *T* represents the unbound TF, *D* the unbound DNA and *TD* the TF-DNA complex:

\begin{equation*} K_a = e^{ - \frac{A}{{K_B T}} \cdot S_{{\rm norm}} } \end{equation*}

where *A* is a proportionality constant in units of *K*cal/mol, *T* is the temperature in Kelvin and *K_B_* the Boltzmann constant in *K*cal/mol·*K*. *A* controls the slope of the exponential. This transformation gives near-zero binding affinities to the majority of the putative binding sites. We further applied a cutoff, $K_a^{{\rm cutoff}}$, which defines the set of specific TF binding sites. We explored these two parameters and found the best results with *A* = 4.74 *K*cal/mol at 298 K and $K_a^{{\rm cutoff}{\rm }}$ = 0.25. The number of TF binding sites was restricted to a maximum of 300 in cases, where $K_a^{{\rm cutoff}}$ produced a larger number.

### Building position weight matrices

Binding motifs were generated to model binding preferences for each TF. From the set of 4*^k^* DNA sequences tested for binding, only those with affinity values above a relative affinity cutoff of *γ* = 0.25 were selected to build a PWM. The sequences were aligned and their corresponding affinity values were used to weight the contribution of each base in the final PWM model.

### Benchmarking with RosettaDNA

We modified our algorithm and replaced the knowledge-based potential with the RosettaDNA energy function to estimate binding strengths. The algorithm produces models of the TF in question bound to every possible DNA sequence of length *k* and binding strengths are calculated with the RosettaDNA potential, which includes a protein-DNA interaction component that describes base-readout (direct-readout) mechanisms and deformation energies of the DNA sequence to include shape-readout mechanisms (indirect-readout) (23). Precise protocols used with the RosettaDNA program are provided in the Supplementary Material.

### Computing similarity between motifs

The similarity between PWMs of two motifs was assessed by computing *P*-values using the Fisher–Irwin exact test (34). One motif was allowed to slide over the other and the *P*-value was evaluated for each alignment. We also calculated sliding *P*-values for the forward and reverse complement versions of the sliding motif. Finally, the best alignment was determined by identifying the best *P*-value. *P*-values depend on the length of the motif alignment, therefore these cannot be used to compare motifs with different alignment lengths. Short motifs with lengths of 3–4 nucleotides will easily find good matches (better *P*-value) virtually within any other motif than longer ones (poorer *P*-value). To alleviate this problem, we transformed motif similarity *P*-values to motif alignment length-independent statistical *Z*-scores by comparing *P*-values against a non-redundant set of experimentally determined motifs that we used as decoys. A non-redundant set of decoy motifs was constructed using a conservative Fisher–Irwin *P*-value cutoff of less than 0.05 between any two experimentally determined motifs within the set. The final set contained 106 protein-DNA complex decoys (Supplementary Table S13). Motif *Z*-scores are computed by considering equal-length comparisons within the 106 decoy motifs. Therefore, high *Z*-scores would not only measure relative similarity but also the specificity or ‘uniqueness’ of the match.

### Clustering TF binding motifs

All-to-all similarity *Z*-scores (*Z*) were transformed to give a distance (*D*) matrix, using the following equation:

\begin{equation*} D = \frac{{abs(Z - 10)}}{{11.1}} \end{equation*}

The value 10 is the maximum *Z*-score we considered (most similar motifs) and 11.1 is the observed range of *Z*-scores. When *Z* equals 10, the equation assigns a distance of zero corresponding to the most similar motifs. The transformation was necessary to construct a phylogenetic tree using the program PHYLIP (35). PHYLIP generated a rooted tree using the hierarchical clustering UPGMA (Unweighted Pair Group Method with Arithmetic Mean) method. Clusters were chosen based on a similarity *Z*-score cutoff of 2.0, which means two motifs are similar with 95% confidence.

### Experimentally determined TF binding motifs

The union of two databases JASPAR (6) and UniPROBE (7) (854 entries in total) that contain experimentally determined binding sites was used to extract all the related TF protein sequences (1697 sequences). Redundancy was removed at 90% sequence identity using the program cd-hit (29), yielding 779 non-redundant TF sequences with known binding preferences. This step was necessary because JASPAR and UniPROBE do not always provide TF sequence information directly. In those cases where TFs linked to several sequences, only the first occurrence was considered. The final set of TF sequences, with known binding preferences, was matched against our manually curated structural collection using HMM to HMM alignments. This returned 311 matches in the 20–100% sequence identity range.

### Protein production

Codon-optimized synthetic cDNA of TLX-3 was purchased from GenScript. TLX-3 was cloned into the vector pMCSG7 containing a TEV cleavable N-terminal hexahistidine (His6) tag by ligation-independent cloning using a previously described protocol (36). The resulting plasmid was transformed into BL21(DE3)T1R (Sigma) containing the RIL plasmid (RIL) from Stratagene containing copies of genes that encodes tRNAs for rare codons. Transformed bacteria were grown in PASM 5052 (37) containing ampicillin and chloramphenicol (100 ug/ul and 34 ug/ul, respectively), for 5 h at 37°C. At that time the temperature was lowered to 22°C for overnight growth. The bacterial cells were pelleted and resuspended in Buffer A (20mM Hepes pH 7.6, 500mM NaCl, 20mM imidazole, 10% glycerol, 0.01% Tween-20, 0.1% NaN3, containing 1 mM PMSF and 4 u/ml of DNAse I). The cells were lysed using an EmulsiFlex C3 (Avestin) and separation of the lysate from the intact cells was achieved by centrifugation (16 500 *g*, 1 h). The protein was purified using an AktaExpress system. The clarified cell extract was passed through a His60 Ni Superflow 1 ml column (Clontech) at a flow rate of 0.75 ml/min. The column was washed with 10 column volumes of Buffer A. The protein was eluted with 5 ml of Elution Buffer B (20 mM Hepes pH 7.6, 500 mM NaCl, 500 mM imidazole, 10% glycerol). Then, the protein is loaded into a HiLoad 16/600 Superdex 200 (GE Healthcare Life Sciences) equilibrated with buffer C (20 mM Hepes pH 7.6, 150 mM NaCl, 5% glycerol and 5 mM DTT). The fractions containing the protein fractions were pooled together and concentrated using a 10K Amicon® Ultra Centrifugal Filters (EMD Millipore Corporation). The protein purity was determined by sodium dodecyl sulphate-polyacrylamide gel electrophoresis and the sequence confirmed by mass spectroscopy.

### Electrophoretic mobility shift assay (EMSA)

Complementary TF oligonucleotides (5′-NNNTTAATGTGTNNN-3′) and scrambled oligonucleotides (5′-NNNCGCTCAGACNNN-3′) were synthesized, labeled with biotin and annealed. Cold oligonucleotides (5′-NNNTTAATGTGTNNN-3′) were synthesized and annealed. In EMSA experiments, samples were prepared with 0.3 μg TLX3 recombinant protein, 1 μg Poly d(I-C), 5 μl of 4× Binding Buffer (10 mM HEPES pH 7.9, 100 mM KCl, 4 mM DTT, 0.5% Triton X-100 and 2.5% Glycerol), 0–12 μl of nuclease-free water and biotin-labeled TF probe (20 ng). A negative control sample was prepared without recombinant protein. In the cold assay, the sample was incubated at room temperature for 20 min with 5-fold excess of the cold TF probe prior to the addition of the biotin-labeled TF probe. In the scrambled assay, the biotin-labeled TF probe was replaced with 2 μl of the scrambled probe (200 μg). The binding reaction was performed at room temperature for 30 min. Protein-DNA complexes were then separated through gel electrophoresis on a 6% non-denaturing polyacrylamide gel using 0.5× tris-borate-EDTA buffer (TBE). Transfer was performed on a Pall Biodyne B nylon membrane using an electroblotting device with 0.5× TBE then fixed using a ultraviolet crosslinker. The membrane was washed, blocked, incubated with streptavidin-alkaline phosphatase and then developed using CDP-Star according to the protocol for non-isotopic detection of biotinylated DNA probes (Ambion Bright Star Biodetect Kit). The membrane was then exposed to autoradiography film for 5 min and developed.

### Scanning human promoter regions with binding motifs

All promoter sequences from protein-coding human genes, as present in the RefSeq database (38), were collected. Promoter regions were defined as: 1500 bp upstream and 500 bp downstream of transcription start sites (TSSs). The resulting 23 340 promoter regions were scanned using the Motif Alignment and Search Tool (MAST) (39). MAST takes a binding motif in the form of a PWM and searches the given DNA sequences. It calculates match scores for each binding site found by summing up the individual PWM frequencies of the matching letters within promoter sequences. Finally, it reports the set of binding sites with *P*-values calculated in comparison to a set of background sequence decoys. Binding sites were identified using a *P*-value cutoff of 0.0001.

## RESULTS

### Obtaining TF-DNA complexes and their binding motifs

The TF2DNA algorithm utilizes a 3D model of a TF to predict its binding preferences. 3D models of TF proteins could be obtained either from experimental sources or homology modeling (40). For homology modeling we built a structural database of templates by collecting all available crystal structures of TF-DNA complexes from the PDB (28). These structures were manually curated to include only the binding domain of each TF. The resulting 171 TF-DNA complexes were used as templates to build homology models (Supplementary Table S1).

**Figure 1.**
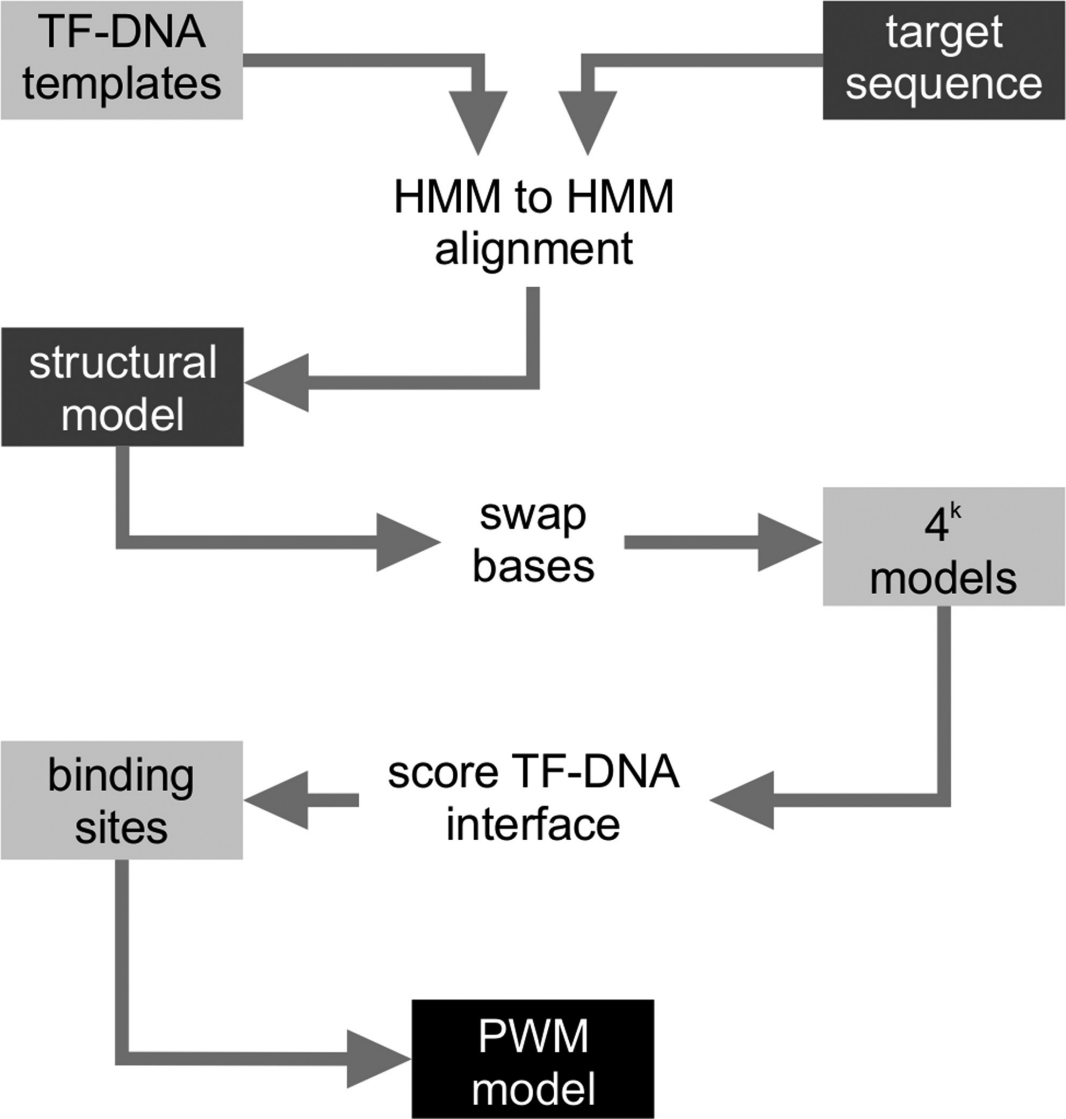
Flowchart of the TF2DNA method. For each TF target sequence a profile HMM is built. The HMM of the target is aligned to all pre-calculated template HMMs in our collection of manually curated TF-DNA structures. The best alignment is identified using 100% coverage of the template binding site and highest sequence identity of the aligned region. The obtained alignment is then used to generate a homology model of the target sequence. Alternative TF-DNA complex models are obtained by swapping the DNA bases by all possible sequences of length *k* (length of the DNA in the model structure). Using a knowledge-based atomistic pair potential we score all the resulting 4*^k^* number of TF-DNA interfaces. The scores are normalized in the range 0–1 and a cutoff is applied to identify the group of sequences that define the target TF binding sites. The resulting binding sites are used to model the binding motif as a position weight matrix.

The method starts by aligning the target TF protein sequence to all known TF sequences in the template database using HMM profile alignments, supplemented with predicted secondary structure information (Figure 1). The most suitable template is selected by considering the extent of coverage of the binding site residues and the sequence identity of the aligned region. A comparative protein model of the target sequence in complex with DNA is generated with Modeller (31) using the target-template HMM-HMM profile alignment. The program Modeller was suitable for this task because of its ability to model protein-DNA complexes, which functionality is not provided in most homology modeling programs. Next, the complete library of bound DNA fragments is explored within the complex by building additional TF-DNA complex models where the DNA bases are replaced by all possible 4*^k^* sequences (*k* is the length of the DNA in contact with the protein). Possible atomic clashes at the TF-DNA interface are relaxed using a minimization protocol in a molecular mechanics force field. The resulting models are ranked based on their TF-DNA relative affinities, which are calculated using a knowledge-based potential (33) that was trained on structures of TF-DNA complexes. The set of best scoring DNA segments are used to construct position weight matrices, also referred to as binding motifs, which model the binding preferences of the TF.

The TF2DNA program generates ∼22 mutants per minute, considering a system with ∼1000 atoms (e.g. PDB code: 1AAY – Early Growth Factor 1 TF). Therefore, a TF binding motif prediction can be completed in ∼2 days time in case of an 8 base pairs long DNA sequence, using a single core on an Intel® Core™ i7–870, 2.93GHz. The calculation is easily parallelizable, therefore the same job takes ∼6 h using an 8-core computer and it takes about a half hour if a computing cluster with 10 such nodes is available. A computer program fully implementing the algorithm is available for download at http://www.fiserlab.org/our_programs.htm.

### Benchmarking the method with experimentally determined binding motifs

The performance of the method was benchmarked using a testing set of 311 TF non-redundant sequences with experimentally determined TF binding motifs as found in the JASPAR (6) and UniPROBE (7) databases.

Several training schemes for the knowledge-based potential were considered depending on some aspects of the shape readout (involving static features of the structure only) that characterized the DNA and the secondary structures of the TF involved in the interaction. For this purpose we divided the set of 311 sequences according to six TF binding modes: helix binding the major groove of the DNA, helix/loop combination binding the major groove, strand/loop combination binding the major groove, strands binding the minor groove, strands binding the major groove and helix binding the minor groove (Supplementary Table S2 and Figure S1). A special training set of crystal structures modeled onto themselves with Modeller (‘self-models’) was utilized. Supplementary Figure S1 shows the overall performances for each training scheme. Performances were comparable for the case of training the potential with all the 171 crystal and self-modeled structures in our database, and showed that the potential is not sensitive to small diversifications of the training structures. Training with subsets of TF structures that recognize the major groove of the DNA with helices also resulted in a similar performance. This is probably a consequence of the fact that the training set, as well as the testing set of 311 TF sequences, are overwhelmingly composed of TFs binding the major groove of the DNA molecule with helices (Supplementary Table S2). The effect of relaxing the structures after mutations provides a relatively modest but significant (3–5% improvement). A breakdown of results (Supplementary Figure S1) shows that the increase in performance is localized to the most difficult modeling cases (∼9% increase), indicating that structural relaxation is particularly beneficial in cases where the target-template identity is less than 50%.

Inherent uncertainties in the experimental determination of TF binding motifs result in differently recorded preferences; for example, the MATα2 TF binds the consensus sequences AATTACATG and AACAATAG, respectively, as documented in the JASPAR and UniPROBE databases. To avoid these ambiguities in the benchmark, we set up a control set of 11 TF sequences for which we found an available TF-DNA complex crystal structure with at least 90% protein sequence identity, and where the experimentally determined binding motifs were essentially identical in both JASPAR and UniPROBE databases. Predicted and experimentally determined motifs were compared using a similarity *Z*-score measurement (see Materials and Methods). A prediction is assumed to be correct above 95% confidence level (*Z*-score ≥ 2) when compared to the experimental data. The similarity *Z*-scores of the control set (11 cases) span a range from 1.9 to 10, with 10 cases above 2 (Supplementary Table S3). In other words, the method returns correct predictions when the TF-DNA complex structure is known or a highly accurate structural model can be built (with more than 90% target-template identity). Some motifs, such as Gabpa, ETS1 and Mafb are only 3 base pairs long, but sufficiently unique among the set of 106 decoy motifs to return a significant *Z*-score.

**Figure 2.**
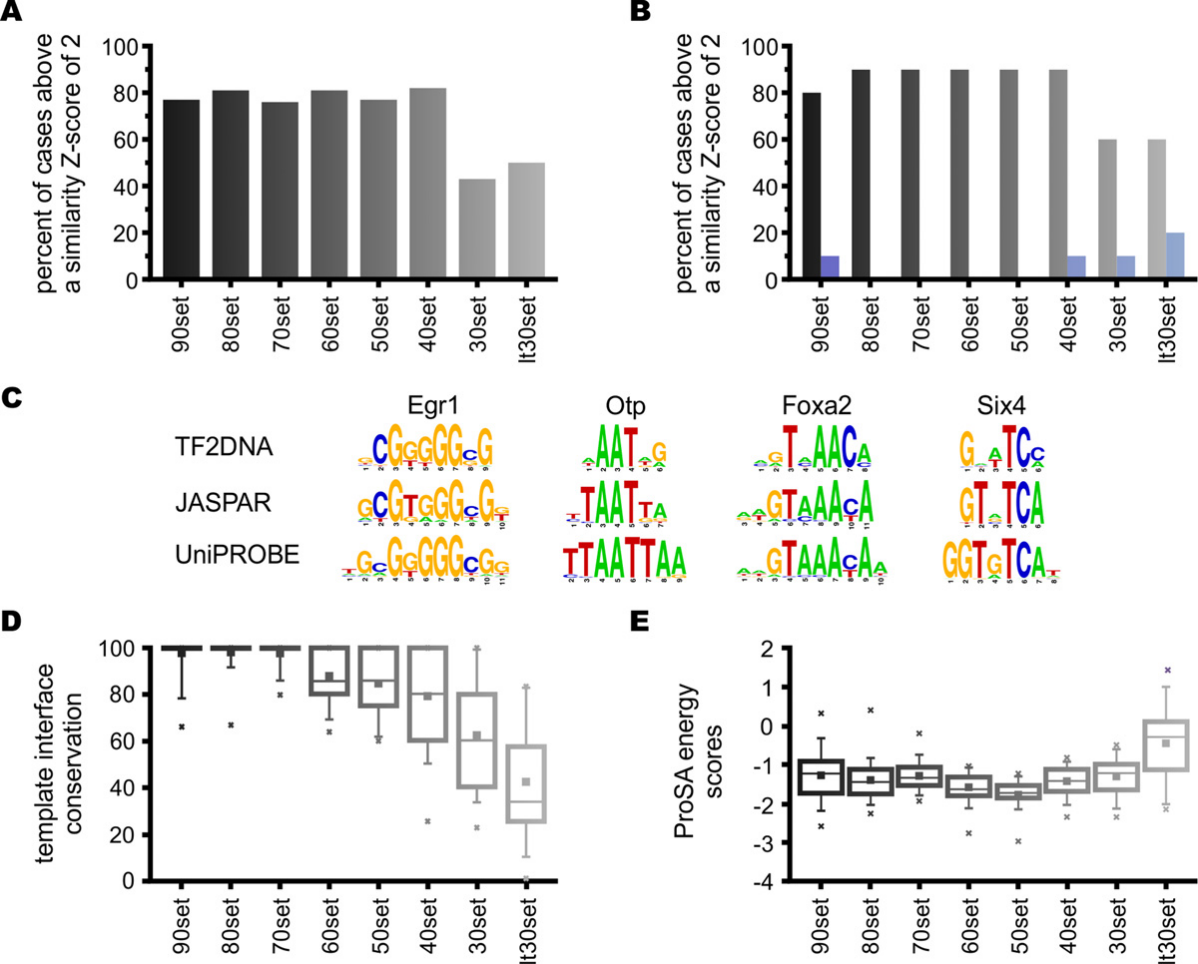
Performance of TF2DNA at predicting TF binding motifs. (A) Performance of the TF2DNA method as measured by the percent of correctly predicted test cases. A prediction is correct when the predicted and experimentally determined sequence motifs (from JASPAR and/or UniPROBE) show a similarity *Z*-score of 2, or higher. A *Z*-score of 2 or higher means that two compared motifs are similar at 95% confidence level. Performance is shown for eight test-sets of TF, which sets differ by their target-template sequence identity. (B) Comparison of performances between the Robertson–Varani knowledge-based potential (gray bars) and the RosettaDNA potential (blue bars), similarly to panel A. Each test-set bin contains 10 randomly chosen cases. Here, the plot shows the percent of test cases above a motif similarity *Z*-score of 1 (i.e. correct prediction is already assumed at 66% confidence level.). This lower expected confidence level was chosen to enhance the signal produced by RosettaDNA, which did not predict any motif correctly when *Z*-score expectation was set at 2. (C) Four examples of predicted motifs at different TF target-template sequence identities are shown with sequence logo representations: Egr1 (early growth response protein 1), Otp (orthopedia homolog from *D. melanogaster*), Foxa2 (forkhead box A2 protein) and Six4 (sine oculis-related homeobox 4 homolog from *D. melanogaster*). The sequence identities to their templates (and their database motif similarity *Z*-scores) are: 100% (10), 69% (4.2), 49% (10) and 31% (2.1), respectively. (D) Boxplots showing the distributions of template interface conservations (TIC), which is measured as the percent target-template sequence identity of residues in direct contact with DNA bases (within 4.5 Ang of any base atom in the template structure). (E) Boxplot of distributions of residue contact energies in the modeled structures as estimated by ProSA energy scores (71). Boxplot interpretation: filled squares show averages, boxes display quartiles, whiskers are at 5% and 95% of data and crosses show minimum and maximum values.

The sequence identities of the 311 protein sequences in the test set range from 20% to 100% to a known TF sequence in the curated collection of TF-DNA complexes. The set is divided into eight increasingly more challenging subsets (in terms of modeling difficulty), binned into 10% sequence identity intervals, except bins with less than 30% target-template sequence identity, which were combined due to insufficient number of cases. TF models built on target-template alignments with ≥40% sequence identities achieved an average success rate of 78.4%, using similarity *Z*-score cutoff of 2, to define successful predictions, which refers to 95% confidence level (Figure 2A). The performance remains fairly even across the bins in the range 40–100% (>70%) (Figure 2A and Supplementary Table S4).

**Figure 3.**
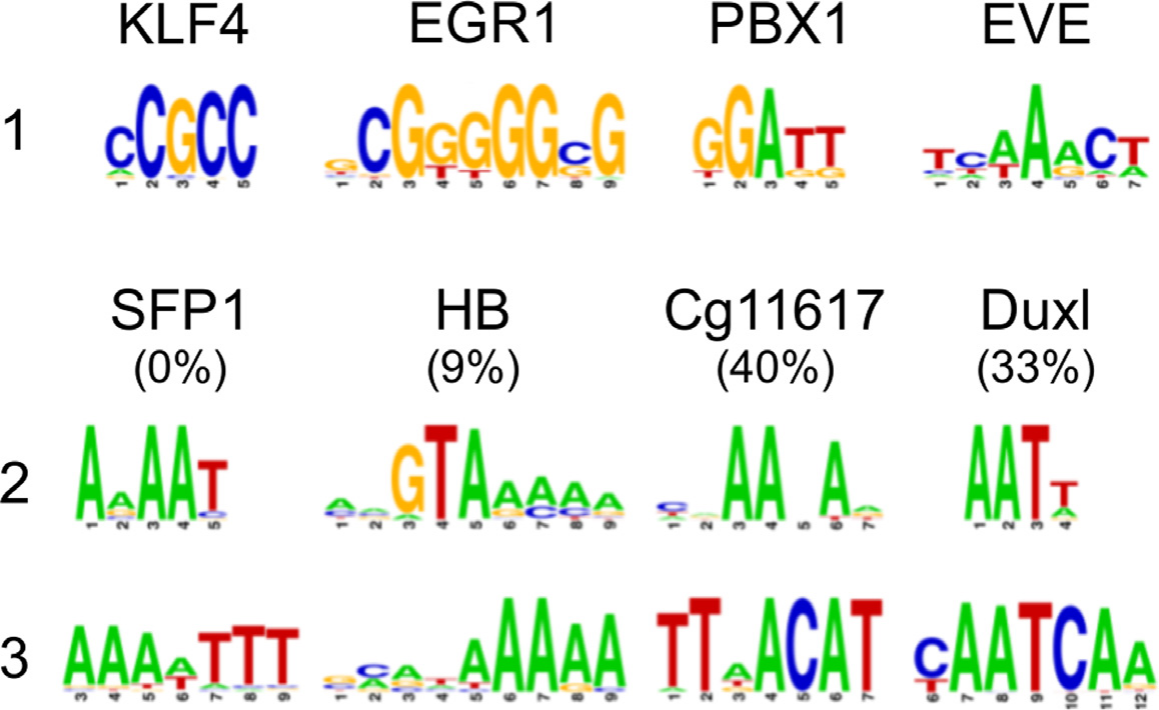
TF2DNA predictions are template independent. Examples of template sequence independence of the motif predictions. On the first row, motifs produced by the TF protein sequences of the templates: Klf4 (gut Kruppel-like factor 4), Egr1 (early growth response protein 1), PBX1 (pre-B-cell leukemia homeobox) and eve (even-skipped). The second row shows the predicted motifs for the TF target sequences: SFP1 (Split finger protein 1), hb (hunchback protein), CG11617 (unknown protein with homology predicted TF function) and Duxl (double homeobox B-like protein). Each target sequence was modeled using the corresponding same-column template structure. Their respective TIC values are indicated within parenthesis. The third row shows their experimentally determined motif according to the JASPAR or UniPROBE databases. Expected motifs are correctly predicted (with *Z*-scores above 2) even in such cases, as SFP1, where the residues at the protein-DNA interface were completely replaced.

Figure 2C shows four examples of predicted motifs that are similar to their experimental counterparts (*Z*-scores ≥ 2). These example predictions cover all the spectrum of target-template TF sequence identities, going as low as 31% in the case of the Six4 TF. The experimental motifs are sometimes longer than the ones predicted by TF2DNA. This could be due to the fact that while we start from TF-DNA structural complexes and consider only those DNA positions that make physical contacts with the TF, the high-throughput experiments capture all positions with some preference and may add extra nucleotides at each end. The experimentally determined motif of EGR1 have 10 and 11 base pairs in JASPAR and UNIPROBE, respectively, despite the fact that only 9 nucleotides make physical contact with the protein (Figure 2C). In another example, DNA binding domains of TFs Otp and Six4 contact a maximum of 6 bases and Foxa2 contacts 8 bases in the crystal structures, while the experimentally derived motifs add extra bases. Another reason of differences in length of motif definitions is due to the incorrectly captured preferences at certain positions in the DNA, which situation is more often encountered when the target-template protein sequence alignment is incorrect, due to low sequence similarities. The latter reason would explain the loss of information in Figure 3's TF2DNA predictions.

The success rates quickly decline when the target-template sequence identity drops below 40% (Figure 2A). This decay in performance coincides with a deteriorating conservation of template protein residues in the modeled binding site environment (expressed as the target-template sequence identity of the residues at the protein-DNA interface; defined as residues within 4.5 Å of any DNA atom) (Figure 2D and Supplementary Table S4). The ‘conservation of template interface’ (CoTI) remains high, with an average of 80%, even as low as 40% global target-template sequence identities. The CoTI decreases from ∼80% to 62%, when the global sequence identity drops below 40%. Another factor influencing the performance of the method is the quality of the homology models. The thermodynamic stability of the structures, determined by their ProSA energy scores (41), is used as an estimate for the quality of the models (Figure 2E). ProSA energy scores show that reasonable models were constructed for almost all the test sets. The exception is the lt30set (less than 30% global sequence identity), which shows an increased fraction of low quality models (higher ProSA energy scores). The prediction accuracy seems to level off above 40% global sequence identity, to about 80% success rate. This leveled performance could be attributed to the limited choice of template structures, causing frequent reuse of templates. Out of the available structures, only 43% were used as templates in the control set, from which 67% were reused. The lowest template reuse rate is seen in the 90set, because the target-template sequence similarity requirement is more stringent.

In general, the method shows good performance even when target-template protein sequence identities are as low as 40%. The method seems to be limited by template availability. The CoTI measure appears as a good predictor of success, followed by model quality, as estimated by ProSA protein energy scores.

### Benchmarking against RosettaDNA

Currently, there are no alternative methods that provide a software package for the prediction of TF binding motifs, however, our algorithm can accommodate other potentials for the evaluation of TF-DNA binding interaction strengths. Therefore, for benchmarking purposes, we replaced the Robertson–Varani knowledge-based potential in our program with the RosettaDNA (23) potential (see Materials and Methods). The RosettaDNA potential takes on average five times longer than TF2DNA to optimize the protein-DNA interface and compute binding strengths, therefore we predicted binding motifs for 80 representative TFs (out of the total 311 in our test set) (Figure 2B). The 80 representative cases were compiled by randomly choosing 10 cases from each of the eight test set bins. According to the results (see Supplementary Material), RosettaDNA was not able capture the specificity of any studied TF in a statistically significant manner (Supplementary Figure S2 and Table S5). A possible explanation of this is that RosettaDNA has been optimized to reproduce protein-DNA affinities at the expense of losing specificity (23,42), whereas the Robertson–Varani knowledge-based potential was developed to distinguish binding sites among large sets of decoy DNA sequences.

### TF2DNA predicts motifs in a template-independent manner

Due to the relatively small size of the curated structural collection of TF-DNA complexes, it is common that the same templates are used to model different TF target sequences, which may have different binding specificities. This provides an opportunity to explore whether TF2DNA is able to correctly predict binding motifs for target protein sequences modeled on templates whose sequences recognize different motifs. Figure 3 shows four examples: SFP1 (Split finger protein 1), hb (hunchback protein), CG11617 (unknown protein with homology predicted TF function) and Duxl (double homeobox B-like protein). Despite the very low CoTI (0%, 9%, 33% and 40%), these example TFs produced correct binding motif predictions. In the case of SFP1, not a single template residue was conserved at the protein-DNA interface in the model. Nevertheless, the TF2DNA correctly predicted the experimental motif: AAAAT. Meanwhile, the protein sequence of the template structure (KLF4) that was used to model SFP1, recognizes an entirely different sequence: CCGCC. The results suggest that TF2DNA is able to correctly capture binding preferences, even if all the TF residues located at the binding site are built from scratch.

### Prediction of human TF binding motifs

About 6–7% of all eukaryotic genes are estimated to be DNA binding proteins (41). Previously, a census of human sequences found 1987 genes (1825 after removing sequence redundancy at 90% identity) to be sequence-specific DNA-binding TFs (43), but only a small percentage of these TFs have known binding motifs. Up to 380 experimentally obtained human TF binding motifs have been collected by Jolma *et al.* (44). Kulakovskiy *et al.* gathered ∼400 TF binding motifs by adding mined data (45). Table 1 shows the availability of TF binding motifs in different organisms (43–50,51). The best studied organism is *Saccharomyces cerevisiae* or budding yeast, with 83% coverage of known experimentally determined TF binding motifs, although this organism has only a total of 203 TFs to cover. The number of expected TFs in humans is eight times greater than in yeast, between ∼1500 and 1800. The coverage of TF binding motifs in humans is about 25% (39% considering unique TFs from combining all human datasets; 586 in total), leaving much room to achieve full coverage.

**Table 1.** TF binding motif and general statistics for different organisms

Organism name	Genome Size [Mb]	# proteins	# TFs	# binding motifs
*Homo sapiens*	3209.29	19 226	1825^a^	380 (25%)^g^
				268 (18%)^h^
			1500^b^	127 (8%)^i^
				4 (<1%)^j^
*Mus musculus*	2798.79	20 616	1266^b^	295 (28%)^j^
				76 (7%)^i^
*Drosophila melanogaster*	139.49	13 929	1052^c^	131 (13%)^i^
*Caenorhabditis elegans*	100.29	20 362	934^d^	23 (3%)^j^
				15 (2%)^i^
*Saccharomyces cerevisiae*	12.16	5906	203^e^	177 (87%)^i^
				92 (45%)^j^
*Escherichia coli*	4.64	4141	314^f^	202 (64%)^k^

^a^(Vaquerizas *et al.*, 2009) (43).

^b^(Fulton *et al.*, 2009) (47).

^c^(Pfreundt *et al.*, 2010) (48).

^d^(Reece-Hoyes *et al.*, 2005) (49).

^e^(Harbison *et al.*, 2004) (50).

^f^(Pérez-Rueda and Collado-Vides, 2000) (51).

^g^(Jolma *et al.*, 2013) (44).

^h^HOCOMOCO database (Kulakovskiy *et al.*, 2013) (45).

^i^JASPAR database (Bryne *et al.*, 2008) (6).

^j^UniPROBE database (Newburger and Bulyk, 2009) (7).

^k^RegulonDB (Gama-Castro *et al.*, 2011) (17).

General statistics for the most widely used model organisms and human. The number of proteins (column 3) was taken from the RefSeq database (38) and consists of the unique counts of all gene names (HUGO names (46)) annotated as ‘protein-coding’ (RefSeq transcript identifiers starting with ‘NM’). Column 4 contains the number of TFs as estimated by their corresponding sources. Column 5 shows the number of TF binding motifs that are available on different databases. The percent coverage based on the smallest number of TFs reported in column 4 is displayed within parenthesis.

TF2DNA predicted human TF motifs whenever a reliable homology model could be built. A reliable model must minimally cover all the binding site residues in the template structure. Additionally, the target-template protein HMM alignment probability should be higher than 70%. This resulted in 1321 human TF models, which means that currently ∼72% of all considered sequences can be reliably modeled. The models were built using 106 different template structures (out of a total of 171 in our curated structural collection); where 16 were used only once and 1 was used 501 (53%) times (Supplementary Table S6).

The predicted human TF binding motifs were grouped in bins of 10% width according to the percent sequence identity of the target-template aligned regions (Table 2). About 87% of all modeled human TFs were built on a template with more than 30% sequence similarity. If we consider the observed TF2DNA success rates from the benchmarking (fourth column in Supplementary Table S4), an estimate of 923 motifs would be accurately predicted, which accounts for a total of 71.5% of all 1321 modeled human TF sequences. In summary, our TF2DNA predictions approximately double the current knowledge on human TF binding motifs, from 586 to 923 motifs according to the data collected on Table 1.

**Table 2.** Models of human TFs

%ID range	Reliable models	Reliable models (cumulative)	TF2DNA performance	Correct targets	Correct targets (cumulative)
≥90	90	90	74.8	67	67
80–90	54	144	77.8	42	109
70–80	60	204	81.2	49	158
60–70	246	450	78	192	350
50–60	312	762	76.8	240	590
40–50	200	962	81.8	164	753
30–40	188	1150	49	92	845
<30	171	1248	45.2	78	923

The total number of targeted TF sequences is 1825 (obtained from list of Ensembl identifiers provided by Vaquerizas *et al.* (43)). First column shows the percent sequence identity between human TF target and their structural template. The fourth column shows the fraction of successful predictions by TF2DNA (at 95% confidence level) (from Supplementary Table S4). The fifth column shows the estimated numbers of correctly predicted targets, which is obtained by multiplying the number of reliably modeled targets (second column) and the calculated TF2DNA performance (fourth column).

### Human TF regulatory network

Each of the 1321 predicted TF binding motifs were used to search human promoter sequences and identify lists of putative protein-coding gene targets (regulated genes), in order to reconstruct the human TF regulatory network. The search was conducted on all available human promoter DNA regions, defined as 1500 bp upstream and 500 bp downstream of protein-coding gene TSSs. All human promoters were downloaded from the RefSeq database (38), reaching a total of 23 340 promoters that correspond to 18 515 unique genes. Analysis of promoter multiplicity (the number of promoters per gene) shows that 81.9% of genes have 1 promoter and 98.3% have less than 4 promoters (Supplementary Table S7). All the data was stored and organized in an SQL database called ‘TF2DNA database’ (accessible at http://www.fiserlab.org/tf/) which can be queried for general statistics, such as the distribution of the number of TFs that regulate each promoter (Supplementary Figure S3). According to TF2DNA predictions, a promoter is normally regulated by a median of 215 putative TFs. In addition, a TF can generally regulate a median of 3026 genes (Supplementary Figure S4). Similar amounts of regulated genes are observed in ChIP-chip or ChIP-seq experiments; for example: Satoh and Tabunoki (52) found 1441 target genes for the signal transducer and activator of transcription 1 (STAT1) protein, Satoh *et al.* (53) identified 2470 regulated genes for the nuclear respiratory factor 1 (NRF1) and Cheng *et al.* (54) found 3689 targets for the mouse signal transducer and activator of transcription 4 (STAT4). These high numbers seem odd, but promoter searches with short DNA binding motifs may return many false positive results. Similarly, ChIP experiments suffer from artifacts, where binding is detected at non-specific regions termed ‘hyper-ChIPable’, as observed by Teytelman *et al.* (55). It has been also extensively discussed in the literature that only a fraction of *in vitro* binding motifs (determined in *in vitro* experiments or predicted computationally) are true *in vivo* binding sites and only a fraction of these are functional (2).

### Clustering human TFs by binding motif similarity

Predicted human TF binding motifs were clustered according to their similarity (see Materials and Methods). The clustering generated 294 clusters (Supplementary Table S8), with the largest cluster containing 66 motifs. Qualitative inspection of TF names on each cluster serves as additional validation of the motifs. The largest cluster (cluster #1) is formed by homeobox (HOX) genes and other related TFs. For example, the TF BARHL1 (BarH-like homeobox 1) and its known homolog DLX2 (distal-less homeobox 2) also cluster together in this group. SOX (sex determining region Y-box) TFs also cluster together along with the SRY (sex determining region Y) protein (cluster #11). Distributions of cluster sizes (Supplementary Figure S5) show that most clusters have few members and most TFs are grouped together in few clusters.

### Experimental validation of TLX3 binding sites with EMSAs

**Figure 4.**
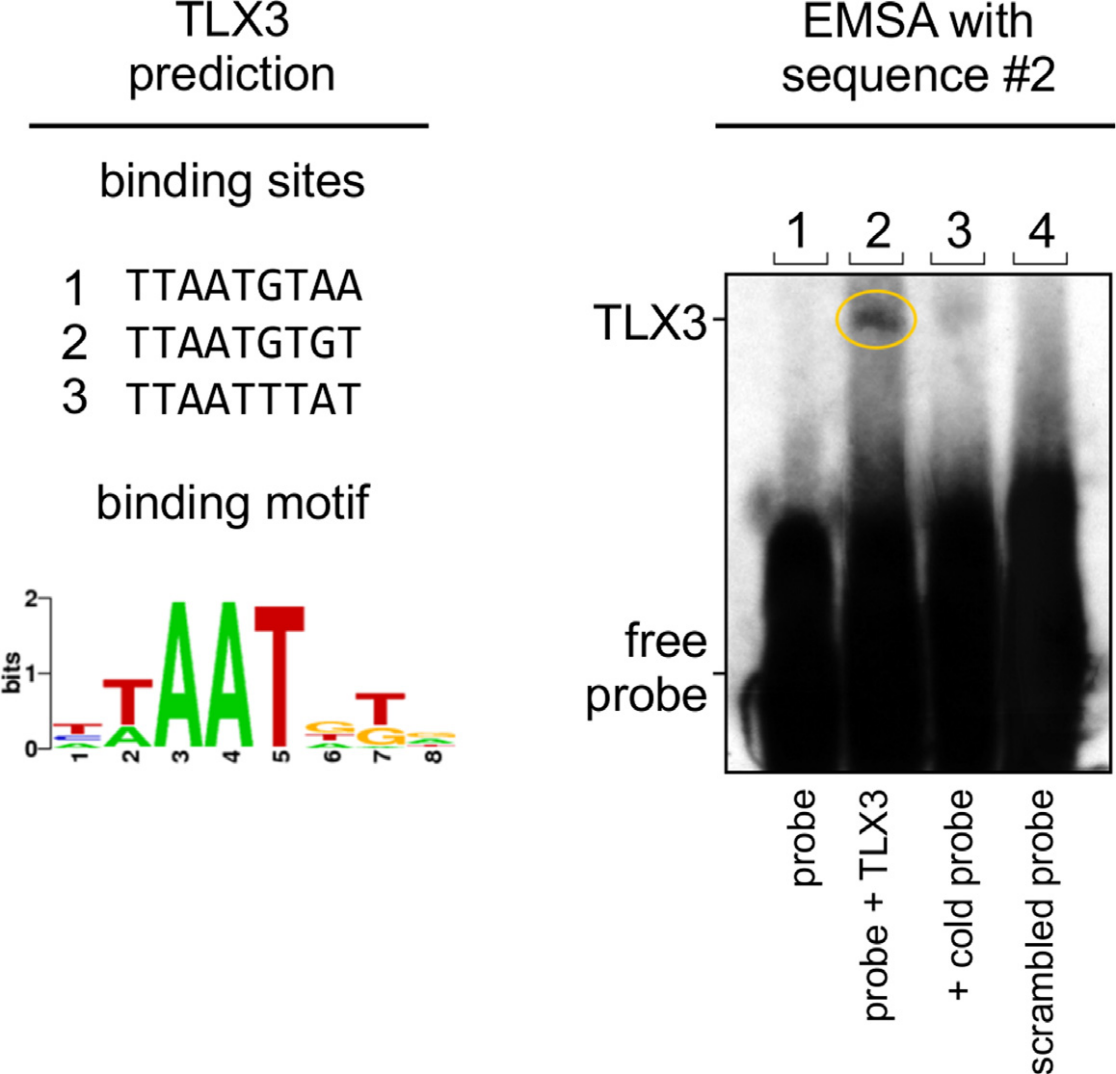
Experimental validation of predicted human TLX3 binding sequences. Left panel: Prediction of binding preferences for the human TLX3 TF. The top three predicted binding sequences, which were used for EMSA assays, are displayed as well as the consensus binding motif. The sequence highlighted in blue showed binding to TLX3 in the EMSA assay. Right panel: Results of the EMSA assay using sequence #2 (predicted sequence highlighted in blue), referred to as probe. Lane 1: Negative control (probe only). Lane 2: Biotinylated probe. Lane 3: Cold probe (competition assay). Lane 4: Scrambled probe. The yellow circle marks the shifted, bound motif with TLX3.

To experimentally test the prediction accuracies, a group of five proteins were selected from the set of human TF proteins described in the previous section: ARNTL2 (aryl hydrocarbon receptor nuclear translocator-like 2), LBX1 (ladybird homeobox 1), MSGN1 (mesogenin 1), NOTO (notochord homeobox) and TLX3 (T-cell leukemia homeobox 3). These proteins were chosen because they have some limited experimental functional annotation but their binding preferences are unknown meanwhile their target-template similarities were below 60%, where TF2DNA predictions are more challenging. Two proteins, ARNTL2 and MSGN1 failed in the protein expression and purification process. The remaining three successfully purified TFs were tested for their binding to the top three predicted DNA sequence motifs using EMSA. In one out of the three cases, TLX3, the EMSA experiment produced a gel shift due to binding of TLX3 to the second predicted motif (TTAATGTGT) (Figure 4). This shift was confirmed against a negative control, a scrambled probe and a competition assay. In case of the other two TF a failed EMSA is not necessarily an indication of a mispredicted binding motif. The protein binding domains may not be folded correctly or completely or the DNA binding domain is not sufficient to provide efficient binding, perhaps the TF may bind as a (hetero or homo) dimer. It is also important to note that the three TLX3 putative binding sequences were picked from 262 144 (4^9^) sequences, meaning that in this case the TF2DNA program was able to produce close to 100 000-fold enrichments of binding sequences. Also, in line with the *in silico* predicted success rates (Figure 2), about 40% of these test cases were expected to be correct.

### Genetic targets of TLX3

**Figure 5.**
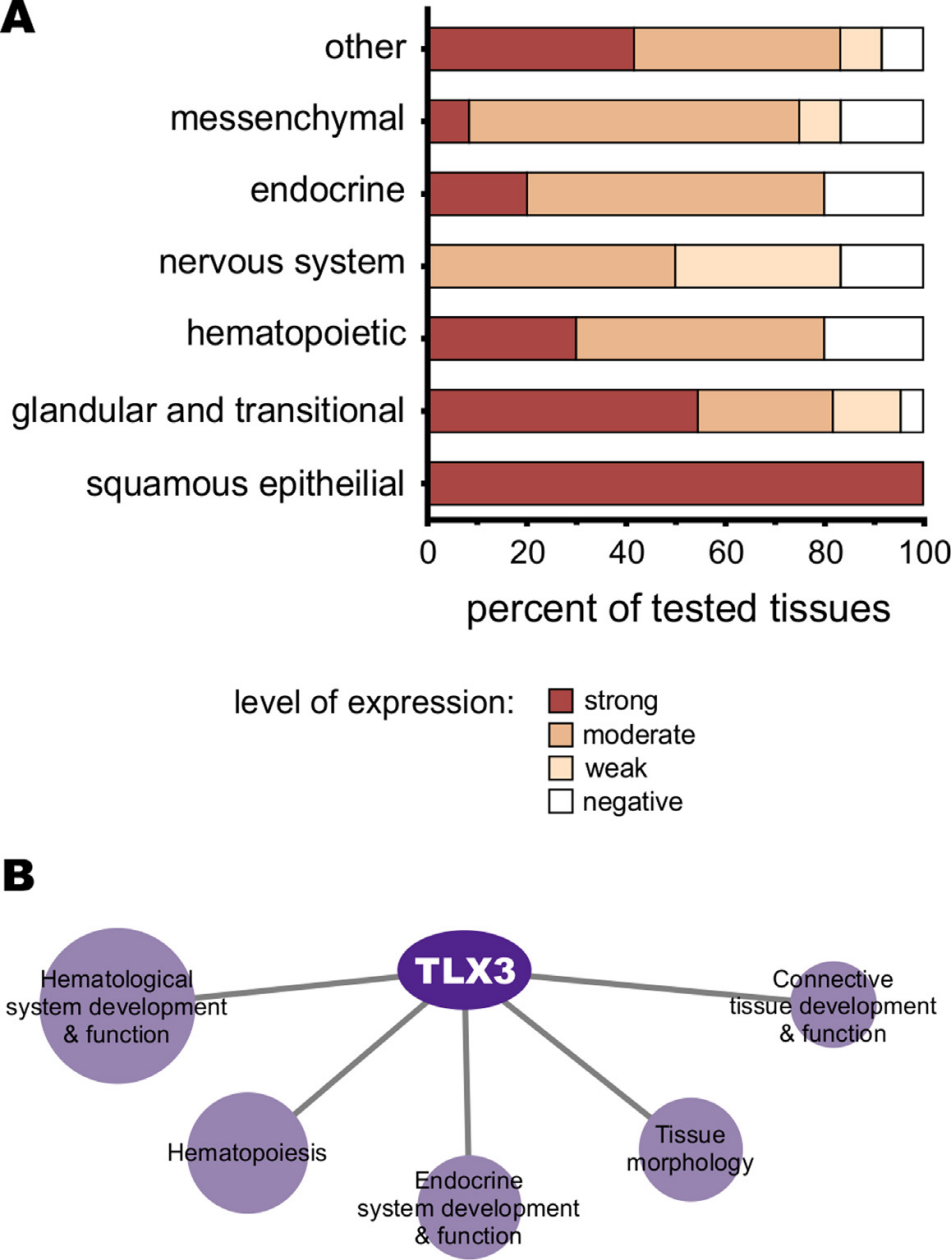
Predicted function of the TLX3 TF. (A) Protein expression levels of TLX3 as reported in the Human Protein Atlas (60). The tissue types were broadly grouped and the percent of observed expression levels were calculated for the tested subtissues within each category. Detailed expression levels in subtissues are presented in Supplementary Table S9. (B) Ingenuity pathway analysis of observed targets genes of TLX3 obtained with the TF2DNA predicted binding motif. The figure shows the five most significantly enriched networks in the physiological system development and function category. Sphere sizes are proportional (logarithmic scale) to the amount of genes populating the category. The TLX3 target genes that were enriched within this category are listed in Supplementary Table S12.

T-cell leukemia homeobox 3 (TLX3; also Hox11L2 or Rnx) is a sequence-specific TF with known functions in nervous system cells. Kondo *et al.* (26) found that overexpression of TLX3 in mesenchymal stem cells induced sensory and glutamatergic neuron markers. In another study, Lopes *et al.* (27) observed that overexpression of TLX3, in conjunction with RUNX1, induced ectopic expression of sensory channels and receptors in dorsal root ganglion cells in mice. Huang *et al.* showed that TLX3 was required for the expression of both proteins vasoactive intestinal polypeptide (VIP) and somatostatin (SST) in cholinergic neurons during late mice development (56). TLX3 has also been implicated in disease states in humans, such as in T-cell acute lymphoblastic leukemia or T-ALL. A common translocation of the TLX3 coding region places it under the control of an active promoter in T-ALL (57), interfering with critical stages of T cell differentiation (58). A systems biology analysis (59) of the regulatory circuit controlled by TLX1 and TLX3 in T-ALL found these factors as master regulators and identified the runt-related TF 1 (RUNX1) as a tumor suppressor. In addition, TLX3 is expressed in a diverse variety of tissue types but, despite the functional evidence discussed above, the normal function of TLX3 in cells other than nervous system cells remains unidentified. According to The Human Protein Atlas (60), TLX3 protein expression is observed in epithelial cells (squamous, glandular and transitional), hematopoietic, endocrine, messenchimal and other types of cells (Figure 5A and Supplementary Table S9).

Our reconstructed human regulatory network identifies 2173 TLX3 putative target genes (Supplementary Table S10). We used the best ranking 1000 genes (ranked by binding site scores) to perform a functional enrichment analysis using the IPA® software package (Ingenuity® Systems, www.ingenuity.com). The most enriched functions in the ‘physiological system development and function’ category include (1) hematological system development and function, (ii) hematopoiesis, (iii) tissue morphology, (iv) endocrine system development and function and (v) connective tissue development and function (Figure 5B). Other enriched categories are shown in Supplementary Table S11, involving TLX3 in cancer, intestinal, hematological and immunological diseases as well as in metabolism and energy production. Further details about enriched subcategories within the ‘physiological system development and function’ category are provided as a supplement in Supplementary Table S12.

## DISCUSSION

We introduced a novel structure-based computational algorithm, TF2DNA, for the prediction of TF binding preferences. The approach relies on the use of available TF-DNA complexes as template structures in model building, each of which were subjected to careful manual curation. While conformational changes in DNA also play an important role in determining TF specificity (61), this work advances our understanding of binding specificities by considering the protein structure. As opposed to other methods in the literature (19–24), TF2DNA scores the TF-DNA interface of models generated by fully enumerating DNA sequences. This can avoid the often used, but questionable, assumption of considering interactions in an additive manner (62,63). TF2DNA prediction accuracies fall in the range of 45–82%, depending primarily on target-template sequence identities (spanning the range of 0–100%), conservation of binding site environment and quality of TF-DNA models. The method is able to correctly predict DNA motifs that are very different from those observed using the protein sequence of the template complex (e.g. in the case of the correctly predicted SFP1 TF where the residue conservation of the template interface is 0%).

The main bottleneck of the method is finding suitable structure templates to generate high quality homology models for the target TF sequences. Reliable homology models could not be generated for 28% of the target sequences, due to the limited availability of TF-DNA complex structures in the PDB (including sequence variants of similar structures). However, we expect this problem to be alleviated in the near future, as suitable templates continue to emerge, given the rapid expansion of the PDB and the ongoing worldwide structural genomics efforts (64,65). Additional bottlenecks are associated with inaccurate TF modeling, which is especially aggravated in cases of water-mediated protein-DNA interactions (66,67), and incorrectly captured TF-DNA binding affinities, particularly due to the contributions of shape-readouts.

We compared the Robertson–Varani knowledge-based potential against the RosettaDNA *ab initio* potential for the description of TF-DNA interaction specificities. We found that the *ab initio* potential was not able to distinguish binding from decoy DNA sequences in any of the tested cases, whereas the knowledge-based potential was very precise at ranking TF binding sites. One possible explanation is that the Robertson–Varani potential was specifically developed and optimized to discriminate cognate from decoy sites, regardless of their precise affinity for the TF. On the other hand, RosettaDNA was created to measure affinity in protein-DNA interactions (42). Therefore, RosettaDNA always optimizes the protein-DNA interface to maximize its affinity, causing a loss of information about the specificity of the interaction because affinity and specificity seldom correlate in nucleic acid interactions (68).

When exploring a large-scale application of the method in the human genome we found that we can generate 1321 binding motifs out of the 1825 estimated human TF sequences (43). As an anecdotal verification we experimentally confirmed the binding motif for the TLX3 TF. Promoter searches with the TLX3 motif identified a set of putative human regulated genes that were used for functional characterization via network enrichment studies. The functional enrichment implicates TLX3 as functioning in hematopoiesis, tissue morphology, connective tissue function and endocrine system function. It is also associated to hematological and intestinal diseases and cancer. These functions are generally consistent with the tissue types in which TLX3 is also known to be expressed (reported in The Human Protein Atlas (60)), such as hematopoietic, endocrine and epithelial cells.

TLX3 function has been found to be related to the establishment of sensory neuron phenotypes (26,27). TLX3 is also involved in the development of T-ALL disease (58,59). The oncogenic T-ALL predicted transcriptional network of Della Gatta *et al.* shows TLX1 and TLX3 as master regulators and identify the protein RUNX1 (runt-related TF 1) as a tumor suppressor gene from ChIP-chip experiments. The RUNX1 TF is known to regulate hematopoietic development (69). Our list of TLX3 targets also predicts direct regulation of the *RUNX1* gene (rank 535). Another work showed that TLX3 is required for the acquisition of cholinergic phenotype in neurons during the prenatal development of the mouse (56). Furthermore, they demonstrate that TLX3 is required for the expression of the cholinergic peptide VIP and SST hormones. We find VIP (rank 39) in the list of TLX3 putative targets but not SST. Instead of SST, we find the SSTR2 (SST receptor 2) in the list (rank 187). This suggests TLX3 could directly regulate expression of VIP but not of SST. Alternatively, SST may be indirectly affected by SSTR2-mediated autoregulation, probably through the negative regulation of the STAT5A (signal transducer and activator of transcription 5) protein via SSTR2 signaling pathway (70), since we find STAT5A as a regulator of SST in our predictions.

The method described in this work is sufficiently general and can be applied to any set of TF sequences to investigate or supplement existent but incomplete regulatory networks for any organism of interest.

## SUPPLEMENTARY DATA

Supplementary Data are available at NAR Online.

SUPPLEMENTARY DATA

## References

[1] Ernst P., Smale S.T. (1995). Combinatorial regulation of transcription. I: general aspects of transcriptional control. Immunity.

[2] Slattery M., Zhou T., Yang L., Dantas Machado A.C., Gordan R., Rohs R. (2014). Absence of a simple code: how transcription factors read the genome. Trends Biochem. Sci..

[3] Stormo G.D., Zhao Y. (2010). Determining the specificity of protein-DNA interactions. Nat. Rev. Genet..

[4] Pujato M., MacCarthy T., Fiser A., Bergman A. (2013). The underlying molecular and network level mechanisms in the evolution of robustness in gene regulatory networks. PLoS Comput. Biol..

[5] MacCarthy T., Bergman A. (2007). The limits of subfunctionalization. BMC Evol. Biol..

[6] Bryne J., Valen E., Tang M., Marstrand T., Winther O., da Piedade I., Krogh A., Lenhard B., Sandelin A. (2008). JASPAR, the open access database of transcription factor-binding profiles: new content and tools in the 2008 update. Nucleic Acids Res..

[7] Newburger D., Bulyk M. (2009). UniPROBE: an online database of protein binding microarray data on protein-DNA interactions. Nucleic Acids Res..

[8] Matys V., Kel-Margoulis O.V., Fricke E., Liebich I., Land S., Barre-Dirrie A., Reuter I., Chekmenev D., Krull M., Hornischer K. (2006). TRANSFAC and its module TRANSCompel: transcriptional gene regulation in eukaryotes. Nucleic Acids Res..

[9] Quest D., Ali H. (2010). The Motif Tool Assessment Platform (MTAP) for sequence-based transcription factor binding site prediction tools. Methods Mol. Biol..

[10] Liu L.A., Bradley P. (2012). Atomistic modeling of protein-DNA interaction specificity: progress and applications. Curr. Opin. Struct. Biol..

[11] Bailey T.L., Elkan C. (1995). The value of prior knowledge in discovering motifs with MEME. Proc. Int. Conf. Intell. Syst. Mol. Biol..

[12] Liu X., Brutlag D.L., Liu J.S. (2001). BioProspector: discovering conserved DNA motifs in upstream regulatory regions of co-expressed genes. Pac. Symp. Biocomput..

[13] Liu X.S., Brutlag D.L., Liu J.S. (2002). An algorithm for finding protein-DNA binding sites with applications to chromatin-immunoprecipitation microarray experiments. Nat. Biotech..

[14] Nakaki R., Kang J., Tateno M. (2012). A novel ab initio identification system of transcriptional regulation motifs in genome DNA sequences based on direct comparison scheme of signal/noise distributions. Nucleic Acids Res..

[15] Pavesi G., Mauri G., Pesole G. (2001). An algorithm for finding signals of unknown length in DNA sequences. Bioinformatics.

[16] Smith A.D., Sumazin P., Zhang M.Q. (2005). Identifying tissue-selective transcription factor binding sites in vertebrate promoters. Proc. Natl. Acad. Sci. U.S.A..

[17] Gama-Castro S., Salgado H., Peralta-Gil M., Santos-Zavaleta A., Muñiz-Rascado L., Solano-Lira H., Jimenez-Jacinto V., Weiss V., García-Sotelo J.S., López-Fuentes A. (2011). RegulonDB version 7.0: transcriptional regulation of Escherichia coli K-12 integrated within genetic sensory response units (Gensor Units). Nucleic Acids Res..

[18] Narlikar L., Ovcharenko I. (2009). Identifying regulatory elements in eukaryotic genomes. Brief. Funct. Genom. Proteom..

[19] Alamanova D., Stegmaier P., Kel A. (2010). Creating PWMs of transcription factors using 3D structure-based computation of protein-DNA free binding energies. BMC Bioinformat..

[20] Alibés A., Nadra A.D., De Masi F., Bulyk M.L., Serrano L., Stricher F. (2010). Using protein design algorithms to understand the molecular basis of disease caused by protein-DNA interactions: the Pax6 example. Nucleic Acids Res..

[21] Angarica V.E., Pérez A.G., Vasconcelos A.T., Collado-Vides J., Contreras-Moreira B. (2008). Prediction of TF target sites based on atomistic models of protein-DNA complexes. BMC Bioinformat..

[22] Liu Z., Guo J.T., Li T., Xu Y. (2008). Structure-based prediction of transcription factor binding sites using a protein-DNA docking approach. Proteins.

[23] Morozov A.V., Havranek J.J., Baker D., Siggia E.D. (2005). Protein-DNA binding specificity predictions with structural models. Nucleic Acids Res..

[24] Morozov A.V., Siggia E.D. (2007). Connecting protein structure with predictions of regulatory sites. Proc. Natl. Acad. Sci. U.S.A..

[25] Dessailly B.H., Nair R., Jaroszewski L., Fajardo J.E., Kouranov A., Lee D., Fiser A., Godzik A., Rost B., Orengo C. (2009). PSI-2: structural genomics to cover protein domain family space. Structure.

[26] Kondo T., Matsuoka A.J., Shimomura A., Koehler K.R., Chan R.J., Miller J.M., Srour E.F., Hashino E. (2011). Wnt signaling promotes neuronal differentiation from mesenchymal stem cells through activation of Tlx3. Stem Cells.

[27] Lopes C., Liu Z., Xu Y., Ma Q. (2012). Tlx3 and Runx1 act in combination to coordinate the development of a cohort of nociceptors, thermoceptors, and pruriceptors. J. Neurosci..

[28] Bernstein F.C., Koetzle T.F., Williams G.J., Meyer E.F., Brice M.D., Rodgers J.R., Kennard O., Shimanouchi T., Tasumi M. (1977). The Protein Data Bank: a computer-based archival file for macromolecular structures. J. Mol. Biol..

[29] Li W., Jaroszewski L., Godzik A. (2001). Clustering of highly homologous sequences to reduce the size of large protein databases. Bioinformatics.

[30] Söding J. (2005). Protein homology detection by HMM-HMM comparison. Bioinformatics.

[31] Sali A., Blundell T.L. (1993). Comparative protein modelling by satisfaction of spatial restraints. J. Mol. Biol..

[32] Phillips J.C., Braun R., Wang W., Gumbart J., Tajkhorshid E., Villa E., Chipot C., Skeel R.D., Kale L., Schulten K. (2005). Scalable molecular dynamics with NAMD. J. Comput. Chem..

[33] Robertson T., Varani G. (2007). An all-atom, distance-dependent scoring function for the prediction of protein-DNA interactions from structure. Proteins.

[34] Schones D.E., Sumazin P., Zhang M.Q. (2005). Similarity of position frequency matrices for transcription factor binding sites. Bioinformatics.

[35] Felsenstein J. (1989). PHYLIP - Phylogeny Inference Package (Version 3.2). Cladistics.

[36] Eschenfeldt W.H., Lucy S., Millard C.S., Joachimiak A., Mark I.D. (2009). A family of LIC vectors for high-throughput cloning and purification of proteins. Methods Mol. Biol..

[37] Studier F.W. (2005). Protein production by auto-induction in high density shaking cultures. Protein Expr. Purif..

[38] Pruitt K.D., Tatusova T., Brown G.R., Maglott D.R. (2012). NCBI Reference Sequences (RefSeq): current status, new features and genome annotation policy. Nucleic Acids Res..

[39] Bailey T.L., Gribskov M. (1998). Combining evidence using p-values: application to sequence homology searches. Bioinformatics.

[40] Fernandez-Fuentes N., Madrid-Aliste C.J., Rai B.K., Fajardo J.E., Fiser A. (2007). M4T: a comparative protein structure modeling server. Nucleic Acids Res..

[41] Kauffman C., Karypis G. (2012). Computational tools for protein–DNA interactions. Wiley Interdisciplinary Rev. Data Mining Knowledge Discov.

[42] Havranek J.J., Duarte C.M., Baker D. (2004). A simple physical model for the prediction and design of protein-DNA interactions. J. Mol. Biol..

[43] Vaquerizas J.M., Kummerfeld S.K., Teichmann S.A., Luscombe N.M. (2009). A census of human transcription factors: function, expression and evolution. Nat. Rev. Genet..

[44] Jolma A., Yan J., Whitington T., Toivonen J., Nitta K.R., Rastas P., Morgunova E., Enge M., Taipale M., Wei G. (2013). DNA-binding specificities of human transcription factors. Cell.

[45] Kulakovskiy I.V., Medvedeva Y.A., Schaefer U., Kasianov A.S., Vorontsov I.E., Bajic V.B., Makeev V.J. (2013). HOCOMOCO: a comprehensive collection of human transcription factor binding sites models. Nucleic Acids Res..

[46] Gray K.A., Daugherty L.C., Gordon S.M., Seal R.L., Wright M.W., Bruford E.A. (2013). Genenames.org: the HGNC resources in 2013. Nucleic Acids Res..

[47] Fulton D.L., Sundararajan S., Badis G., Hughes T.R., Wasserman W.W., Roach J.C., Sladek R. (2009). TFCat: the curated catalog of mouse and human transcription factors. Genome Biol..

[48] Pfreundt U., James D.P., Tweedie S., Wilson D., Teichmann S.A., Adryan B. (2010). FlyTF: improved annotation and enhanced functionality of the Drosophila transcription factor database. Nucleic Acids Res..

[49] Reece-Hoyes J.S., Deplancke B., Shingles J., Grove C.A., Hope I.A., Walhout A.J. (2005). A compendium of Caenorhabditis elegans regulatory transcription factors: a resource for mapping transcription regulatory networks. Genome Biol..

[50] Harbison C.T., Gordon D.B., Lee T.I., Rinaldi N.J., Macisaac K.D., Danford T.W., Hannett N.M., Tagne J.B., Reynolds D.B., Yoo J. (2004). Transcriptional regulatory code of a eukaryotic genome. Nature.

[51] Pérez-Rueda E., Collado-Vides J. (2000). The repertoire of DNA-binding transcriptional regulators in Escherichia coli K-12. Nucleic Acids Res..

[52] Satoh J., Tabunoki H. (2013). A comprehensive profile of ChIP-Seq-based STAT1 target genes suggests the complexity of STAT1-mediated gene regulatory mechanisms. Gene Regul. Syst. Bio..

[53] Satoh J., Kawana N., Yamamoto Y. (2013). Pathway analysis of ChIP-Seq-based NRF1 target genes suggests a logical hypothesis of their involvement in the pathogenesis of neurodegenerative diseases. Gene Regul. Syst. Bio..

[54] Cheng C., Min R., Gerstein M. (2011). TIP: a probabilistic method for identifying transcription factor target genes from ChIP-seq binding profiles. Bioinformatics.

[55] Teytelman L., Thurtle D.M., Rine J., van Oudenaarden A. (2013). Highly expressed loci are vulnerable to misleading ChIP localization of multiple unrelated proteins. Proc. Natl. Acad. Sci. U.S.A..

[56] Huang T., Hu J., Wang B., Nie Y., Geng J., Cheng L. (2013). Tlx3 controls cholinergic transmitter and Peptide phenotypes in a subset of prenatal sympathetic neurons. J. Neurosci..

[57] Bernard O.A., Busson-LeConiat M., Ballerini P., Mauchauffé M., Della Valle V., Monni R., Nguyen Khac F., Mercher T., Penard-Lacronique V., Pasturaud P. (2001). A new recurrent and specific cryptic translocation, t(5;14)(q35;q32), is associated with expression of the Hox11L2 gene in T acute lymphoblastic leukemia. Leukemia.

[58] Dadi S., Le Noir S., Payet-Bornet D., Lhermitte L., Zacarias-Cabeza J., Bergeron J., Villarèse P., Vachez E., Dik W.A., Millien C. (2012). TLX homeodomain oncogenes mediate T cell maturation arrest in T-ALL via interaction with ETS1 and suppression of TCRα gene expression. Cancer Cell.

[59] Della Gatta G., Palomero T., Perez-Garcia A., Ambesi-Impiombato A., Bansal M., Carpenter Z.W., De Keersmaecker K., Sole X., Xu L., Paietta E. (2012). Reverse engineering of TLX oncogenic transcriptional networks identifies RUNX1 as tumor suppressor in T-ALL. Nat. Med..

[60] Uhlen M., Oksvold P., Fagerberg L., Lundberg E., Jonasson K., Forsberg M., Zwahlen M., Kampf C., Wester K., Hober S. (2010). Towards a knowledge-based Human Protein Atlas. Nat. Biotech..

[61] Yang L., Zhou T., Dror I., Mathelier A., Wasserman W.W., Gordan R., Rohs R. (2014). TFBSshape: a motif database for DNA shape features of transcription factor binding sites. Nucleic Acids Res..

[62] Benos P.V., Bulyk M.L., Stormo G.D. (2002). Additivity in protein-DNA interactions: how good an approximation is it?. Nucleic Acids Res..

[63] Maerkl S.J., Quake S.R. (2007). A systems approach to measuring the binding energy landscapes of transcription factors. Science.

[64] Montelione G.T. (2012). The Protein Structure Initiative: achievements and visions for the future. F1000 Biol. Rep..

[65] Khafizov K., Madrid-Aliste C., Almo S.C., Fiser A. (2014). Trends in structural coverage of the protein universe and the impact of the Protein Structure Initiative (vol 111, pg 3733, 2014). Proc. Natl. Acad. Sci. U.S.A..

[66] Jayaram B., Jain T. (2004). The role of water in protein-DNA recognition. Annu. Rev. Biophys. Biomol. Struct..

[67] Pabo C.O., Sauer R.T. (1992). Transcription factors: structural families and principles of DNA recognition. Annu. Rev. Biochem..

[68] Demidov V.V., Frank-Kamenetskii M.D. Two sides of the coin: affinity and specificity of nucleic acid interactions. Trends Biochem. Sci..

[69] Okuda T., Nishimura M., Nakao M., Fujita Y. (2001). RUNX1/AML1: a central player in hematopoiesis. Int. J. Hematol..

[70] Theodoropoulou M., Stalla G.K. (2013). Somatostatin receptors: from signaling to clinical practice. Front Neuroendocrinol..

[71] Sippl M.J. (1993). Recognition of errors in three-dimensional structures of proteins. Proteins.

