# Supplementary material for: Prediction of DNA binding motifs from 3D models of transcription factors; identifying TLX3 regulated genes
Source: Nucleic Acids Res. 2014 Nov 26;42(22):13500–12. doi: 10.1093/nar/gku1228 (PMC4267649; doi:10.1093/nar/gku1228)
Supplement: SUPPLEMENTARY DATA [file supp_42_22_13500__index.html]

Prediction of DNA binding motifs from 3D models of transcription factors; identifying TLX3 regulated genes — Prediction of DNA binding motifs from 3D models of transcription factors; identifying TLX3 regulated genes — SUPPLEMENTARY DATA 

# Prediction of DNA binding motifs from 3D models of transcription factors; identifying TLX3 regulated genes

## SUPPLEMENTARY DATA

**Files in this Data Supplement:**

- SUPPLEMENTARY DATA
- SUPPLEMENTARY DATA
