# Supplementary material for: Prediction of DNA binding motifs from 3D models of transcription factors; identifying TLX3 regulated genes
Source: Nucleic Acids Res. 2014 Nov 26;42(22):13500–12. doi: 10.1093/nar/gku1228 (PMC4267649; doi:10.1093/nar/gku1228)
Supplement: SUPPLEMENTARY DATA [file supp_gku1228_nar-02093-z-2014-File007.docx]

### Supplementary figures


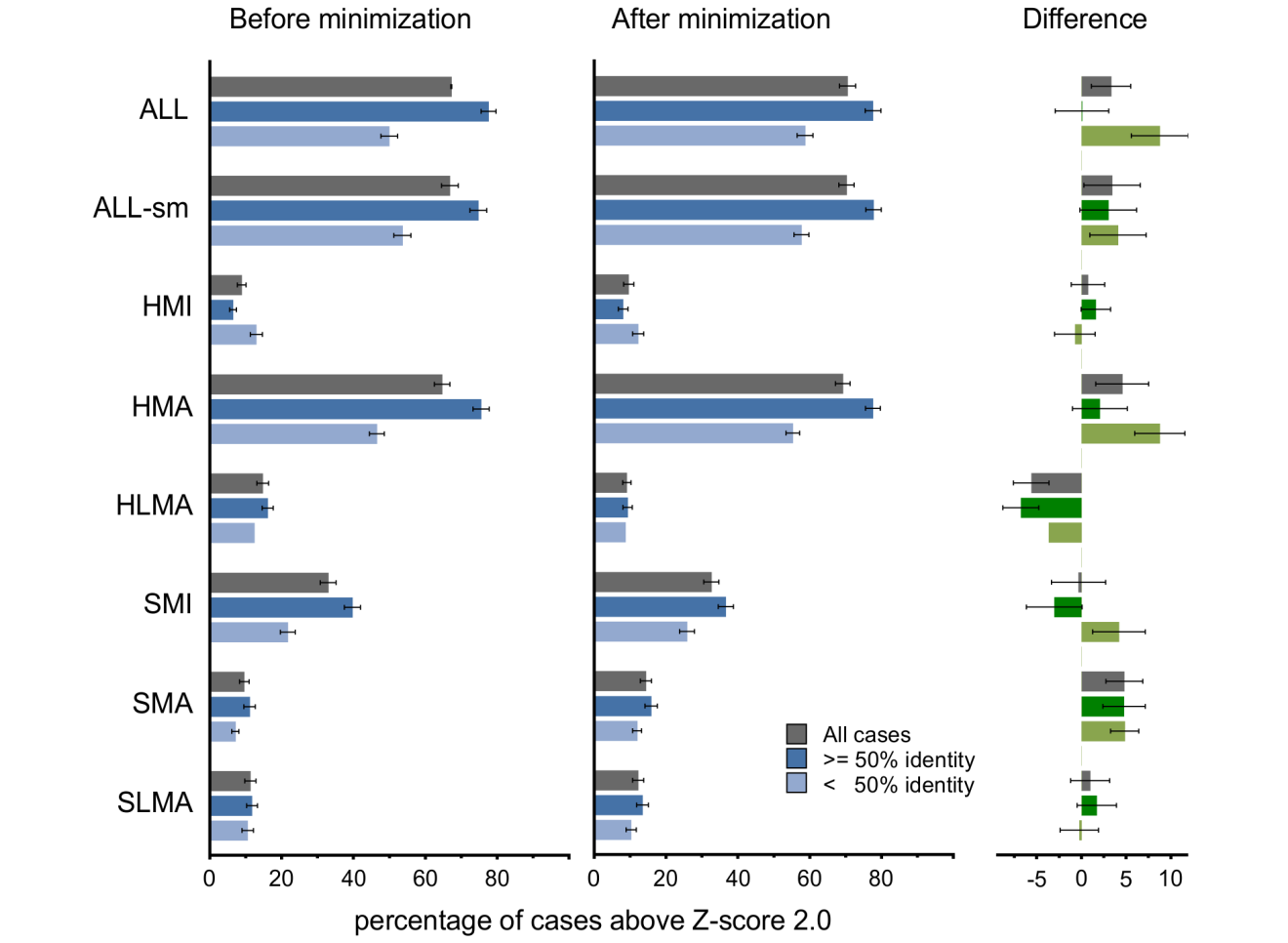


**Figure S1. TF2DNA performance under diverse conditions**

Performance of the TF2DNA method as the percent of cases above a Z-score of 2.0 (95% confidence) before and after structural relaxation (energy minimization with CHARMM). Several training schemes were considered (**Table S2** has details of the training schemes). The third panel shows the difference between the performances after and before minimization, where positive values indicate a gain after minimization. Gray bars denote performances of the full set, blue and light blue bars correspond to subsets containing cases with target-template sequence identities ≥50% and <50%, respectively. The subset with <50% target-template sequence identity is enriched in cases that are difficult to align and model, explaining variations in that subset upon structural relaxation. Error bars are the standard deviation from evaluating random samples of 20 cases, 20 times.

**
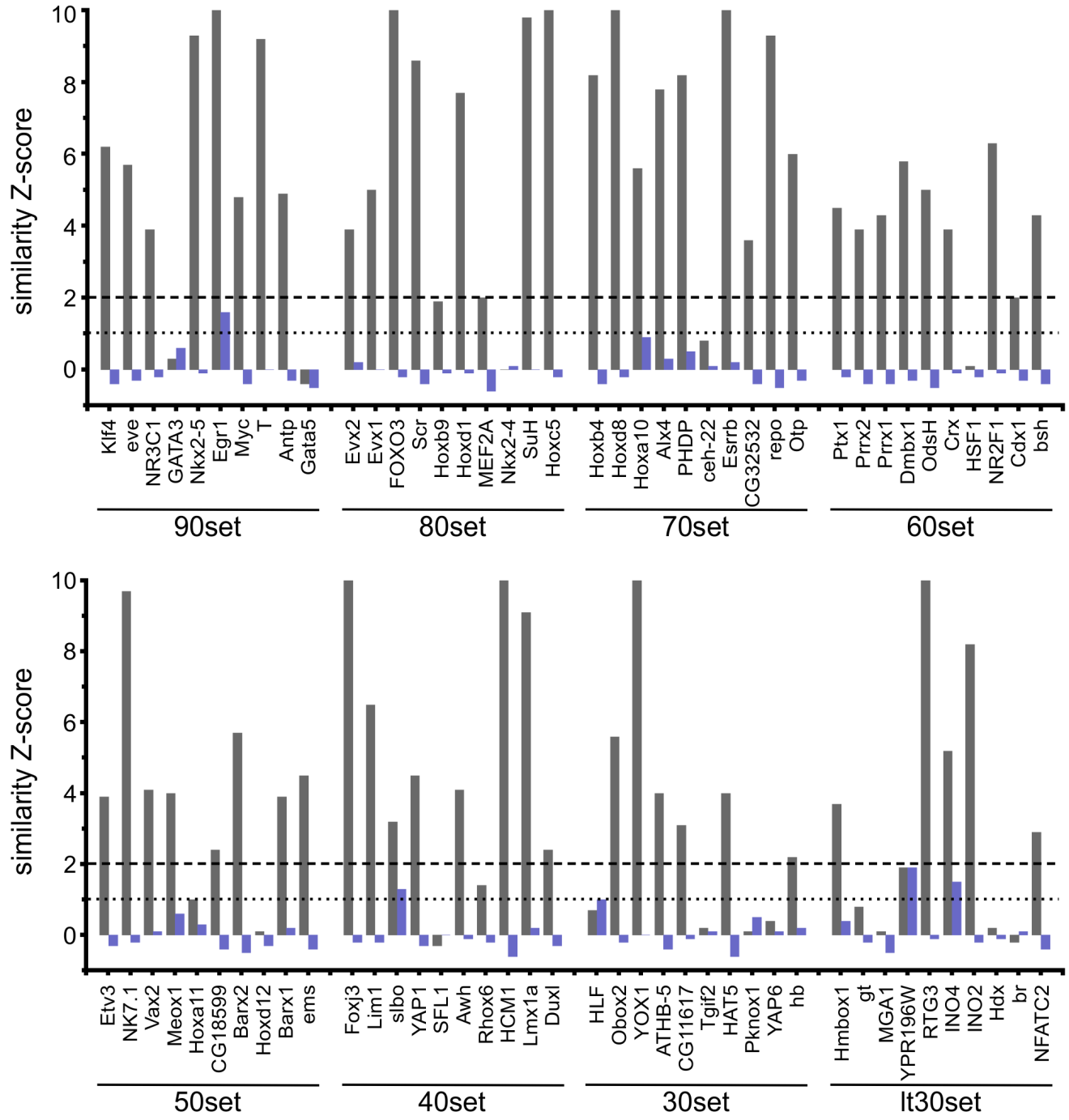
**

**Figure S2. Performance of the RosettaDNA potential**

Similarity Z-scores of motif predictions using the Robertson-Varani knowledge-based potential (gray bars) and the RosettaDNA potential (blue bars) for the 80 randomly chosen cases from the test set of 311 TF sequences. The 80 cases are equally distributed among the 8 target-template identity bins. Z-scores of 1 and 2 correspond to confidence levels in motif similarity of 66% and 95%, displayed with dotted and dashed lines, respectively.

**
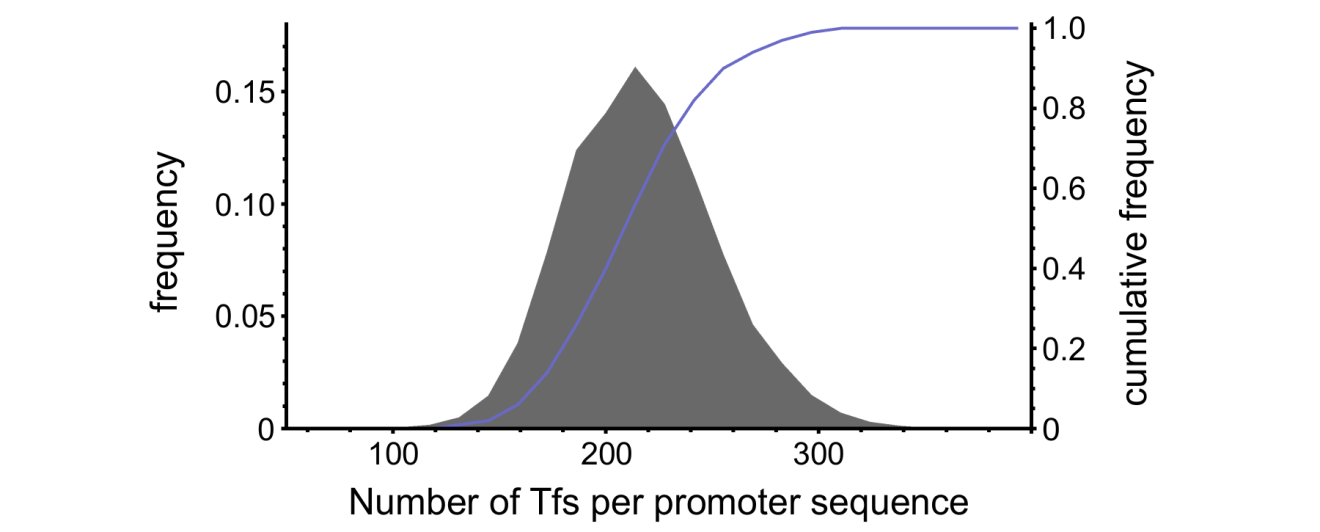
**

**Figure S3. Number of TFs per promoter sequence**

The gray area displays the distribution of the number of TFs that regulate a promoter (it is not “per gene” because some genes have more than one promoter). The minimum and maximum values are 76 and 396 TFs per promoter, respectively. The median is at 215 TFs per promoter sequence. The blue line represents the corresponding cumulative distribution function.

**
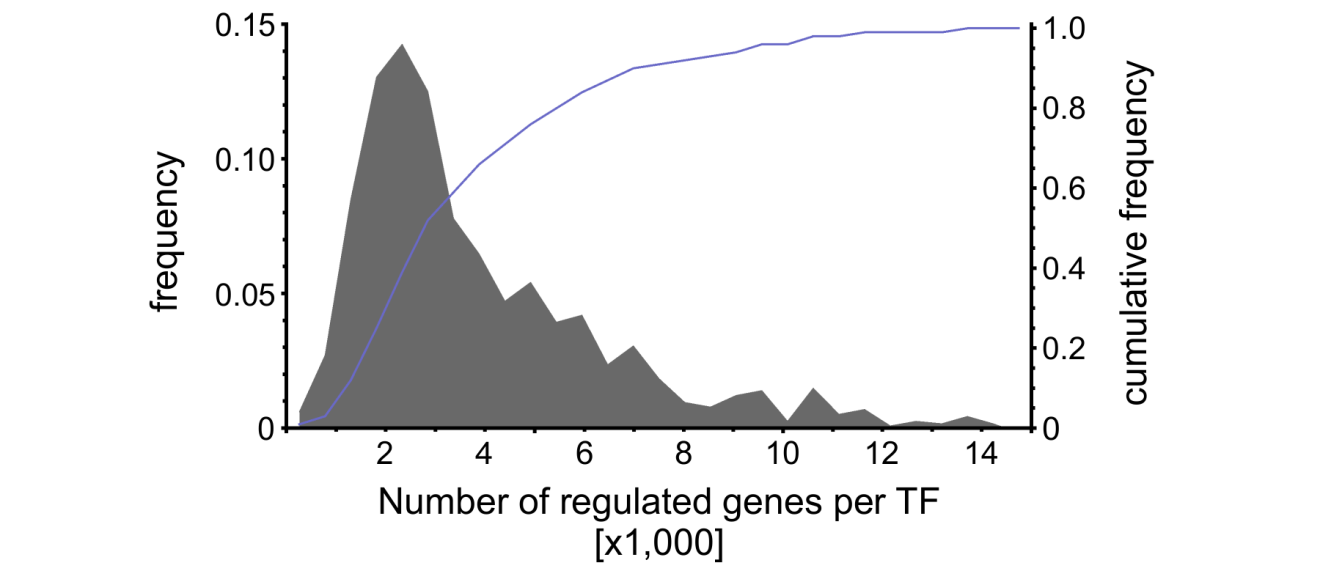
**

**Figure S4. Number of regulated genes per TF**

Distribution of the predicted number of regulated genes per TF in the TF2DNA database (gray area). The minimum and maximum values are 244 and 14,435 genes per TF, respectively. The distribution is highly skewed with the median at 3,026 regulated genes per TF (mode at 2,327). The blue line represents the cumulative distribution function.


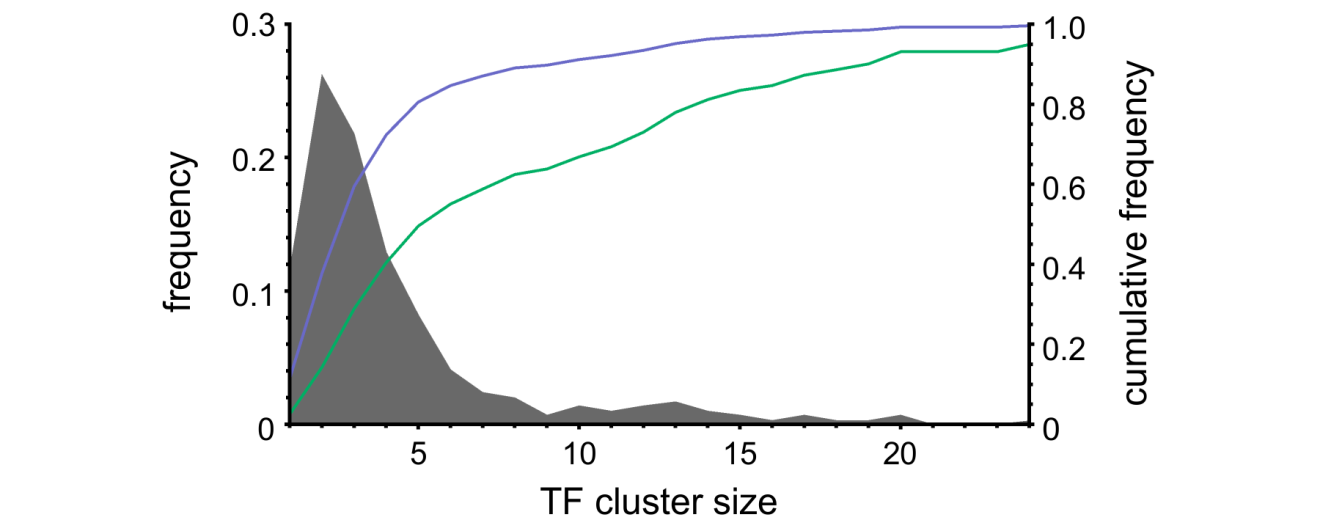


**Figure S5. Clustering of TF binding motifs.**

Distribution of cluster sizes for a similarity Z-score cutoff of 2.0 (grey area). Clusters with sizes 25 through 66 were left out of the figure. All those clusters have no occurrences, except cluster 66, which has a population of one. The blue line represents the cumulative distribution function. The green line is the cumulative distribution function of the percentage of TFs found in each cluster size bin. Clusters with sizes 5 or less cover 80% of all 294 clusters (blue line). Nevertheless, these clusters cover only 49.6% of all TF binding motifs (green line). In other words, most clusters have small sizes and most TFs are part of big clusters.

### Supplementary tables

**Table S1. List of TF-DNA structural complexes used as templates for homology modeling of TFs**

A manually curated a set of 171 TF-DNA structural complexes was collected from the PDB. The structures included transcription factors bound to double stranded DNA, consisting at most 9 base pairs. Crystal water molecules were retained. Refer to **Table S2** for keys to column 6 codes.

| **Number** | **PDB code** | **Name** | **DNA length** | **Canonical sequence** | **Binding**  **mode** |
| --- | --- | --- | --- | --- | --- |
| 1 | 1A3Q | NF-KAPPA-B P52 | 8 | GAATCCCC | SLMA |
| 2 | 1A6Y | REVERBA orphan nuclear receptor | 9 | ACTAGGTCA | HLMA |
| 3 | 1AIS | TATA-binding protein | 9 | CTTTTTAAA | SMI |
| 4 | 1AKH | MAT alpha2 | 8 | ATGTAAAA | HMA |
| 5 | 1AM9 | SREBP-1A | 9 | GTGGGGTGA | HMA |
| 7 | 1AWC | GABP alpha/beta domain | 8 | CCGGAAGT | HMA |
| 8 | 1B3T | EBNA-1 nuclear protein | 9 | ATGCTTCCC | HMA |
| 9 | 1B72 | HOX-B1 | 8 | TGATCGGC | HMA |
| 10 | 1B72 | PBX1 | 8 | TATGATTG | HMA |
| 11 | 1B8I | UBX | 8 | GCCATAAA | HMA |
| 12 | 1BC7 | SAP-1 | 9 | ACAGGATGT | HMA |
| 13 | 1BDT | ARC | 9 | CTTCTATCA | SMA |
| 14 | 1BF4 | SSO7D | 7 | GCGTTCG | SMI |
| 15 | 1BG1 | STAT3B | 8 | TTTCCCGT | SLMA |
| 18 | 1DSZ | RARA | 8 | CAGGTCAA | HLMA |
| 19 | 1DSZ | RXRA | 8 | AAGGTCAG | HLMA |
| 20 | 1DUX | ELK-1 | 8 | CCGGAAGT | HMA |
| 21 | 1E3O | OCT-1 POU dimer | 9 | ATGCATGAG | HMA |
| 22 | 1F2I | ZIF12 (zinc fingers 1 AND 2 of ZIF268) | 8 | GCGCCCAT | HLMA |
| 23 | 1F4K | Replication terminator protein | 8 | ATGTTCAT | HMA |
| 24 | 1FJL | paired protein | 8 | CAGATTAT | HMA |
| 25 | 1G2D | CYS2HIS2 zinc finger | 9 | GCTATAAAA | HMA |
| 26 | 1GD2 | PAP1 | 7 | CGTAACC | HMA |
| 27 | 1GTW | C/EBP beta | 7 | CGCAATC | HMA |
| 28 | 1GXP | PHOB | 9 | GTCATAAAG | HMA |
| 29 | 1H6F | TBX3 | 9 | TTCACACCT | SLMA |
| 30 | 1H89 | C-Myb | 9 | TAACGGACT | HMA |
| 31 | 1HCQ | Estrogen receptor | 8 | GTGACCTG | HMA |
| 34 | 1IG7 | MSX-1 | 9 | CTAATTGAA | HMA |
| 35 | 1IGN | RAP1_A | 9 | GCACACCCA | HLMA |
| 36 | 1IGN | RAP1_B | 8 | CACACCAG | HLMA |
| 37 | 1IHF | IHF alpha/beta | 6 | GCAACA | SMI |
| 38 | 1J1V | DnaA domainIV | 9 | TTATCCACA | HMA |
| 39 | 1JE8 | Nitrate/Nitrite response regulator protein NARL | 8 | GTACCCAT | HMA |
| 40 | 1JFT | PurR | 8 | CGCAAACG | HMA |
| 41 | 1JGG | Even-skipped Homeodomain | 7 | TTGAATT | HMA |
| 42 | 1JJ4 | E2 | 7 | CAACCGA | HMA |
| 43 | 1K78 | Pax5 | 9 | CTCCAGTGG | HMA |
| 44 | 1K79 | Ets-1 | 9 | CCGGAAATG | HMA |
| 45 | 1KU7 | SigmaA | 7 | CTTGACA | HMA |
| 46 | 1L3L | TraR | 8 | TCTGCACA | HMA |
| 47 | 1LAT | Glucocorticoid receptor | 8 | CAGAACAT | HMA |
| 48 | 1LE8 | MATa1 | 8 | TTACATCA | HMA |
| 49 | 1LLI | Repressor protein CI | 8 | TACCACTG | HMA |
| 50 | 1LLM | Zif23-GCN4 Chimera | 8 | CCCACGCG | HMA |
| 51 | 1LQ1 | Stage 0 sporulation protein A | 8 | GTGTCGAA | HMA |
| 52 | 1MEY | Designed zinc finger | 9 | GAGGCAGAA | HMA |
| 53 | 1MJ2 | Met represssor | 9 | AGACTGCTA | SMA |
| 54 | 1MNM | MATalpha2 | 8 | ATTACCTA | HMA |
| 55 | 1MNM | Mcm1 | 7 | ATTACCT | HMA |
| 56 | 1N6J | MEF2 | 8 | CTATTTAT | SMI |
| 57 | 1NFK | NF-KB | 8 | GAATTCCC | SLMA |
| 58 | 1NKP | Myc | 8 | CACGTGCT | HMA |
| 59 | 1NKP | Max | 8 | ACGTGCTA | HMA |
| 60 | 1NLW | Mad | 8 | ACGTGCTA | HMA |
| 61 | 1NVP | TBP | 9 | CTATAAAAG | SMI |
| 62 | 1OZJ | SMAD 3 | 8 | CTAGACAT | SMA |
| 64 | 1PDN | PRD paired | 9 | GTCACGGTT | HMA |
| 65 | 1PER | 434 repressor | 9 | TTTCTTGTA | HMA |
| 66 | 1PP7 | initiator binding protein | 9 | GTTACTTCA | HMA |
| 67 | 1PUE | PU.1 | 9 | AGGGGAAGT | HMA |
| 68 | 1PUF | HoxA9 | 8 | TTTACGAC | HMA |
| 69 | 1QNE | TBP | 8 | TATAAAAG | SMI |
| 70 | 1QPI | Tetracycline repressor | 6 | CTATCA | HLMA |
| 71 | 1R71 | korB | 8 | GGCTAAAA | HMA |
| 73 | 1R8E | BmrR | 9 | ACCCTCCCC | HMA |
| 74 | 1RIO | bacteriophage lambda cI-NTD | 9 | TATCACCGC | HLMA |
| 75 | 1RM1 | TBP | 9 | ATATAAAAC | SMI |
| 76 | 1SKN | SKN-1 | 8 | TGTCATCC | HMA |
| 78 | 1TRR | TRP repressor | 7 | AGCGTAC | HLMA |
| 79 | 1UBD | YY1 | 9 | CTCCATTTT | HMA |
| 80 | 1W0T | HTRF1 | 9 | TAGGGTTAG | HMA |
| 81 | 1W0U | HTRF2 | 8 | TAGGGTTA | HMA |
| 82 | 1WD0 | Sac7d | 7 | TATATAG | SMI |
| 83 | 1XBR | T domain | 9 | TTCACACCT | SLMA |
| 84 | 1YO5 | PDEF | 9 | AGCAGGATG | HMA |
| 86 | 1ZNS | ColE7 | 9 | GGGATATCC | HMA |
| 87 | 1ZRF | alkaline phosphatase | 7 | ATGCGAT | HLMA |
| 88 | 1ZS4 | Repressor protein CII | 8 | CGTTGCGT | HLMA |
| 89 | 2A07 | Foxp2 | 8 | AACAAATT | HMA |
| 90 | 2A66 | hLRH-1 | 9 | TCAAGGCCA | HLMA |
| 91 | 2BNW | omega repressor | 7 | TGTGATT | SMA |
| 92 | 2BOP | E2 | 7 | TCGGTCG | HMA |
| 94 | 2C7A | Progesterone receptor | 7 | CAGAACA | HMA |
| 95 | 2C9L | BZLF1 | 8 | GACTCATG | HMA |
| 96 | 2D5V | HNF-6alpha | 9 | AAGTCAATA | HMA |
| 97 | 2DGC | putative transcriptional regulator SCO5550 | 8 | ACGTCATC | HMA |
| 98 | 2E1C | Putative HTH-type transcriptional regulator PH1519 | 8 | TTTTCACT | HMA |
| 99 | 2ETW | Ndt80 | 9 | CCACACAAA | SLMA |
| 100 | 2H1K | Pdx1 | 8 | CTCATTAG | HMA |
| 101 | 2H27 | SigmaE Region 4 | 7 | CGGAACT | HMA |
| 102 | 2H7H | JUN | 8 | ACTCATCG | HMA |
| 103 | 2HAN | Protein ultraspiracle | 8 | GGGTTCAA | HMA |
| 104 | 2HAN | Ecdysone receptor | 8 | AATGCACT | HMA |
| 105 | 2HAP | HAP1 | 8 | TCGCTATT | HLMA |
| 106 | 2HOS | Engrailed homeodomain | 8 | CGTAATCC | HMA |
| 107 | 2I06 | Tus | 9 | GTTGTAACT | SMA |
| 110 | 2IRF | IRF-2 | 8 | AGTGAAAG | HMA |
| 111 | 2ISZ | IdeR | 8 | TGTTAGCA | HMA |
| 112 | 2NLL | Thyroid hormone receptor | 8 | TCAGGTCA | HMA |
| 113 | 2NTC | large T antigen | 8 | TGGCCTCT | SLMA |
| 114 | 2O49 | CUT domain of SATB1 | 8 | ATATTAGC | HMA |
| 115 | 2OG0 | Lambda Xis | 9 | GTAGTCTGT | HLMA |
| 116 | 2PI0 | IRF-3 | 9 | TTTCAGTTT | HMA |
| 117 | 2QL2 | E47 | 8 | AGATGGCC | HMA |
| 119 | 2RAM | NF-KB P65 | 8 | ATTTCCAG | SLMA |
| 120 | 2RBF | PutA | 9 | GGTTGCACC | SLMA |
| 121 | 2VE9 | Gamma domain of FTSK | 8 | CCTGCCCT | HLMA |
| 123 | 2VY1 | LEAFY | 8 | TGGTCCGT | HMA |
| 124 | 2W7N | KORA | 8 | GCTAAACA | HMA |
| 125 | 2WBS | KLF4 | 7 | GCGCCTC | HLMA |
| 126 | 2WT7 | MAFB | 8 | ACTCATAG | HMA |
| 127 | 2WT7 | CFOS | 8 | TGCTGACT | HMA |
| 128 | 2X6V | TBX5 | 9 | AGGTGTGAG | SLMA |
| 129 | 2XSD | OCT-6 (POU3F1) | 9 | ATGCATGAG | HMA |
| 130 | 2YVH | CgmR | 8 | GGTCGGTA | HMA |
| 131 | 2Z3X | Small, acid-soluble spore protein C | 8 | GGGGGGGG | HMI |
| 132 | 3A46 | viral NEIL1 ortholog | 8 | CCAGTCTA | SLMA |
| 133 | 3BRD | CSL (Lag-1) | 9 | TTTCCCACA | SLMA |
| 134 | 3CMY | Pax-3 | 6 | ATTATG | HMA |
| 135 | 3CO6 | FoxO1 | 8 | GGTAAACA | HMA |
| 136 | 3COQ | Gal4 | 8 | CCGGAGGA | HLMA |
| 137 | 3CRO | CRO | 8 | TTCTTGTA | HMA |
| 138 | 3DPG | SgrAI | 8 | CGACCGGT | HMA |
| 139 | 3E6C | CprK | 7 | GTTAATG | HMA |
| 140 | 3ERE | Arginine repressor | 8 | TTATGCAA | HMA |
| 141 | 3EXJ | p53 | 8 | GCATGCTC | HLMA |
| 142 | 3FDQ | MogR | 8 | AAAAAAAT | HMA |
| 143 | 3G6P | Glucocorticoid receptor | 8 | CAGAACAC | HMA |
| 144 | 3G73 | FOXM1 | 8 | TGTTTATA | HMA |
| 145 | 3GNA | RAG1 | 9 | GTTTTTGTT | HMA |
| 146 | 3GPX | MutM Interrogation Complex 4 | 8 | CCGAGTCT | HMI |
| 147 | 3GXQ | ArtA | 8 | CATGACAT | SMA |
| 148 | 3H0D | CtsR | 9 | ATAGTCAAA | HMA |
| 149 | 3HTS | HSF | 7 | GTTCTAG | HMA |
| 151 | 3IAG | CSL (RBP-Jk) | 8 | TTCACACG | SLMA |
| 152 | 3IGM | PF14_0633 protein | 7 | TGCATGC | SMA |
| 153 | 3IKT | Redox-sensing transcriptional repressor rex | 9 | TTCACAGCG | HMA |
| 154 | 3JSO | LexA | 8 | TATACAGT | HMA |
| 155 | 3JTG | Elf3 | 9 | AACAGGAAA | HMA |
| 156 | 3JXB | Repressor protein C2 | 8 | TCTTAAAT | HMA |
| 157 | 3KET | Redox-sensing transcriptional repressor rex | 8 | TTCACAAT | HMA |
| 158 | 3KMD | p53 | 7 | GGCATGC | HMA |
| 159 | 3L2C | FOXO4 | 8 | TGTAAACA | HMA |
| 160 | 3LNQ | Aristaless | 8 | CTAATTAA | HMA |
| 162 | 3O9X | MqsA | 8 | TAGGTTAT | HMA |
| 165 | 3TS8 | Cellular tumor suppressor P53 | 6 | ACATGT | HLMA |
| 166 | 4A0A | TBX1 | 9 | GGTGTGAAA | SLMA |
| 167 | 4JL3 | Transcriptional regulator MS6564, TETR family | 8 | TCGTCTTG | HLMA |
| 168 | 3RKQ | Homeobox protein NKX-2.5 | 9 | TGAAGTGGG | HMA |
| 169 | 3QYN | Tumor protein 63 | 8 | ACATGTTT | HLMA |
| 170 | 3QWS | N15 CRO repressor protein | 7 | GCTATAA | HMA |
| 171 | 3PVP | Chromosomal replication initiator protein DNAA | 9 | TTATCCACA | HMA |
| 172 | 1VTN | HNF-3/fork head DNA-recognition motif | 9 | TAAGTCAAC | HMA |
| 173 | 4JBM | Interferon-inducible protein AIM2 | 6 | CGCGCC | HLMA |
| 174 | 4IX7 | INSV-BEN domain | 9 | CAATTGGAA | HMA |
| 175 | 4I2O | FIXK2 protein | 8 | AATTGATT | HMA |
| 176 | 4HQE | Transcriptional regulator QSRR | 7 | GGTATAA | HLMA |
| 177 | 4HN5 | Glucocorticoid receptor | 8 | CGGGAGAG | HMA |
| 178 | 4HC9 | GATA transcription factor-complex 3 | 7 | AAATCAG | HLMA |
| 179 | 4GZN | ZFP57 zinc finger | 8 | TTGCCGCA | HLMA |
| 180 | 4F6M | KAISO zinc finger | 8 | TCCTGCCA | HLMA |
| 181 | 4BQA | Protein C-ETS-2 | 8 | CCGGAAGT | HMA |
| 182 | 4AIJ | Transcriptional regulator SLYA | 8 | ATTATATT | HLMA |
| 183 | 3ZP5 | DNA binding domain of the human protein FEV | 8 | CACTTCCG | HMA |
| 184 | 3VOK | Transcriptional regulator HRTR | 7 | ATGACAC | HLMA |
| 185 | 3UK3 | Zinc finger protein 217 | 9 | GATTCTGCA | HLMA |
| 186 | 3U2B | SOX4 HMG domain | 8 | CTATTGTC | HMI |
| 187 | 3TMM | Transcription factor a, mitochondrial | 9 | GTTAGTTGG | HMI |
| 188 | 3TED | Chromo domain-containing protein 1 | 8 | ATATATGC | HLMA |
| 189 | 3S8Q | R-M controller protein | 7 | TGTGACT | HLMA |

**Table S2. Composition of TF-DNA structures according to binding mode**

The database of template structures have been qualitatively stratified (by visual inspection) according to the binding mode of the TF protein to the DNA molecule into 6 classes: HMA (helix binding the major groove), HLMA (helix/loop combination binding the major groove), SLMA (strand/loop combination binding the major groove), SMI (strands binding the minor groove), SMA (strands binding the major groove) and HMI (helix binding the minor groove). The 2^nd^ column contains the analysis on the database of template structures. Here, 93.0% of complexes are major groove binders, from which 80.7% use helices. Only a 7.0% recognize DNA through the minor groove using either helices or strands. See **Table S1** for specific examples of complexes within each category. The 4^th^ column shows counts for the structures in the control set, which adopt the binding mode of the template used. There are no minor groove binders in the set. Approximately 90% of the 311 modeled structures bind with helices to the major groove of the DNA. The rest contacts the DNA major groove with helix/loop and strand/loop mixtures.

| **Binding mode code** | **Structures in database** | **Percentage in database** | **Structures in**  **control set** | **Percentage in control set** |
| --- | --- | --- | --- | --- |
| **HMA** | 109 | 63.7 | 278 | 89.4 |
| **HLMA** | 29 | 17.0 | 23 | 7.4 |
| **SLMA** | 14 | 8.2 | 8 | 2.6 |
| **SMI** | 8 | 4.7 | 2 | 0.6 |
| **SMA** | 7 | 4.1 | 0 | 0.0 |
| **HMI** | 4 | 2.3 | 0 | 0.0 |
| total | 171 | 100 | 311 | 100 |

**Table S3. Performance of the control set**

The control set is composed of 11 transcription factors for which there was a perfectly matching experimental motif in both JASPAR and UniPROBE databases. The first column shows the Z-score similarity between predicted and experimental motifs. The second shows the Z-score similarity between JASPAR and UniPROBE experimental motifs. Higher values show higher confidence. The third column displays TF2DNA predicted motifs and the fourth column the binding motifs from the UniPROBE database (by definition, JASPAR motifs are the same).

| **TF name** | **Z-score** | **JU Z-score** | **TF2DNA motif** | **DB motif** |
| --- | --- | --- | --- | --- |
| PBX1 | 7.7 | 10.0 | 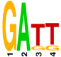 | 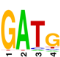 |
| ELK1 | 6.5 | 4.8 | 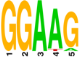 | 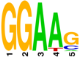 |
| ELK4 | 10 | 10.0 | 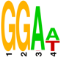 | 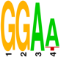 |
| Gabpa | 5.1 | 8.9 | 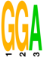 | 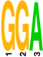 |
| RAP1 | 4.2 | 9.9 | 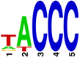 | 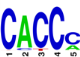 |
| GAL4 | 1.9 | 6.0 | 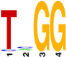 | 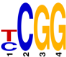 |
| Egr1 | 10 | 10.0 | 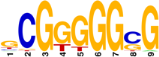 | 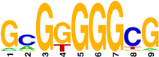 |
| Myb | 10 | 10.0 | 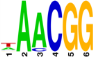 | 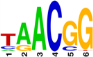 |
| ETS1 | 4.6 | 4.5 | 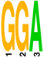 | 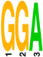 |
| Sfpi1 | 8.3 | 10.0 | 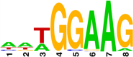 | 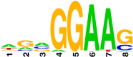 |
| Mafb | 4.4 | 10.0 | 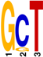 | 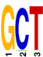 |

**Table S4. Performance of TF2DNA on test sets**

The set of 311 TFs with available experimentally determined PWMs from either JASPAR or UniPROBE databases were divided into 10% bins (except the last three that were pooled together into the “less than 30% set”, lt30set) according to the sequence identity between the TF sequences and best template found in the curated structural collection. The 3^rd^ column shows the distribution of cases among the 8 bins. The 4^th^ column shows the method performances, measured as the fraction of correctly predicted motifs (at 95% confidence level). The 5^th^ column displays the average percentages of TF interface conservation (TIC; defined as the identity between target and template residues that are at a distance of 4.5 Å from any DNA atom).

| **Test set name** | **%ID range** | **Number of cases** | **Performance** | **TFIC** |
| --- | --- | --- | --- | --- |
| 90set | 90 ≤ %ID ≤ 100 | 61 | 74.8 | 98.2 |
| 80set | 80 ≤ %ID < 90 | 26 | 77.8 | 98.2 |
| 70set | 70 ≤ %ID < 80 | 33 | 81.2 | 97.9 |
| 60set | 60 ≤ %ID < 70 | 48 | 78.0 | 87.8 |
| 50set | 50 ≤ %ID < 60 | 35 | 76.8 | 84.5 |
| 40set | 40 ≤ %ID < 50 | 40 | 81.8 | 79.3 |
| 30set | 30 ≤ %ID < 40 | 44 | 49.0 | 62.2 |
| lt30set | %ID < 30 | 24 | 45.2 | 32.1 |

**Table S5. Benchmark using the Rosetta DNA software to calculate TF-DNA binding energies**

We have randomly selected 80 TFs from the test set of 311 TFs with experimental binding motifs, 10 from each bin of 10% identity to the template TF sequence. The second column indicates the similarity Z-score between experimental and TF2DNA predicted motifs. The third column contains similarity Z-scores between experimental and Rosetta predicted motifs.

| **TF name** | **TF2DNA Z-score** | **Rosetta Z-score** | **%id to template** |
| --- | --- | --- | --- |
| Klf4 | 6.2 | -0.4 | 100.0 |
| eve | 5.7 | -0.3 | 100.0 |
| NR3C1 | 3.9 | -0.2 | 100.0 |
| GATA3 | 0.3 | 0.6 | 100.0 |
| Nkx2-5 | 9.3 | -0.1 | 98.2 |
| Egr1 | 10.0 | 1.6 | 93.4 |
| Myc | 4.8 | -0.4 | 92.6 |
| T | 9.2 | 0.0 | 91.8 |
| Antp | 4.9 | -0.3 | 90.9 |
| Gata5 | -0.4 | -0.5 | 90.5 |
| Evx2 | 3.9 | 0.2 | 89.5 |
| Evx1 | 5.0 | 0.0 | 89.5 |
| FOXO3 | 10.0 | -0.2 | 89.0 |
| Scr | 8.6 | -0.4 | 87.3 |
| Hoxb9 | 1.9 | -0.1 | 87.0 |
| Hoxd1 | 7.7 | -0.1 | 84.7 |
| MEF2A | 2.0 | -0.6 | 84.6 |
| Nkx2-4 | 0.0 | 0.1 | 82.5 |
| SuH | 9.8 | 0.0 | 81.5 |
| Hoxc5 | 10.0 | -0.2 | 80.0 |
| Hoxb4 | 8.2 | -0.4 | 78.2 |
| Hoxd8 | 10.0 | -0.2 | 78.2 |
| Hoxa10 | 5.6 | 0.9 | 77.8 |
| Alx4 | 7.8 | 0.3 | 77.6 |
| PHDP | 8.2 | 0.5 | 75.9 |
| ceh-22 | 0.8 | 0.1 | 71.4 |
| Esrrb | 10.0 | 0.2 | 71.4 |
| CG32532 | 3.6 | -0.4 | 71.2 |
| otp | 6.0 | -0.3 | 70.7 |
| Otp | 6.0 | -0.3 | 70.7 |
| Ptx1 | 4.5 | -0.2 | 69.0 |
| Prrx2 | 3.9 | -0.4 | 67.8 |
| Prrx1 | 4.3 | -0.4 | 67.8 |
| Dmbx1 | 5.8 | -0.3 | 67.2 |
| OdsH | 5.0 | -0.5 | 66.7 |
| Crx | 3.9 | -0.1 | 66.7 |
| HSF1 | 0.1 | -0.2 | 65.2 |
| NR2F1 | 6.3 | -0.1 | 64.9 |
| Cdx1 | 2.0 | -0.3 | 61.8 |
| bsh | 4.3 | -0.4 | 61.4 |
| Etv3 | 3.9 | -0.3 | 58.9 |
| NK7.1 | 9.7 | -0.2 | 58.6 |
| Vax2 | 4.1 | 0.1 | 57.9 |
| Meox1 | 4.0 | 0.6 | 57.9 |
| Hoxa11 | 1.0 | 0.3 | 56.1 |
| CG18599 | 2.4 | -0.4 | 55.9 |
| Barx2 | 5.7 | -0.5 | 54.4 |
| Hoxd12 | 0.1 | -0.3 | 52.6 |
| Barx1 | 3.9 | 0.2 | 52.6 |
| ems | 4.5 | -0.4 | 52.6 |
| Foxj3 | 10.0 | -0.2 | 48.3 |
| Lim1 | 6.5 | -0.2 | 47.4 |
| slbo | 3.2 | 1.3 | 45.5 |
| YAP1 | 4.5 | -0.3 | 44.6 |
| SFL1 | -0.3 | 0.0 | 44.3 |
| Awh | 4.1 | -0.1 | 43.9 |
| Rhox6 | 1.4 | -0.2 | 42.4 |
| HCM1 | 10.0 | -0.6 | 42.0 |
| Lmx1a | 9.1 | 0.2 | 40.4 |
| Duxl | 2.4 | -0.3 | 40.4 |
| HLF | 0.7 | 1.0 | 39.7 |
| Obox2 | 5.6 | -0.2 | 38.6 |
| YOX1 | 10.0 | 0.0 | 38.1 |
| ATHB-5 | 4.0 | -0.4 | 36.4 |
| CG11617 | 3.1 | -0.1 | 35.6 |
| Tgif2 | 0.2 | 0.1 | 34.9 |
| Tgif2 | 0.2 | 0.1 | 34.9 |
| Pknox1 | 0.1 | 0.5 | 33.3 |
| YAP6 | 0.4 | 0.1 | 31.2 |
| hb | 2.2 | 0.2 | 31.2 |
| Hmbox1 | 3.7 | 0.4 | 29.7 |
| gt | 0.8 | -0.2 | 29.6 |
| MGA1 | 0.1 | -0.5 | 29.2 |
| YPR196W | 1.9 | 1.9 | 28.9 |
| RTG3 | 10.0 | -0.1 | 28.4 |
| INO4 | 5.2 | 1.5 | 27.5 |
| INO2 | 8.2 | -0.2 | 27.4 |
| Hdx | 0.2 | -0.1 | 25.4 |
| br | -0.2 | 0.1 | 25.4 |
| NFATC2 | 2.9 | -0.4 | 21.9 |

**Table S 6. Template usage in the prediction of human TF binding sites**

The table shows the number of templates that are reused in modeling the set of human TF sequences (first column). The second column has the computed frequency of each “template reuse” bin. The third column contains the percentage of reused templates and the fourth column its cumulative distribution. Only 0.1% of the TF sequences use unique templates, whereas 53.1% of the sequences were modeled with one template.

| **Reused templates** | **Frequency** | **% reused templates** | **cumulative distribution** |
| --- | --- | --- | --- |
| 1 | 16 | 0.1 | 100.0 |
| 2 | 18 | 0.2 | 99.9 |
| 3 | 14 | 0.3 | 99.7 |
| 4 | 7 | 0.4 | 99.4 |
| 5 | 3 | 0.5 | 98.9 |
| 6 | 7 | 0.6 | 98.4 |
| 7 | 8 | 0.7 | 97.8 |
| 8 | 6 | 0.8 | 97.0 |
| 9 | 3 | 1.0 | 96.2 |
| 10 | 2 | 1.1 | 95.2 |
| 11 | 3 | 1.2 | 94.2 |
| 12 | 2 | 1.3 | 93.0 |
| 13 | 2 | 1.4 | 91.7 |
| 14 | 1 | 1.5 | 90.4 |
| 15 | 1 | 1.6 | 88.9 |
| 16 | 2 | 1.7 | 87.3 |
| 20 | 1 | 2.1 | 85.6 |
| 22 | 1 | 2.3 | 83.5 |
| 23 | 1 | 2.4 | 81.1 |
| 24 | 1 | 2.5 | 78.7 |
| 27 | 1 | 2.9 | 76.2 |
| 28 | 1 | 3.0 | 73.3 |
| 39 | 2 | 4.1 | 70.3 |
| 41 | 1 | 4.3 | 66.2 |
| 83 | 1 | 8.8 | 61.9 |
| 501 | 1 | 53.1 | 53.1 |

**Table S7. Multiplicity of human promoters**

The RefSeq database contains 23,340 protein-coding gene promoters (see Methods for precise definitions). These promoters are degenerate because they relate to 18,515 unique gene identifiers. This table presents the number of genes for each promoter multiplicity observed (ranging from 1 through 10). Only 18.1% of the genes have a multiplicity of 2 or greater.

| **Promoter multiplicity** | **Number of genes** | **Percentage of genes** | **Cumulative percentage** |
| --- | --- | --- | --- |
| 1 | 15,158 | 81.9 | 81.9 |
| 2 | 2,400 | 13.0 | 94.8 |
| 3 | 648 | 3.5 | 98.3 |
| 4 | 199 | 1.1 | 99.4 |
| 5 | 60 | 0.3 | 99.7 |
| 6 | 27 | 0.1 | 99.9 |
| 7 | 9 | 0.0 | 99.9 |
| 8 | 10 | 0.1 | 100.0 |
| 9 | 3 | 0.0 | 100.0 |
| 10 | 1 | 0.0 | 100.0 |
| total | 18,515 | 100.0 | - |

**Table S8. Clustering of human TFs by binding motif similarity**

The 1,321 human predicted TF binding motifs were compared and a similarity tree was constructed. A similarity Z-score cutoff of 2 was used to define clusters (95% confidence that two motifs should cluster together). This cutoff defines 294 TF clusters.

| **Cluster number** | **Number of elements** | **List of TFs in cluster** |
| --- | --- | --- |
| 1 | 66 | ALX1 ALX4 ARGFX ARX BARHL1 BARX1 BARX2 CDX1 CDX2 CDX4 DLX2 DLX3 DPRX DRGX ESX1 GSX1 GSX2 HESX1 HMX1 HOXA11 HOXA2 HOXA3 HOXA4 HOXA5 HOXA6 HOXB13 HOXB3 HOXB4 HOXB5 HOXB8 HOXC11 HOXC12 HOXC13 HOXC4 HOXC5 HOXC8 HOXD12 HOXD3 HOXD4 HOXD8 ISX LHX3 LMX1A LMX1B MEOX2 MNX1 PDX1 PITX1 PITX2 RAX UNCX HOXA7 HOXA9 HOXB2 KAT6B LHX6 LHX8 VSX2 ZFHX3 MIXL1 OTX1 PHOX2A PHOX2B PITX3 PROP1 SIX2 |
| 2 | 24 | ESR1 ESR2 ESRRA ESRRB ESRRG NR1H2 NR1H4 NR1I3 VDR NR1D1 NR1D2 NR4A1 PPARA PPARG RARA RORB RORC RARG NR5A2 PPARD RORA THRB NR1I2 NR2F6 |
| 3 | 20 | ADNP EGR1 EGR2 KLF1 KLF10 KLF11 KLF13 KLF15 KLF17 KLF2 KLF4 KLF7 KLF8 SP1 SP3 SP4 SP7 SP8 SP9 WT1 |
| 4 | 20 | ATF2 ATF7 BACH1 CREB1 CREB3L1 DBP NFIL3 ATF6 FOSL2 JUN JUND ATF4 ATF5 BATF2 CREB5 CREM CEBPB CEBPE JUNB XBP1 |
| 5 | 19 | FIZ1 HIC2 HINFP ZBTB10 ZBTB46 ZNF408 ZNF644 KLF14 MYF5 NEUROG1 NFAT5 NHLH2 OLIG2 TCF15 MYOD1 TCF12 TCF4 NEUROG3 ZBTB48 |
| 6 | 18 | BACH2 CREB3L4 DLX5 EMX1 HOXD1 MSX1 MSX2 NKX6-2 NKX6-3 BARHL2 BSX DLX1 GBX1 LHX4 EMX2 NANOG OTP VENTX |
| 7 | 17 | DLX4 GBX2 HHEX HOXA1 NKX2-3 NKX6-1 LBX1 LBX2 NKX2-6 NKX2-1 NKX2-2 NKX2-4 NKX2-5 NKX2-8 NKX3-1 NKX3-2 TLX1 |
| 8 | 17 | FOXA1 FOXA2 FOXA3 FOXB1 FOXB2 FOXC2 FOXD3 FOXD4 FOXD4L1 FOXD4L3 FOXE3 FOXI2 FOXL2 FOXQ1 FOXS1 FOXK1 FOXK2 |
| 9 | 16 | ZBTB49 ZBTB7C ZNF275 ZNF599 ZNF720 ZNF740 ZNF202 ZNF263 ZNF215 ZNF257 ZNF517 ZNF682 ZFY ZNF611 ZNF579 ZNF669 |
| 10 | 15 | PATZ1 ZBTB7A ZBTB7B ZEB2 ZNF543 ZNF580 ZNF454 ZNF799 ZSCAN25 ZFP57 ZNF148 ZNF75D ZBTB22 ZBTB8B ZNF273 |
| 11 | 15 | CHD1 DMBX1 SOX11 SOX13 SOX4 SOX5 SRY SOX12 SOX14 SOX15 SOX2 SOX8 SOX9 HOMEZ SOX21 |
| 12 | 14 | FOXI1 FOXJ2 FOXJ3 FOXM1 FOXN1 FOXN2 FOXN4 FOXO3 FOXO6 FOXO1 FOXP3 FOXP4 FOXP2 FOXR1 |
| 13 | 14 | HMX2 HMX3 NR5A1 ZFP37 ZNF197 ZNF37A ZNF473 ZNF484 HOXA13 ZNF107 ZBTB12 ZNF57 SSH1 ZNF446 |
| 14 | 14 | EOMES JRKL TBX21 MGA TBR1 TBX1 TBX10 TBX15 TBX20 TBX22 TBX18 TBX4 TBX5 TBX6 |
| 15 | 13 | HKR1 KLF12 KLF5 SP2 SP5 SP6 ZNF112 ZNF541 ZNF165 ZNF281 PRDM9 ZNF429 ZNF836 |
| 16 | 13 | HES1 MLXIP MXD1 MYC MYCL TCFL5 USF1 MYCN MAX USF2 MXI1 ZNF691 HES2 |
| 17 | 13 | GLI1 GLI2 GLI3 RBAK ZIC2 ZNF846 GLIS1 GLIS3 ZIC3 ZIC4 ZIC5 GLIS2 ZIC1 |
| 18 | 13 | IRF3 ZNF169 ZNF233 ZNF250 ZSCAN12 ZFP62 ZNF514 ZNF575 ZNF2 ZNF586 ZNF766 ZNF252P ZNF664 |
| 19 | 13 | DMTF1 E4F1 IKZF1 ZNF667 IFI16 ZNF132 ZNF274 ZNF420 ZNF500 ZNF791 ZNF200 ZNF678 ZNF8 |
| 20 | 12 | EHF ELF5 ELK3 ETV6 RBPJL SPDEF ELF4 ETS1 ETS2 ETV2 ELF1 ETV7 |
| 21 | 12 | ATF1 DBX2 EVX1 VAX2 ZNF780A ZKSCAN5 ZNF425 DNAJC21 EVX2 PBX3 POU6F2 KMT2C |
| 22 | 12 | ATF3 BATF BATF3 FOS FOSL1 HLF JDP2 NFE2L2 POU2F1 POU5F1a POU5F1b ZBTB41 |
| 23 | 12 | HNF4A HNF4G NR2C2 NR2E1 NR2F1 NR2F2 NR2C1 NR4A2 RXRA RXRG NR2E3 RXRB |
| 24 | 11 | ZFP2 ZFX ZNF444 ZNF71 ZNF730 ZSCAN9 ZKSCAN1 ZKSCAN3 ZNF627 ZNF783 ZSCAN10 |
| 25 | 11 | ARNT2 ARNTL2 BHLHE40 BHLHE41 HELT HEY2 MITF SREBF1 TFE3 TFEB TFEC |
| 26 | 11 | PRDM10 ZBTB24 ZNF407 ZNF141 ZNF254 ZNF676 ZNF788 ZNF785 ZNF426 ZNF527 ZNF701 |
| 27 | 10 | GSC IRX4 ISL2 OTX2 PAX6 IRX1 IRX2 IRX3 IRX5 IRX6 |
| 28 | 10 | POU1F1 POU3F1 POU3F2 POU3F3 POU3F4 POU4F2 ZNF665 POU4F3 ZNF16 ZNF232 |
| 29 | 10 | ZKSCAN2 ZNF383 ZNF695 ZNF835 ZNF239 ZNF776 ZNF891 ZNF354A ZNF551 ZNF570 |
| 30 | 10 | TP53 ZNF155 ZNF563 ZNF696 ZNF335 ZNF556 ZNF786 ZNF576 ZXDB ZXDC |
| 31 | 9 | HMBOX1 PAX3 PRRX2 NOTO SHOX SHOX2 PAX7 ZFHX2 PRRX1 |
| 32 | 9 | CENPB FOXC1 FOXD2 FOXE1 FOXF1 FOXF2 FOXG1 FOXJ1 FOXL1 |
| 33 | 8 | ZBTB43 ZNF48 ZNF304 ZNF396 ZNF227 ZNF654 ZNF324 ZNF382 |
| 34 | 8 | ZBTB6 ZNF506 ZNF573 ZNF366 ZNF771 ZNF780B ZNF714 ZNF90 |
| 35 | 8 | PRDM12 ZBTB25 ZNF22 ZNF816 ZNF430 ZSCAN20 ZNF358 ZNF416 |
| 36 | 8 | PEG3 TP73 ZNF554 REST ZNF532 ZSCAN22 ZBTB33 ZBTB38 |
| 37 | 8 | FARSA ZNF236 ZNF99 ZNF813 OVOL2 TGIF2 ZNF653 ZNF692 |
| 38 | 8 | IRF1 IRF2 IRF4 IRF6 IRF8 IRF9 ZNF765 IRF5 |
| 39 | 7 | MECOM PRDM16 ZBTB45 ZNF319 ZNF467 ZNF726 ZNF705A |
| 40 | 7 | ASCL2 TFAP4 ASCL4 ATOH8 HAND2 HES4 OLIG1 |
| 41 | 7 | ERF ETV3 ETV4 ERG FLI1 ETV1 FEV |
| 42 | 7 | CREB3 TPRX1 CRX HLX TLX2 LEUTX SRF |
| 43 | 7 | BAZ2B UBP1 HBP1 SOX18 SOX17 SOX7 RAD51 |
| 44 | 7 | DDIT3 DEPDC4 ZNF594 DNAJC2 MTA2 STAT3 ZNF687 |
| 45 | 7 | MYNN PRDM14 ZKSCAN8 ZNF235 ZNF211 ZNF513 ZNF729 |
| 46 | 6 | ZNF283 ZNF558 ZNF84 ZNF449 ZNF568 ZNF713 |
| 47 | 6 | KLF16 KLF6 KLF9 ZBTB18 ZNF468 ZNF546 |
| 48 | 6 | ATMIN ZNF493 ZNF662 TRAFD1 ZFP1 ZNF485 |
| 49 | 6 | MEF2A MEF2C MEF2D POU6F1 ZBTB40 ZNF749 |
| 50 | 6 | ZNF214 ZNF32 ZNF623 ZNF30 ZNF256 ZSCAN4 |
| 51 | 6 | ZNF143 ZNF75A ZNF410 ZNF443 ZNF709 ZNF823 |
| 52 | 6 | ZFP41 ZFP82 ZNF14 ZNF286A ZNF431 ZNF628 |
| 53 | 6 | ZNF182 ZNF419 ZNF684 ZNF559 ZNF652 ZSCAN23 |
| 54 | 6 | PRDM6 ZBTB20 ZNF91 ZNF24 ZNF322P1 ZNF793 |
| 55 | 6 | CEBPA CEBPG ZNF519 ZNF222 ZNF223 ZNF230 |
| 56 | 6 | MXD4 ZNF471 ZNF121 ZNF284 ZNF552 ZNF697 |
| 57 | 6 | PBX1 PBX2 PBX4 PKNOX1 PKNOX2 ZNF280B |
| 58 | 5 | ZNF133 ZNF774 ZSCAN32 ZNF479 ZNF792 |
| 59 | 5 | IKZF4 ZBTB8A ZBTB9 ZEB1 ZNF445 |
| 60 | 5 | ZNF101 ZNF648 ZNF808 ZNF418 ZNF746 |
| 61 | 5 | ZNF229 ZNF582 ZNF778 ZNF33B ZNF574 |
| 62 | 5 | ELK4 GABPA RELB NFATC2 ZSCAN5C |
| 63 | 5 | MSGN1 NEUROD6 TIGD3 NFIB SNAI3 |
| 64 | 5 | BHLHA15 FIGLA NCOA3 MESP1 MNT |
| 65 | 5 | ARNTL MLXIPL SREBF2 ZNF277 ZNF747 |
| 66 | 5 | NFATC1 SMAD2 NHLH1 ZNF529 ZNF346 |
| 67 | 5 | HSF1 HSFY1 HSFY2 HSF2 HSF5 |
| 68 | 5 | HOXB9 HOXC9 HOXD10 HOXD9 HOXD13 |
| 69 | 5 | ALX3 DLX6 LHX2 HOXC10 TSHZ1 |
| 70 | 5 | SATB1 SATB2 TFDP1 TFDP2 TGIF2LY |
| 71 | 5 | GRHL2 ZNF763 ZNF540 ZNF35 ZNF354B |
| 72 | 5 | CERS4 ZBP1 NFIX ZNF267 ZNF777 |
| 73 | 5 | BNC1 MBD2 ZMAT3 TFAP2A TFAP2C |
| 74 | 5 | MEIS1 MEIS3 TRMT1L ZNF512B SSH2 |
| 75 | 5 | ID2 MAZ ZNF45 ZNF716 NUFIP1 |
| 76 | 5 | POU4F1 STAT1 ZNF528 ZNF154 ZSCAN21 |
| 77 | 5 | ZIK1 ZNF177 ZNF852 ZNF287 ZNF3 |
| 78 | 5 | GZF1 ZNF157 ZNF775 ZNF74 ZNF781 |
| 79 | 5 | DMRT2 E2F8 ZNF518B ZNF597 ZNF789 |
| 80 | 5 | CDC5L VEZF1 ZNF34 ZNF433 ZNF41 |
| 81 | 5 | SCRT1 ZNF251 ZNF585A ZNF81 ZNF596 |
| 82 | 4 | BCL11A BCL11B ZNF296 ZNF347 |
| 83 | 4 | ZNF442 ZNF534 ZNF672 ZNF805 |
| 84 | 4 | ZNF140 ZNF502 ZNF562 ZNF646 |
| 85 | 4 | TERF1 ZNF334 ZNF19 ZNF668 |
| 86 | 4 | NFE2 NFE2L1 PRDM4 ZNF124 |
| 87 | 4 | ATOH1 ATOH7 NEUROG2 BHLHA9 |
| 88 | 4 | BHLHE22 MXD3 MYF6 NEUROD1 |
| 89 | 4 | OLIG3 TCF21 TCF3 ZNF207 |
| 90 | 4 | ASCL3 NEUROD4 CEBPZ SMAD6 |
| 91 | 4 | HOXC6 NOBOX RAX2 ZNF620 |
| 92 | 4 | HOXB1 ISL1 LHX5 MEOX1 |
| 93 | 4 | ZNF135 ZNF621 ZNF146 ZNF583 |
| 94 | 4 | CREB3L3 CREBRF TFAM ZFP14 |
| 95 | 4 | GATA2 GATA3 STAT2 ZNF521 |
| 96 | 4 | RERE TAX1BP1 SOX3 ZNF208 |
| 97 | 4 | LHX1 TFAP2E ZNF438 ZNF76 |
| 98 | 4 | DNAJC1 MKX TGIF2LX ZFP64 |
| 99 | 4 | HMGXB4 STAT6 HOXB7 MTA3 |
| 100 | 4 | AR NR3C2 PGR NR3C1 |
| 101 | 4 | STAT4 ZNF181 ZNF221 ZNF516 |
| 102 | 4 | CERS5 POU2F2 ZNF544 ZNF639 |
| 103 | 4 | FEZF2 GFI1 GFI1B ZNF92 |
| 104 | 4 | PRDM1 ZNF317 SCRT2 ZNF681 |
| 105 | 4 | FOXP1 HSF4 TRMT1 ZCCHC11 |
| 106 | 4 | SALL1 SALL4 ZNF212 ZNF33A |
| 107 | 4 | ZNF329 ZNF391 ZNF79 ZNF555 |
| 108 | 4 | MYBL2 ZNF77 ZBTB16 ZBTB32 |
| 109 | 4 | OSR1 ZBTB47 TP63 ZNF708 |
| 110 | 4 | ZBTB4 ZNF300 ZNF530 ZNF655 |
| 111 | 4 | ZFP69B ZNF569 ZNF860 ZNF83 |
| 112 | 4 | ZNF18 ZNF354C ZNF572 ZNF671 |
| 113 | 4 | ZNF174 ZNF790 ZNF461 ZNF649 |
| 114 | 4 | MAFA MAFB MAFF NRL |
| 115 | 4 | ZBTB26 ZNF547 ZNF436 ZNF782 |
| 116 | 4 | MYB PAX9 ZNF142 ZUFSP |
| 117 | 4 | IKZF2 PAX2 TGIF1 MIER3 |
| 118 | 4 | NFE2L3 ONECUT2 ONECUT1 ONECUT3 |
| 119 | 4 | GATA4 RC3H2 ZNF658 ZSCAN2 |
| 120 | 3 | PAX4 ZNF440 ZSCAN5B |
| 121 | 3 | PLAG1 PLAGL2 ZNF423 |
| 122 | 3 | AEBP2 INSM2 KLF3 |
| 123 | 3 | ZNF100 ZNF320 ZNF561 |
| 124 | 3 | EGR3 EGR4 ZNF134 |
| 125 | 3 | OSR2 ZNF732 ZNF189 |
| 126 | 3 | ZNF12 ZNF571 ZNF607 |
| 127 | 3 | ZNF497 ZNF589 ZNF764 |
| 128 | 3 | ZNF23 ZNF510 ZNF837 |
| 129 | 3 | E2F7 FOSB ZNF341 |
| 130 | 3 | SNAI2 ZNF404 ZNF675 |
| 131 | 3 | TFAP2D ZNF606 ZNF93 |
| 132 | 3 | RCOR2 TERF2 ZNF577 |
| 133 | 3 | MEIS2 ZNF496 ZNF415 |
| 134 | 3 | ZNF397 ZNF44 ZNF483 |
| 135 | 3 | FERD3L KIAA2018 MSC |
| 136 | 3 | SCXA SOX10 TWIST1 |
| 137 | 3 | ASCL1 HES5 HES7 |
| 138 | 3 | CLOCK HEY1 NPAS2 |
| 139 | 3 | LYL1 TAL1 TAL2 |
| 140 | 3 | BHLHE23 ZBTB39 ZNF581 |
| 141 | 3 | NFKB1 NFKB2 ZNF592 |
| 142 | 3 | NFATC4 ZCCHC6 RELA |
| 143 | 3 | IKZF5 TSHZ3 ZNF501 |
| 144 | 3 | ZBTB21 ZMAT4 ZNF622 |
| 145 | 3 | RNF138 SP100 ZNF160 |
| 146 | 3 | HOPX HOXD11 SIX4 |
| 147 | 3 | DBX1 LHX9 TLX3 |
| 148 | 3 | RHOXF1 SIX1 SIX5 |
| 149 | 3 | HOXB6 TFCP2 TFCP2L1 |
| 150 | 3 | EDF1 ZSCAN18 ZNF20 |
| 151 | 3 | CREB3L2 HDX VAX1 |
| 152 | 3 | EN1 EN2 RHOXF2 |
| 153 | 3 | CASZ1 LEF1 ZNF474 |
| 154 | 3 | FEZF1 GATA5 ZNF384 |
| 155 | 3 | CTCFL RAG1 RLF |
| 156 | 3 | GATA1 MTA1 ZNF266 |
| 157 | 3 | ZFP28 ZNF131 ZNF333 |
| 158 | 3 | ZNF28 ZNF549 ZNF70 |
| 159 | 3 | NCOR2 NRK ZFP91 |
| 160 | 3 | CHD2 OVOL3 ZNF536 |
| 161 | 3 | HIVEP1 HIVEP3 NFIC |
| 162 | 3 | ZNF689 ZNF772 ZNF784 |
| 163 | 3 | ZNF679 ZNF85 ZSCAN16 |
| 164 | 3 | REPIN1 ZFAT ZNF683 |
| 165 | 3 | ID1 ZNF195 ZNF724P |
| 166 | 3 | ZNF311 ZNF560 ZNF615 |
| 167 | 3 | ZNF660 ZNF677 ZNF721 |
| 168 | 3 | BCL6B ZNF700 ZNF184 |
| 169 | 3 | ZNF26 ZNF268 ZNF626 |
| 170 | 3 | ZNF180 ZNF394 ZNF488 |
| 171 | 3 | RREB1 ZNF710 ZSCAN30 |
| 172 | 3 | ZMAT1 ZNF557 ZNF680 |
| 173 | 3 | CIZ1 MAFG ZFP3 |
| 174 | 3 | MAFK SMAD1 REL |
| 175 | 3 | MZF1 PAX5 ZNF821 |
| 176 | 3 | ZBTB14 ZNF849P ZNF292 |
| 177 | 3 | IRF7 ZBTB5 ZNF480 |
| 178 | 3 | NR1H3 NR4A3 NR6A1 |
| 179 | 3 | TEF ZSCAN5A THRA |
| 180 | 3 | RBPJ TBX19 ZNF566 |
| 181 | 3 | TBX2 TBX3 ZBTB2 |
| 182 | 3 | SPI1 SPIB SPIC |
| 183 | 3 | INSM1 ZNF451 ZNF441 |
| 184 | 2 | OVOL1 ZIM3 |
| 185 | 2 | ZBTB1 ZNF610 |
| 186 | 2 | ELK1 PLAGL1 |
| 187 | 2 | ZKSCAN4 ZNF460 |
| 188 | 2 | ZNF490 ZNF616 |
| 189 | 2 | ID4 MKRN3 |
| 190 | 2 | ZNF491 ZNF80 |
| 191 | 2 | ZBTB37 ZNF337 |
| 192 | 2 | RNF114 ZNF17 |
| 193 | 2 | PTF1A ZNF773 |
| 194 | 2 | ZNF331 ZNF470 |
| 195 | 2 | POGK ZNF264 |
| 196 | 2 | ELF3 PRDM15 |
| 197 | 2 | ZNF217 ZNF629 |
| 198 | 2 | ZNF117 ZNF43 |
| 199 | 2 | ARNT RFX5 |
| 200 | 2 | TCF23 ZNF280D |
| 201 | 2 | HES6 MLX |
| 202 | 2 | ZNF282 ZNF398 |
| 203 | 2 | DZIP1L HEYL |
| 204 | 2 | PRDM2 ZNF114 |
| 205 | 2 | SP140 ZNF302 |
| 206 | 2 | HIC1 SMARCC2 |
| 207 | 2 | ADNP2 ZNF787 |
| 208 | 2 | ZFP42 ZNF226 |
| 209 | 2 | NEUROD2 YY1 |
| 210 | 2 | ZBTB11 ZNF841 |
| 211 | 2 | ZNF800 ZNF827 |
| 212 | 2 | MYOG PRDM8 |
| 213 | 2 | BCL6 HIVEP2 |
| 214 | 2 | HSFX2 ZNF414 |
| 215 | 2 | RNF166 ZNF280A |
| 216 | 2 | MTF1 POGZ |
| 217 | 2 | CUX2 ZFHX4 |
| 218 | 2 | GRHL3 ZNF385B |
| 219 | 2 | ZBTB17 ZFP69 |
| 220 | 2 | JAZF1 ZNF638 |
| 221 | 2 | DEPDC1B ZNF831 |
| 222 | 2 | CREBL2 RNF125 |
| 223 | 2 | RC3H1 SOX1 |
| 224 | 2 | CERS2 ZNF219 |
| 225 | 2 | CREBZF FOXN3 |
| 226 | 2 | TIGD4 ZFPM2 |
| 227 | 2 | ZFP30 ZNF670 |
| 228 | 2 | ZNF138 ZNF248 |
| 229 | 2 | BNC2 CCDC79 |
| 230 | 2 | MYBL1 ZKSCAN7 |
| 231 | 2 | SNAPC4 ZNF7 |
| 232 | 2 | ZNF362 ZNF613 |
| 233 | 2 | ZNF25 ZNF565 |
| 234 | 2 | ZNF205 ZNF343 |
| 235 | 2 | SNAI1 ZNF439 |
| 236 | 2 | ZNF213 ZNF567 |
| 237 | 2 | PRDM5 SALL2 |
| 238 | 2 | ZNF385A ZNF526 |
| 239 | 2 | SMARCA1 ZNF367 |
| 240 | 2 | ZNF598 ZNF699 |
| 241 | 2 | ZNF624 ZSCAN29 |
| 242 | 2 | ZNF584 ZNF98 |
| 243 | 2 | CTCF MAF |
| 244 | 2 | HSFX1 ZNF614 |
| 245 | 2 | ZNF69 ZNF711 |
| 246 | 2 | ZNF385D ZNF587 |
| 247 | 2 | DHX34 SMAD5 |
| 248 | 2 | IKZF3 ZNF814 |
| 249 | 2 | SMAD4 SMAD9 |
| 250 | 2 | ZNF175 ZNF829 |
| 251 | 2 | GTF3A ZNF619 |
| 252 | 2 | PAX1 PRDM13 |
| 253 | 2 | SMARCA5 ZNF770 |
| 254 | 2 | CHAMP1 GATA6 |
| 255 | 2 | ZNF276 ZSCAN1 |
| 256 | 2 | FOXR2 T |
| 257 | 2 | TRPS1 ZFP90 |
| 258 | 2 | ZNF385C ZNF641 |
| 259 | 2 | GRHL1 SMAD7 |
| 260 | 2 | WIZ ZHX3 |
| 261 | 1 | ZNF605 |
| 262 | 1 | ELF2 |
| 263 | 1 | ZBTB44 |
| 264 | 1 | ZNF600 |
| 265 | 1 | EIF3K |
| 266 | 1 | HAND1 |
| 267 | 1 | POU2F3 |
| 268 | 1 | NFIA |
| 269 | 1 | ZNF524 |
| 270 | 1 | NFATC3 |
| 271 | 1 | VSX1 |
| 272 | 1 | PAX8 |
| 273 | 1 | ZNF843 |
| 274 | 1 | FOXH1 |
| 275 | 1 | FOXO4 |
| 276 | 1 | GSC2 |
| 277 | 1 | ZBTB3 |
| 278 | 1 | ZNF548 |
| 279 | 1 | RCOR3 |
| 280 | 1 | STAT5A |
| 281 | 1 | ZNF768 |
| 282 | 1 | ZNF280C |
| 283 | 1 | ZNF511 |
| 284 | 1 | ZNF507 |
| 285 | 1 | ZNF618 |
| 286 | 1 | ZFPM1 |
| 287 | 1 | SIX6 |
| 288 | 1 | ZNF136 |
| 289 | 1 | ZNF707 |
| 290 | 1 | RARB |
| 291 | 1 | TFAP2B |
| 292 | 1 | ZNF462 |
| 293 | 1 | GPN1 |
| 294 | 1 | ZBTB34 |

**Table S9. TLX3 expression profiles in diverse tissues from the Human Protein Atlas**

Protein expression of TLX3 in different tissues and cell types as assayed with tissue antibody staining. Expression levels are reported in four bins: strong (red), moderate (orange), weak (light orange) and negative (white).

| **Tissue** | **Cells** | **S** |  | **Tissue** | **Cells** | **S** |
| --- | --- | --- | --- | --- | --- | --- |
| **Nervous system cells** | |  |  | **Squamous epithelial cells** | |  |
| Soft tissue 1 | Peripheral nerve |  |  | Skin | Keratinocytes | 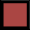 |
| Soft tissue 2 | Peripheral nerve | 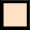 |  | Vulva/anal skin | Epidermal | 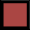 |
| Cerebral cortex | Neuronal | 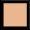 |  | Vagina | Squamous epithelial | 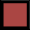 |
| Hippocampus | Neuronal | 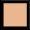 |  | Cervix, uterine | Squamous epithelial | 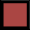 |
| Lateral ventricle | Neuronal | 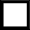 |  | Oral mucosa | Squamous epithelial | 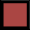 |
| Cerebellum | Purkinje | 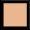 |  | Tonsil | Squamous epithelial | 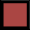 |
| Cerebellum | Cells in granular layer | 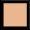 |  | Esophagus | Squamous epithelial | 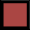 |
| Cerebellum | Cells in molecular layer | 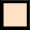 |  | **Endocrine cells** | |  |
| Cerebral cortex | Glial | 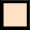 |  | Pancreas | Islets of Langerhans | 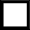 |
| Colon | Peripheral nerve/ganglion | 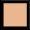 |  | Thyroid gland | Glandular | 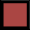 |
| Hippocampus | Glial | 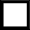 |  | Parathyroid gland | Glandular | 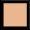 |
| Lateral ventricle | Glial | 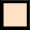 |  | Adrenal gland | Glandular | 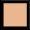 |
| Cerebral cortex | Neuropil | 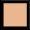 |  | Testis | Leydig | 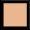 |
| **Hematopoetic cells** | |  |  | **Mesenchymal cells** | |  |
| Bone marrow | Hematopoietic | 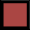 |  | Soft tissue 1 | Adipocytes | 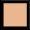 |
| Skin | Langerhans | 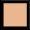 |  | Soft tissue 1 | Chondrocytes |  |
| Lymph node | Germinal center | 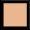 |  | Soft tissue 1 | Fibroblasts | 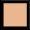 |
| Tonsil | Germinal center | 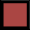 |  | Soft tissue 2 | Adipocytes | 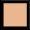 |
| Lymph node | Non-germinal center | 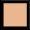 |  | Soft tissue 2 | Chondrocytes |  |
| Tonsil | Non-germinal center | 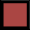 |  | Soft tissue 2 | Fibroblasts | 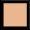 |
| Spleen | Cells in white pulp | 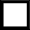 |  | Skin | Fibroblasts | 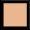 |
| Spleen | Cells in red pulp | 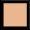 |  | Skeletal muscle | Myocytes | 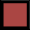 |
| Appendix | Lymphoid tissue | 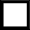 |  | Heart muscle | Myocytes | 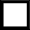 |
| Lung | Macrophages | 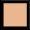 |  | Smooth muscle | Smooth muscle | 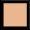 |
| **Glandular and transitional epithelial cells** | |  |  | Uterus, pre-menopause | Cells in endometrial stroma | 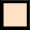 |
| Salivary gland | Glandular | 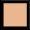 |  | Uterus, post-menopause | Cells in endometrial stroma | 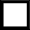 |
| Stomach, upper | Glandular | 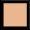 |  | Ovary | Ovarian stroma | 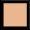 |
| Stomach, lower | Glandular | 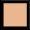 |  | Breast | Adipocytes | 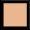 |
| Duodenum | Glandular | 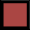 |  | **Other cells** | |  |
| Small intestine | Glandular | 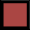 |  | Liver | Hepatocytes | 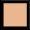 |
| Appendix | Glandular | 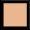 |  | Skin | Melanocytes | 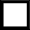 |
| Colon | Glandular | 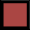 |  | Kidney | Cells in glomeruli | 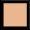 |
| Rectum | Glandular | 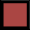 |  | Kidney | Cells in tubules | 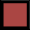 |
| Liver | Bile duct | 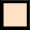 |  | Lung | Pneumocytes | 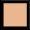 |
| Gallbladder | Glandular | 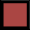 |  | Testis | Cells in seminiferus ducts | 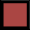 |
| Pancreas | Exocrine glandular | 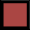 |  | Ovary | Follicle | 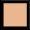 |
| Breast | Glandular | 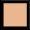 |  | Cerebral cortex | Endothelial | 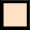 |
| Cervix, uterine | Glandular | 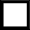 |  | Colon | Endothelial | 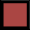 |
| Uterus, pre-menopause | Glandular | 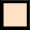 |  | Placenta | Decidual | 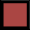 |
| Uterus, post-menopause | Glandular | 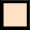 |  | Placenta | Trophoblastic | 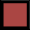 |
| Fallopian tube | Glandular | 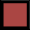 |  | Breast | Myoepithelial |  |
| Epididymis | Glandular |  |  |  |  |  |
| Prostate | Glandular |  |  |  |  |  |
| Seminal vesicle | Glandular |  |  | Level of expression: | Strong |  |
| Bronchus | Respiratory epithelial |  |  |  | Moderate |  |
| Nasopharynx | Respiratory epithelial |  |  |  | Weak |  |
| Urinary bladder | Urothelial |  |  |  | Negative |  |

**Table S10. Predicted TLX3 target genes**

List of 2,173 target genes of TLX3 using the TF2DNA predicted binding motif and the Motif Alignment and Search Tool (MAST) on all promoter sequences of RefSeq protein coding genes (23,340 total promoter regions from 18,515 unique genes). The list is ranked by their single best binding site score. The first 1,000 genes, used for the functional enrichment analysis are shown in bold. The genes RUNX1 (rank 535), VIP (rank 39) and SSTR2 (rank 187) are highlighted in green.

| **HGNC symbol** | | | | | |
| --- | --- | --- | --- | --- | --- |
| **HLA-DMA** | **DPY19L1** | **ANKDD1B** | NRF1 | ARHGEF3 | DNAJC12 |
| **DGKZ** | **TAGAP** | **SPACA5** | COL4A2 | COL24A1 | OR4K5 |
| **SGK3** | **KRTAP26-1** | **RNLS** | PRDM13 | GRXCR1 | PTCD3 |
| **CEP85L** | **ZNF799** | **OVOL2** | PRR19 | ZMIZ2 | SDC1 |
| **PFKM** | **USP39** | **ZKSCAN4** | EFCAB5 | ATXN7 | CDC23 |
| **TNFAIP6** | **CCR6** | **H2BFWT** | TMED10 | LRRC25 | IFNG |
| **TTC7A** | **C7** | **SP140** | NELL2 | CSTF2 | APBB2 |
| **C9orf66** | **RELL1** | **SMIM19** | BFAR | ERBB4 | UBE2D4 |
| **TGIF1** | **NLRP4** | **TMPRSS11F** | MICALL1 | BCS1L | SLC38A9 |
| **ACSM2A** | **CPSF1** | **TOR2A** | DHX57 | OR14A16 | TAF5L |
| **ACR** | **DIRAS2** | **PHTF2** | NLGN3 | ARMCX2 | LOC101928436 |
| **FBXO11** | **IQCF3** | **PLK1S1** | CEP128 | COPA | LYRM2 |
| **TYR** | **WDR72** | **ITPR2** | GTDC1 | KCNC2 | GAPT |
| **SMAP2** | **GABRR2** | **RGPD5** | ANKRD50 | PPP1R32 | KCTD8 |
| **CCNG1** | **MRPL34** | **ALDH3B1** | DGKI | SIAH2 | GGPS1 |
| **UCP1** | **FLJ45513** | **SULT1E1** | IGSF23 | CTDNEP1 | ZNF596 |
| **ERI2** | **PXK** | **MOG** | KIAA1377 | MAP2K5 | CA12 |
| **ADM** | **CRYGD** | **MORC4** | CELF1 | SGTA | SPSB2 |
| **SPATA25** | **IKZF1** | **NMB** | PPP1R42 | OR2M4 | VWA3B |
| **ARG1** | **OR6N2** | **LMOD2** | TMEM123 | CECR1 | PRMT6 |
| **OR4N4** | **DIMT1** | **METAP2** | NCOA2 | BLOC1S4 | CEP112 |
| **USP49** | **ELL** | **PRPSAP2** | RGS18 | ADORA3 | AGMAT |
| **ELAVL4** | **ACTN2** | **RDH11** | FABP7 | TCEAL4 | RFXANK |
| **OR51I1** | **NOX1** | **SMR3B** | WFDC11 | FAM89B | CNGA1 |
| **NOSIP** | **HIST1H4G** | **SLC2A6** | OR51A4 | MNAT1 | ZNRF1 |
| **RAD21** | **STC1** | **LGALS9B** | RIT2 | OLFML3 | RSPH10B2 |
| **SYT2** | **TMPRSS2** | **LRRD1** | NCOA7 | IFLTD1 | ZNF124 |
| **LPAR6** | **BTN2A2** | **ERH** | CYLC1 | RANBP2 | ZBTB45 |
| **GLIS1** | **TNPO1** | **ERAP2** | C2CD4D | POU2F1 | PRKX |
| **SLC29A1** | **EVI5L** | **MAP3K2** | SCN2A | SFR1 | RPS24 |
| **CBY3** | **TTYH2** | **SPDYA** | RTN4 | CRADD | C3orf38 |
| **ZBTB20** | **SNCG** | **PJA1** | TBL1X | RNF39 | TMEM66 |
| **HDAC9** | **NDUFA8** | **GNG11** | NUCB1 | NHLH2 | TMED5 |
| **FAM91A1** | **ADAM2** | **FAM229A** | OR4F4 | QSER1 | SMAD3 |
| **ZNF668** | **OTUD5** | **ZFYVE1** | KRT84 | SMG6 | POM121 |
| **ABCB5** | **PDYN** | **TNF** | RAB18 | TRIM15 | PCDHA12 |
| **ANTXR1** | **BCAT1** | **MC2R** | CCDC104 | SCGB3A2 | NRG1 |
| **GRAMD3** | **MYH1** | **KLHDC10** | LURAP1 | CBX2 | MPZ |
| **VIP** | **BANK1** | **SHROOM3** | BPY2B | ZXDB | DPYD |
| **TMEM25** | **UPB1** | **CYP2C9** | KLRF2 | NADSYN1 | COX5A |
| **OR8K5** | **PCDHGC3** | **DXO** | TAF8 | RGMA | SH3BP5 |
| **COL5A3** | **FAR2** | **TNIP3** | EHBP1 | RCHY1 | PCDHB7 |
| **CCDC85C** | **TRPC5OS** | **KLF14** | TRMT6 | GNGT1 | WBP2 |
| **LYRM7** | **ENPP7** | **IFNB1** | NPR1 | MED6 | COMT |
| **ALDH1A1** | **SHOC2** | **ARHGAP19** | B4GALT7 | PRAMEF8 | CAPZA2 |
| **ZKSCAN7** | **GNL1** | **HTR3E** | OR4C15 | GALNT14 | DDR2 |
| **LSM12** | **YARS2** | **FRMPD4** | MAZ | LIPE | HIPK1 |
| **RIMS1** | **ARHGAP6** | **EML4** | IL22RA2 | CCDC171 | CYGB |
| **SRPRB** | **MB** | **CCL11** | C11orf52 | CYP2C19 | FILIP1L |
| **ELMO1** | **LOC730159** | **CYP3A4** | CDH19 | TBC1D5 | METTL25 |
| **SLC12A1** | **CYP2C18** | **SCLT1** | C10orf25 | BET1 | USP40 |
| **FAM3A** | **NLGN1** | **FRG2B** | PDCD1LG2 | FABP2 | TBC1D8B |
| **LRRTM3** | **TP53I11** | **NPIPA7** | NXPE4 | DBX2 | DCDC1 |
| **TARP** | **TTC39C** | **HIVEP1** | OR10A7 | ZNF227 | MFSD11 |
| **SMC2** | **ZNF224** | **KATNAL1** | KLF5 | CCL2 | WASF2 |
| **ATOH7** | **HTR3C** | **SPARCL1** | GCHFR | OR4F29 | IL1RN |
| **NUP93** | **LMTK2** | **IFI27L2** | LIPI | GRM4 | SNTG1 |
| **NME5** | **C1orf94** | **ZAR1L** | C5orf22 | MCMDC2 | CCDC181 |
| **PRKACB** | **AMER2** | **METTL7A** | CDH17 | MST4 | FAM172A |
| **CTLA4** | **OR1A1** | **C5orf58** | NDUFB4 | TAS1R2 | PAGE2B |
| **ACE2** | **NPTX2** | **OR51L1** | SUMO4 | TP63 | MRGPRF |
| **MIA3** | **ZC3H14** | **SLC14A2** | ASH1L | FGD2 | MRPL15 |
| **KRTAP8-1** | **CCDC178** | **IRAK2** | DUSP19 | ARNT | RBM44 |
| **ZDBF2** | **NDE1** | **KEL** | PTDSS1 | OR51G2 | PCSK1 |
| **PDK3** | **DNAJB7** | **CARD16** | CLUAP1 | CLGN | PCGF5 |
| **PRL** | **DOCK8** | **SCN1A** | NOTO | ACSM3 | SDC3 |
| **OR6Y1** | **GJA8** | **RANBP17** | PRRC1 | TENM2 | HABP2 |
| **FCHO1** | **C22orf46** | **HBD** | HSD17B1 | TLR2 | VTN |
| **TTC18** | **TTC26** | **ZBED2** | ANO10 | CFH | DEFB115 |
| **ZNF219** | **PDX1** | **STOML2** | CXADR | TAAR9 | DNAJB13 |
| **ITGB1BP1** | **TP53TG5** | **TBC1D3C** | ATP5B | KIF2C | TIAF1 |
| **TRIM38** | **SLU7** | **B3GALT1** | BRIX1 | USP17L3 | SLC6A7 |
| **ZNF594** | **MMP13** | **OR51B4** | PTPRS | GTPBP8 | ARMC1 |
| **PEX14** | **LARP4** | **PKIA** | GADD45A | ASCL5 | RPS6 |
| **AIM2** | **IFIH1** | **TNFSF11** | HINFP | TMEM17 | TMEM54 |
| **FAM47A** | **PRR23D1** | **PAPOLG** | SRSF11 | ZPBP2 | COL10A1 |
| **GABRB1** | **FAT3** | **DEFB131** | RANBP3L | LOC440243 | C11orf30 |
| **ZFHX3** | **THNSL1** | **CSAD** | TGOLN2 | PHACTR3 | SMARCA2 |
| **WBP11** | **HBQ1** | **AMELY** | PLG | TRIM48 | ALDH18A1 |
| **EXOC1** | **SPG20** | **BMPR1B** | NTRK2 | TRIM61 | ACBD6 |
| **ANXA11** | **C3orf43** | **SPANXN3** | CYP11B2 | PPP1R12B | FAM26D |
| **PCDHA10** | **TREML4** | **ZNF248** | PTGER3 | FOXD4L4 | ZMYM3 |
| **MRGPRX3** | **POLI** | **CUL1** | KRTAP27-1 | OR4C16 | PRDM8 |
| **CCL20** | **PEX2** | **DIDO1** | RGS12 | RMDN1 | TRAPPC3 |
| **HMGCR** | **TAAR6** | **LACRT** | OR4N2 | CD274 | PCED1B |
| **MYF5** | **NLGN4Y** | **SLITRK2** | SCNN1A | SCRN3 | HAND1 |
| **ZFAND6** | **MAML2** | **OR4C11** | MROH2B | DSEL | CLRN3 |
| **LOC388780** | **AASDHPPT** | **OR4S2** | SPA17 | GALNT7 | SHCBP1L |
| **ARNTL2** | **BDH1** | **GPM6A** | ATP1B3 | FER1L6 | ERCC6L |
| **RIPPLY1** | **TCF4** | **FPR1** | CCDC15 | TTN | NARS2 |
| **EIF4A1** | **RAPGEF2** | **OR6C76** | TM4SF5 | GLRA2 | RASGEF1B |
| **PANK1** | **LYZL1** | **PDE1A** | PTMS | TRIQK | ADIPOQ |
| **ABCB1** | **EIF3C** | **KLHL7** | YKT6 | APPL2 | MAP7D1 |
| **TGM3** | **CTAGE15** | **SLAIN1** | CNIH4 | ZNF474 | NRDE2 |
| **NR3C1** | **OR2S2** | **AMACR** | PLGLB2 | HSPA13 | STK4 |
| **VSIG1** | **EXPH5** | **MAMLD1** | RRAGD | NCKAP1 | BCORL1 |
| **ARHGEF38** | **CDK12** | **HSD11B1** | EXOC8 | AKR1C1 | ARHGAP24 |
| **SELE** | **CHL1** | **ZNF528** | SNRNP35 | C5orf63 | ZSCAN21 |
| **BCHE** | **CSMD3** | **MIER1** | C9orf3 | CD3E | TBR1 |
| **C1orf137** | **SERPINB5** | **COL6A5** | NDUFAF4 | UFSP2 | IMPG2 |
| **PLIN1** | **1-Mar** | **MAP3K7CL** | DCAF8L1 | GABRE | CLEC5A |
| **C22orf31** | **CCNY** | **ZNF185** | NIP7 | FRG1 | CDC73 |
| **ANO7** | **CTAGE4** | **FRG2** | FER1L5 | ATP2C1 | UHRF1 |
| **PAMR1** | **RNPEP** | **GALNT12** | HIST1H2AE | NEK2 | OPA1 |
| **GPR113** | **CLEC12A** | **DNAJC13** | BRDT | SYNJ2 | PPIH |
| **GRIK5** | **MAGEL2** | **PPP2R5A** | RGS3 | PLCL1 | SERPINA5 |
| **LCK** | **DDX60L** | **PSCA** | INPP4A | OR8J1 | STAU2 |
| **USP6NL** | **C1D** | **HAPLN2** | CNTN3 | AKR1C4 | C9orf24 |
| **ZC3HC1** | **CCT5** | **TTC32** | RHOBTB2 | UBA5 | ACACA |
| **SYNPO** | **CMC1** | **CCDC149** | SLC24A1 | TATDN3 | RRM2B |
| **RMDN2** | **TRPM1** | **TMEM179** | MYL10 | BLOC1S6 | FAM198B |
| **MED12** | **DOC2A** | **SMARCC1** | THAP2 | PEAR1 | CSNK1A1L |
| **ZNF223** | **INTS6** | **OR9G1** | LDHD | RICTOR | TEAD2 |
| **CYP2C8** | **TRANK1** | **ESRRG** | EIF2A | OR5AR1 | DCSTAMP |
| **FAT2** | **TMEM51** | **OAS3** | TAF1B | NEUROG1 | LCE1E |
| **CRY2** | **RUNX1T1** | **OR8G2** | AMPD2 | CCDC130 | HRH2 |
| **PSMC1** | **TAMM41** | **KRTAP19-7** | PSMA1 | AMPH | ABHD10 |
| **RBM8A** | **APC** | **HMGN3** | ABCA5 | KRTAP22-2 | RP1-257A7.4 |
| **IFNA13** | **KRTAP29-1** | **RAB39B** | LRRC3 | OR56B1 | AURKA |
| **ZC3H12A** | **RNF139** | **TBC1D1** | ZNF91 | KIAA1279 | PGLYRP3 |
| **MCFD2** | **IDS** | **CTSL** | PSMF1 | OR4N5 | FAM153B |
| **SLC38A11** | **FAM69C** | **GABPB1** | C1orf127 | RNF103-CHMP3 | MAGEC1 |
| **CSRNP2** | **CBWD3** | **KRTAP6-2** | GPR22 | VSIG2 | C11orf53 |
| **LCP2** | **KIAA1456** | **GRIP1** | MFF | ME1 | CA1 |
| **OR52A1** | **KLHL31** | **NNT** | MAOB | PPEF1 | KLK2 |
| **HDHD2** | **KLRC1** | **OR13C4** | OR5H2 | PALMD | RLN1 |
| **RFXAP** | **SMC5** | **DEK** | FCRLB | TTLL6 | KRT28 |
| **TNMD** | **CNGA2** | **CTBS** | CGRRF1 | OR10K1 | DEPTOR |
| **BAALC** | **CCDC129** | **OR10H4** | F2RL2 | PCYT1B | NBN |
| **MAGEC3** | **RCOR3** | **ASCC1** | OR1L4 | PAQR9 | PLA2G5 |
| **CDKN2C** | **STYK1** | **ECSCR** | GPR15 | CDK2AP1 | WDR92 |
| **PELI1** | **G6PC** | **HIATL1** | TSPAN12 | GART | NUDT12 |
| **IL20RA** | **SLA** | **KLF7** | MSH3 | SPTA1 | KRT39 |
| **ARL1** | **INHBE** | **OR6N1** | SPG7 | ZFP90 | PTPN20B |
| **SCP2** | **NETO2** | **CDCA3** | GSAP | MYL1 | PEX3 |
| **SERPINB2** | **TAS2R9** | **KIF2A** | OR12D3 | ZNF713 | IL17A |
| **CBR4** | **CCR3** | **OR5AN1** | MPPED2 | CLDN15 | CLDN24 |
| **KRT23** | **ZNF433** | **GPR133** | ST6GALNAC6 | ARMCX1 | PTPN20A |
| **TPM3** | **TMEFF1** | **ZSCAN4** | SMC4 | RRAS2 | MTRNR2L4 |
| **PPP3R1** | **KIR2DL1** | **CCT2** | TMEM87B | LYG2 | TMEM62 |
| **ERMP1** | **PPP2CA** | **MED10** | SLC39A8 | USF1 | LOC388813 |
| **EIF2AK4** | **MICAL2** | **FUT5** | TDRP | NAP1L1 | ZNF407 |
| **RPP40** | **KIF18B** | **CACNG1** | XCL1 | SRP72 | TAX1BP1 |
| **ZNF646** | **CASP8** | **GFI1** | OR2AG2 | DYDC2 | HHLA1 |
| **FBXL3** | **MT4** | **ATP5C1** | CSN2 | OR2M2 | LY86 |
| **KIAA0196** | **RFPL4A** | **PCDHA8** | DNAJC8 | MBNL1 | SSR2 |
| **JAK1** | **SMAD5** | **ADAM28** | RBFOX3 | HIST1H4I | ERCC6-PGBD3 |
| **OR5I1** | **C9orf153** | **RPL4** | OR10A2 | CECR2 | ASAH2 |
| **DLC1** | **NDUFAF3** | **RCAN3** | IPO8 | FOXC2 | CCNI2 |
| **TIAM2** | **EXOSC4** | **MGST1** | ZCCHC16 | ZNF672 | CDK4 |
| **NANS** | **EID1** | **CLEC4G** | SOX17 | MORF4L1 | OR5M3 |
| **ACSL6** | **FCRL3** | **SOX6** | FGF14 | TRNP1 | SLC16A9 |
| **CLEC3B** | **OR7C2** | **FOXD1** | CLDN11 | MAS1L | CES1 |
| **PTK2** | **MRPL32** | **TLR4** | MDC1 | ADCY10 | ATXN2 |
| **VN1R4** | **OR4L1** | **AADACL2** | HSD3B2 | FNTB | CEP70 |
| **BAAT** | **RGPD1** | **TBL2** | TTC23 | CCDC73 | GOSR2 |
| **AMFR** | **ECM2** | **F3** | RBFOX2 | CXXC4 | DOPEY1 |
| **NMT1** | **ORC3** | **OTOGL** | UPF3A | GSTO1 | NPFFR1 |
| **MT1B** | **OR10T2** | **CFP** | ZBTB8B | TFIP11 | NLRC5 |
| **SLC27A2** | **OR1S2** | **MIOS** | SKIV2L | RCC1 | OR11G2 |
| **C5orf51** | **OR13C2** | **KIF16B** | AK2 | TRIM10 | DYNLL1 |
| **CYP4F2** | **LRRC39** | **LONRF2** | IL2 | SPAM1 | PLS1 |
| **SELK** | **ZNF471** | **PCDHB4** | C8orf44 | SPINK6 | RNASE4 |
| **DYX1C1** | **HYLS1** | **MUC15** | SLC38A4 | ELF5 | BCL2L14 |
| **NUP107** | **VPS37A** | **UHMK1** | ARHGAP15 | HADH | GNB3 |
| **CPO** | **ZNF280D** | **UBA6** | GLUD2 | RCCD1 | POLA1 |
| **OR52N5** | **KLF4** | **ABAT** | VCPIP1 | CYP7A1 | FBXL8 |
| **OIT3** | **ZIC4** | **PCDHB15** | SLC13A4 | NR2F2 | VN1R1 |
| **RNASE8** | **DDX25** | **DKK4** | STS | NDST3 | HARS |
| **IGIP** | **NARG2** | **ABCC2** | RBM43 | TPTE2 | GBP4 |
| **JAK3** | **YIPF7** | **STAMBPL1** | HARBI1 | KCNMB2 | SNX30 |
| **REM2** | **RUNX1** | **SCHIP1** | DTNA | PON1 | FOXP1 |
| **TRIM43B** | **SERPINE1** | **EMCN** | ACOX3 | RPN2 | MEF2B |
| **DFFA** | **OR4D11** | **FNTA** | FABP6 | OR1D5 | JAM3 |
| **RCAN1** | **ANKUB1** | **SPECC1L** | OR4C3 | C6orf10 | IGSF9 |
| **DMD** | **HIST1H1D** | **RHOD** | C8orf34 | ARHGEF33 | AMBP |
| **C12orf60** | **PCDHA5** | **KCNK6** | HN1L | OVCH2 | SPDYE2 |
| **PCDHA3** | **ERG** | **TMPRSS11B** | SERINC1 | TTC1 | STX19 |
| **CNOT2** | **MRPS36** | **ICA1** | MMP28 | CDX4 | SPATC1L |
| **TMEM47** | **MAGEB16** | **NRAS** | DNAH8 | CCT6B | APOBEC1 |
| **KPNA2** | **ZNF14** | **PCDHGA12** | C10orf67 | RHBDL2 | WDR74 |
| **FAM69A** | **RABGEF1** | **FAM103A1** | ANKS4B | PPIL1 | WAPAL |
| **PTGDR2** | **RALGAPA1** | **TVP23B** | EPB41L2 | ELF2 | C16orf46 |
| **MBD3L2** | **BTN3A2** | **FAM71D** | TULP2 | RPP38 | DEFB127 |
| **TCEAL2** | **CAPS** | **MLPH** | RFX3 | KIAA1147 | PLEKHA5 |
| **INSC** | **CADM2** | **IFNL3** | AMPD3 | SLC16A2 | TCEAL7 |
| **SSTR2** | **MRPS27** | **CDK14** | NCALD | TMEM207 | PPP1R14B |
| **POFUT2** | **CXorf27** | **CST11** | TTC21B | GPC2 | HAUS5 |
| **TNFSF15** | **RP11-402G3.3** | **MARCO** | TRAPPC2 | SCNM1 | COTL1 |
| **SDCBP2** | **TYW1** | **HHEX** | OR6V1 | MEPE | ERAS |
| **XKR3** | **BTN3A3** | **SPINK9** | OR4M2 | LYZL6 | RASGRP2 |
| **CHIA** | **TCP11X2** | **C22orf42** | KRT6B | DAAM2 | GOLT1B |
| **HN1** | **TEX15** | **AHCYL2** | GSTK1 | SLC9A6 | CAB39 |
| **FOXP2** | **NDUFS2** | **NCEH1** | CCND3 | FBXW7 | FOXP3 |
| **SLC2A3** | **HIST1H2AG** | **SLC6A13** | KRT2 | ABCG2 | OLFML2A |
| **APBB1** | **PPP1R3A** | **C2CD3** | RECK | HYI | PCDHGB2 |
| **CNNM1** | **OR5K3** | **SSH2** | SERPINB12 | TSHZ2 | ERLIN1 |
| **TBC1D2B** | **ADAM32** | **PDPN** | GJD4 | F9 | WNT16 |
| **ZFP14** | **LPPR5** | **CNTN1** | TBL1Y | IL21R | EIF3M |
| **TIPARP** | **AC004019.13** | **THSD7B** | MRPS18A | F13B | NR2F1 |
| **CEP85** | **SP110** | **ROCK1** | CREB3L4 | NIPA1 | MAGED2 |
| **MID1** | **PLCH1** | **DNAJC5B** | CHPF2 | PHOSPHO2 | IER5L |
| **GTPBP4** | **C14orf105** | **UVRAG** | OLR1 | SULT1A1 | OXNAD1 |
| **LGALS7** | **RBBP9** | **PPIAL4G** | C15orf41 | RANBP9 | SURF4 |
| **GTPBP10** | **H2BFM** | **NKRF** | CDH18 | ZBTB5 | ANKRD1 |
| **SLC22A15** | **METTL15** | **NPIPA3** | CADM1 | GJD3 | DCAF5 |
| **PPP2R2B** | **ACOX1** | **JOSD1** | PMVK | LRRC3B | TPRN |
| **AGBL1** | **RGS4** | **PPIAL4C** | CDH9 | ABCC10 | PUM1 |
| **NLRP14** | **C3AR1** | **EMILIN2** | MOSPD2 | GTSF1 | PLEKHD1 |
| **FAM46C** | **CXorf57** | **NPIPA1** | CD86 | KLHL23 | LIX1 |
| **CAPRIN2** | **C12orf50** | **GOPC** | CD34 | RPS27 | ZC4H2 |
| **DEPDC5** | **IFIT2** | **KIAA0430** | ZNF836 | CLCN3 | PLCE1 |
| **DTYMK** | **CDRT4** | **ABCG8** | USP25 | ETFB | EIF2B2 |
| **PDE2A** | **CBWD2** | **RPS4X** | YY1AP1 | NFASC | LRRC4C |
| **NFIB** | **IDI2** | **NLRP10** | OR8I2 | KLRC4-KLRK1 | PARN |
| **MYOF** | **WASL** | **C11orf70** | DOCK9 | ITGB3BP | ASTN1 |
| **ADAM21** | **MCTP1** | **TRIM4** | RAD1 | PYGO2 | EGFL8 |
| **AADACL4** | **SMR3A** | **RPS6KA5** | PDE1C | KLRC3 | KYNU |
| **LRCH2** | **OPRD1** | **PPIAL4B** | WDR25 | FXYD6 | OR52I2 |
| **VWA2** | **LMO2** | **C12orf54** | H2AFX | KRT31 | SPRY1 |
| **CDC42EP3** | **CDK6** | **FMNL2** | IQGAP3 | METTL7B | RPL21 |
| **HNMT** | **AMOT** | **KLHL8** | UGT3A1 | ENPP6 | SOS1 |
| **CCDC69** | **PITX2** | **ZNF3** | KCMF1 | LRRC18 | VDR |
| **C8orf46** | **MYO16** | **SPINT3** | SENP6 | SERF2 | FAXDC2 |
| **PARP4** | **LTA** | **FAM122C** | GDI1 | ANP32B | PRPS2 |
| **ZNF225** | **RAG2** | **DLGAP1** | CCKAR | NOX3 | AC022173.2 |
| **NBPF9** | **ALPK1** | **EYA1** | ZNF365 | SLC37A4 | RPRD1A |
| **C19orf10** | **GGH** | **TRDMT1** | C1orf227 | DUOX1 | ZCCHC13 |
| **LPP** | **RABGAP1L** | **TOMM70A** | ERP29 | LMBRD1 | PRSS23 |
| **NBPF16** | **AWAT2** | **TAS2R3** | AGTPBP1 | AGXT2 | PPP5C |
| **WNT1** | **PLS3** | **RGS21** | CYLC2 | TMEM205 | OR1E2 |
| **TMEM217** | **EMX2** | **ALOX5AP** | TGFB2 | C6orf99 | NDUFB6 |
| **FAM127C** | **SGCG** | **IBSP** | SLC8B1 | VN1R2 | SPAG1 |
| **UBAP2** | **PAQR3** | **SETBP1** | ISCA1 | PNCK | B3GALT2 |
| **PABPC1** | **EVI2B** | **LSM6** | OR8G5 | FCRL5 | PCDH11X |
| **EMC1** | **NGB** | **STARD13** | C3orf84 | TMEM5 | SPP1 |
| **CFHR1** | **LPIN3** | **UBE2V1** | CASP14 | GRIN3A | CD47 |
| **SNCA** | **C7orf49** | **HRNR** | SAMD13 | CRYZ | CCDC88A |
| **PFDN4** | **DHX32** | **DEFB4A** | KRTAP21-3 | NOP58 | ECI2 |
| **SPANXA2** | **NPTXR** | **MTMR2** | CD1E | RAB3GAP2 | LCE2B |
| **PIH1D1** | **ZNF648** | **FAM13A** | EIF4EBP2 | WDR27 | CPEB4 |
| **STMN2** | **C4orf22** | **RNF141** | PLGLB1 | SDS | ELF1 |
| **COA7** | **R3HCC1L** | **TBC1D3** | GMDS | OR10H5 | FAM71C |
| **RAPGEF4** | **CYP3A5** | **PHLDB2** | PATE2 | WDR19 | DKKL1 |
| **TMCO5A** | **C4orf33** | **EFEMP1** | CHCHD1 | URGCP | SIT1 |
| **RHAG** | **NHP2L1** | **TAAR5** | SCARB1 | SIL1 | CSTF1 |
| **TTC30A** | **HMCN1** | **GRID2** | MITF | WDR82 | THOC3 |
| **CCDC127** | **GSX1** | **EIF4G2** | MUC17 | PIGB | ZC2HC1A |
| **RTTN** | **PABPC4L** | **TBC1D3F** | OR4D6 | DNAJC28 | PCDH11Y |
| **CEP164** | **MTUS2** | **CYP4A22** | GABRA2 | MAP3K1 | ANKS1B |
| **C4BPB** | **TUSC1** | **DHRS9** | ZFP37 | NAT1 | ARHGAP20 |
| **MPHOSPH10** | **MMP20** | **TKTL2** | HPR | ATP5O | CNPY3 |
| **SEC22C** | **ASB2** | **CCDC7** | HIST1H2BG | WWTR1 | NPIPB9 |
| **OR5F1** | **LIMA1** | **UBE2E1** | BIN3 | ACTB | OR2A42 |
| **CLDN10** | **FAP** | **S100A12** | OR56A3 | ARID4B | CHRFAM7A |
| **DIRC1** | **PTTG1** | **FABP4** | C1orf194 | PABPN1 | ADRM1 |
| **CCT7** | **TRDN** | **KRT26** | PCDHA7 | PCDHA2 | NCKAP1L |
| **C1orf61** | **ZDHHC15** | **SLC4A7** | ZFC3H1 | C11orf45 | GRAMD1C |
| **OR8K1** | **TRIM49C** | **SPANXN2** | RNF25 | VPS53 | PNO1 |
| **FYB** | **DYNC2H1** | **PERP** | C9orf91 | ZNF609 | RBMS3 |
| **ZNF443** | **ATP13A4** | **TESPA1** | BCAR1 | DUPD1 | VGLL4 |
| **C2orf68** | **TMPRSS12** | **SLC26A7** | SERP1 | NUPR1L | PLEKHA6 |
| **UNC93A** | **CT55** | **OR13A1** | PTPRO | TFB2M | OR6C1 |
| **HEXIM2** | **PRR23D2** | **CKAP2** | RBM15 | FOXG1 | TOMM7 |
| **MED19** | **EPHB6** | **KRTAP4-11** | PIK3C2A | AKNAD1 | CCK |
| **NLRP11** | **MYO19** | **EPM2AIP1** | BCMO1 | ERVMER34-1 | CNDP2 |
| **MON1A** | **LSG1** | **OR2AP1** | OR2G6 | PDE6H | STOX2 |
| **GPT** | **EBNA1BP2** | **TARS** | GNG2 | CCDC18 | KATNA1 |
| **ZNF787** | **OR4F15** | **MCEE** | ZNF426 | SCG2 | AQP1 |
| **ZNF250** | **DHDH** | **TMEM252** | RAB40A | MIS18BP1 | DDOST |
| **SHISA3** | **ZNF217** | **SETMAR** | TRIP12 | BCAR3 | EYS |
| **OR5B21** | **HBE1** | **MSRA** | ZBTB32 | SPINK2 | LMNA |
| **AMY1A** | **RAB27B** | **ZNF83** | SLC5A12 | LAMTOR5 | TRPM6 |
| **C2orf80** | **FGFBP2** | **KLF9** | SNX15 | OR7D2 | MEOX1 |
| **PLAU** | **LUC7L** | PSMC3IP | TMPRSS11D | ARMC7 | ECSIT |
| **DMTN** | **XKRY2** | KCNQ3 | LIPK | UNC5D | GPATCH8 |
| **GLA** | **CNTN4** | WDR78 | ACSF3 | DAB2IP | MYL3 |
| **MS4A5** | **OR51M1** | R3HDM2 | OR2B3 | ZNF391 | LRRC40 |
| **FBXL16** | **OR2A2** | COL5A2 | PCDHGA2 | SIN3A | CDCA4 |
| **CDKL1** | **HBS1L** | NCOA4 | CPN1 | OVOL3 | OR10G7 |
| **PCDHA13** | **NEIL2** | WLS | DPF1 | LIPJ | DUT |
| **SENP8** | **SIDT1** | C16orf78 | IFT80 | CCDC146 | SMTNL1 |
| **MMRN2** | **PNKP** | AFF4 | AMPD1 | RHD | FAIM |
| **C3orf79** | **MYO3B** | VPS13A | PCDHGA11 | CHRNB4 | TCP10 |
| **OR2T27** | **ZNF25** | TECTA | UBE2N | TMPRSS11A | TRPC7 |
| **DCP1B** | **FAM173B** | CYP11B1 | UGT1A6 | TTC34 | PARK7 |
| **FGF11** | **GPR116** | AOX1 | ITIH6 | RIN2 | UBE3D |
| **TES** | **TMEM257** | MAPK15 | CROT | PRCD | KIRREL |
| **PGBD1** | **OR4P4** | KRTAP19-3 | SMCO2 | GPR3 | LEMD3 |
| **PPM1J** | **MACC1** | FCRL4 | KIF20B | SERPINB6 | HERPUD1 |
| **IL18RAP** | **SIGLEC14** | C12orf66 | SLC35F5 | TAS2R16 | PSMA2 |
| **DGCR6L** | **STARD4** | ARL17A | TTC28 | SIRT2 | HOXB9 |
| **ZNF571** | **FAM110A** | BMX | KIFAP3 | REP15 | COMMD5 |
| **UGT1A9** | **OR5D13** | ZBTB38 | FOXR2 | RB1CC1 | CIAPIN1 |
| **CCNH** | **ZNF488** | FAM120B | TRPC5 | ALPK3 | CAPN8 |
| **FAM156A** | **CMPK2** | XRN1 | MOS | SGMS2 | ANG |
| **RHOJ** | **CBWD5** | MSR1 | KCP | METTL11B | TCP10L |
| **CRYBB3** | **ELOVL5** | SSX2IP | C1orf105 | ERAP1 | RAD23B |
| **SPP2** | **RAD54B** | MTUS1 | ARHGEF9 | MAP3K10 | CES3 |
| **MN1** | **ZNF597** | USP5 | PLEKHS1 | PSAPL1 | SMYD1 |
| **SNRPF** | **INSL5** | MAGEB10 | PSIP1 | NOS2 | FBP2 |
| **PAGE2** | **RAB23** | ZNF772 | XKR7 | NOTUM | CCBL2 |
| **BBIP1** | **RBMS2** | PCDHA1 | TNP2 | PENK | HLTF |
| **LOC154872** | **CALCRL** | CDON | GPR52 | NRAP | HIST1H1E |
| **GGTLC1** | **FATE1** | HFM1 | REV3L | TNFSF4 | CDC14B |
| **METTL20** | **RFX4** | C10orf55 | SLC7A3 | FREM1 | ZNF417 |
| **CYP27C1** | **CTXN3** | COMMD2 | PPFIBP2 | SARM1 | IGSF9B |
| **PLEKHB1** | **DDX6** | SLC11A1 | LYPD5 | ARHGEF4 | EBLN2 |
| **DEFB121** | **ZNF852** | ITIH2 | PRDM2 | RPF2 | PDCD7 |
| **TIGD6** | **C19orf80** | PFN2 | FAM204A | CCDC158 | HLCS |
| **IL13RA2** | **HMOX2** | BAZ1A | ANTXR2 | BPIFB1 | ZNF618 |
| **RNF222** | **SLC4A5** | MGP | HNF4A | MEST | TSGA10 |
| **EIF3L** | **ZNF415** | ZWILCH | MTIF3 | NT5DC1 | ROBO1 |
| **SLC24A2** | **GATM** | ZNF43 | PPARD | CDH2 | SPDYE2B |
| **KLHL24** | **PNMA3** | LNX1 | MYL12A | INPP5F | OR2L8 |
| **DLX6** | **KCNIP4** | CCDC71L | PDC | NMUR2 | PCDHA9 |
| **IFNW1** | **COL15A1** | CUL3 | SYTL2 | ALG8 | ISG15 |
| **SERINC3** | **TCHP** | SYTL5 | COX8C | TBCCD1 | SLC9A7 |
| **UTF1** | **MON2** | P2RY13 | HPRT1 | ZNF830 | PEPD |
| **MAN1A1** | **NME9** | RERGL | OR4A47 | PI3 | PPP2R2C |
| **QPCTL** | **FCAR** | BEX2 | SLC8A2 | C6orf89 | PCDHB3 |
| **ST6GAL1** | **DALRD3** | THUMPD3 | KIAA0020 | FAM107B | SVIP |
| **CARD8** | **PHIP** | NOX5 | SLFN14 | TCF20 | DTX2 |
| **DNTTIP1** | **MPP4** | NRCAM | NEBL | DENND2A | ATG14 |
| **OR4C13** | **FUNDC2** | SYT1 | ERLEC1 | TRIT1 | MOV10 |
| **TSPYL6** | **TLR10** | ENC1 | CWC15 | CHAC2 | RRM2 |
| **FAM217A** | **OR13F1** | SLC22A9 | LCE3C | SCMH1 | LIMS1 |
| **ACBD5** | **HHIPL2** | BRINP1 | PIP | SERPINA1 | KIF18A |
| **SENP5** | **HSPB8** | PRKAR1B | KRT6C | VPS72 | DUSP27 |
| **XIRP2** | **NLRP8** | SLCO1B3 | WDR52 | NXPE3 | ACOT12 |
| **SPANXA1** | **INO80D** | OR5K4 | KCTD4 | MRE11A | TAF10 |
| **CFHR5** | **PELO** | SH2D1B | SERPINB7 | ARHGAP17 | FAM154B |
| **SYBU** | **OR9Q2** | CENPC | CNTN6 | DNAJB5 | NCOR1 |
| **GOLGA6L1** | **OR13C5** | SLCO1A2 | GNPTAB | MPP7 | B3GALNT1 |
| **CLUL1** | **KDM2B** | OR5K1 | RNF182 | SPRR2A | DNAH17 |
| **ALDH16A1** | **ZNF583** | PPFIA2 | CHST11 | SULT1A2 | IQCA1 |
| **SMARCAD1** | **SPATA24** | AP3B1 | ANGPTL3 | SMIM2 | FUBP3 |
| **F12** | **ZNF484** | OR1K1 | PLCB1 | SP7 | SI |
| **SPANXN4** | **TMEM128** | FGL2 | IL3RA | CCDC173 | CD93 |
| **ZC3H13** | **TMED4** | PPM1A | RANBP6 | COLCA2 | CSTF3 |
| **SLCO4C1** | **KAAG1** | C22orf43 | MOBP | PAGR1 | NPNT |
| **SLC30A8** | **ZNF835** | CPED1 | OR5W2 | BTG2 | SKIL |
| **RUFY1** | **PUS3** | GSPT2 | ZNF350 | ARL11 | DEFB132 |
| **HTR3A** | **SLTM** | LIPA | PDE4B | KLK14 | CEACAM21 |
| **TBC1D10B** | **OR52E4** | HIF1A | ZNF148 | DERL1 | TMEM212 |
| **CD83** | **C9orf84** | CSMD1 | SORBS2 | PARS2 | TINAGL1 |
| **SPCS3** | **OR4D1** | FEZF1 | TMEM8A | GMEB2 | TUBB |
| **TIMMDC1** | **IPO4** | MBLAC2 | STAT4 | KLRC4 | IL17B |
| **ZC2HC1B** | **H6PD** | WDR34 | NT5C3A | EFCAB7 | RAG1 |
| **FPR2** | **PIK3R1** | IFIT3 | BEGAIN | GTSCR1 | SDAD1 |
| **PPP1R14D** | **ATP1A2** | FOXA2 | TMSB10 | LEMD1 | PRAMEF13 |
| **POU4F1** | **MARVELD2** | TSHB | RFK | FXYD6-FXYD2 | ABCD2 |
| **RAI14** | **SBDS** | MRPS12 | C15orf48 | KRT33A | PLEKHB2 |
| **ADCYAP1** | **NKX2-1** | STATH | CLDN8 | EFNA1 | RHOG |
| **PGBD3** | **GSTM2** | ITGBL1 | NSL1 | HOXA6 | CSF2RB |
| **DNASE1** | **SCGB1D4** | DYRK1B | BMP6 | KLRC2 | PGRMC1 |
| **RAF1** | **ZNF675** | PTPN4 | TRIM42 | KRT35 | RASAL2 |
| **PRADC1** | **VAT1L** | PCYT2 | MRPL35 | PPIP5K1 | MYBL2 |
| **DUOXA1** | **KLHDC1** | SLC10A2 | CD1B | CORO1C |  |
| **CYYR1** | **BTN1A1** | PPBP | OR52N4 | CLDN17 |  |
| **OR5J2** | **AGFG1** | CALCR | SNX18 | CDC42BPB |  |
| **B3GAT2** | **FLRT3** | ARHGEF15 | GOT2 | SFRP4 |  |
| **B4GALNT1** | **FBXO25** | C4orf32 | GLTPD2 | NEGR1 |  |

**Table S11. Other categories in the IPA pathway analysis of TLX3 target genes**

The table lists the additional categories of diseases and cellular functions in the IPA software analysis of the predicted TLX3 regulated genes. The second column contains the range of p-values that indicate non-random associations between molecules (3^rd^ column) and categories.

| **Diseases and disorders** | **p-values** | **Number of molecules** |
| --- | --- | --- |
| Cancer | 1.07 x10-7 - 4.65 x10-2 | 595 |
| Hematological Disease | 4.06 x10-4 - 4.81 x10-2 | 42 |
| Organismal Injury and Abnormalities | 4.06 x10-4 - 3.40 x10-2 | 29 |
| Gastrointestinal Disease | 4.45 x10-4 - 4.39 x10-2 | 294 |
| Immunological Disease | 1.23 x10-3 - 4.98 x10-2 | 75 |
| **Molecular and cellular functions** | **p-values** | **Number of molecules** |
| Energy Production | 9.31 x10-5 - 2.35 x10-2 | 9 |
| Lipid Metabolism | 9.31 x10-5 - 4.61 x10-2 | 34 |
| Small Molecule Biochemistry8 | 9.31 x10-5 - 4.61 x10-2 | 41 |
| Drug Metabolism | 1.33 x10-4 - 3.40 x10-2 | 15 |
| Carbohydrate Metabolism | 5.10 x10-4 - 2.34 x10-2 | 10 |

**Table S12: Enriched subcategories within the “Physiological system development and function”** **category**

The list shows details of the subcategories within the “Physiological system development and function” category, including enriched networks and molecules, from the IPA pathway analysis using TLX3 predicted genetic targets. 4^th^ column displays association p-values between molecules and categories.

| **Hematological System Development And Function** | **Molecules** | **# molecules** | **p-value** |
| --- | --- | --- | --- |
| differentiation of monocyte-derived dendritic cells | TLR4,TNF,TNFSF11 | 3 | 2.36 x10^-3^ |
| quantity of dendritic cells | CD83,CTLA4,TNF | 3 | 2.36 x10^-3^ |
| chemotaxis of mononuclear leukocytes | CCL11,CCL20,CCR3,DEFB4A/DEFB4B,DEK,FPR1,FPR2,OPRD1,PTGDR2,ROCK1,S100A12,SELE,SERPINE1,TNF,TNFSF11 | 15 | 2.60 x10^-3^ |
| arrest in differentiation of granulocytes | RUNX1,RUNX1T1 | 2 | 2.60 x10^-3^ |
| expansion of erythroid cells | RUNX1,RUNX1T1 | 2 | 2.60 x10^-3^ |
| chemotaxis of monocytes | CCL11,FPR1,FPR2,OPRD1,PTGDR2,S100A12,SELE,SERPINE1,TNF,TNFSF11 | 10 | 3.11 x10^-3^ |
| chemotaxis of phagocytes | CCL11,CCL20,CCR3,CCR6,DEFB4A/DEFB4B,DEK,FPR1,FPR2,OPRD1,PLAU,PTGDR2,S100A12,SELE,SERPINE1,TLR4,TNF,TNFSF11 | 17 | 3.37 x10^-3^ |
| cell movement of monocytes | CCL11,CCL20,FPR1,FPR2,IFNB1,JAK1,OPRD1,PTGDR2,S100A12,SELE,SERPINE1,TNF,TNFSF11 | 13 | 3.38 x10^-3^ |
| respiratory burst of neutrophils | ADCYAP1,FCAR,FPR1,TNF | 4 | 4.47 x10^-3^ |
| differentiation of dendritic cells | DEK,EIF2AK4,IFNA1/IFNA13,IFNB1,IFNW1,TLR4,TNF,TNFSF11 | 8 | 5.13 x10^-3^ |
| osteoclastogenesis of PBMCs | ADM,TNF,TNFSF11 | 3 | 6.12 x10^-3^ |
| quantity of leukocytes | CD83,CTLA4,DOCK8,FPR1,JAK3,LCK,MOG,PIK3R1,PRL,TNF | 10 | 6.16 x10^-3^ |
| colony formation of granulocyte-macrophage progenitor cells | RPS6KA5,TNF | 2 | 7.54 x10^-3^ |
| cell movement of myeloid cells | CCL11,CCL20,CCR3,CCR6,DEFB4A/DEFB4B,DEK,FCAR,FPR1,FPR2,IFNB1,JAK1,OPRD1,PLAU,PTGDR2,S100A12,SELE,SERPINE1,TLR4,TNF,TNFSF11 | 20 | 8.61 x10^-3^ |
| cell movement of phagocytes | CCL11,CCL20,CCR3,CCR6,DEFB4A/DEFB4B,DEK,FCAR,FPR1,FPR2,IFNB1,JAK1,OPRD1,PLAU,PTGDR2,S100A12,SELE,SERPINE1,TLR4,TNF,TNFSF11 | 20 | 1.09 x10^-2^ |
| colony formation of erythroid progenitor cells | RPS6KA5,RUNX1,RUNX1T1,TNF | 4 | 1.16 x10^-2^ |
| differentiation of peripheral blood leukocytes | IFNA1/IFNA13,IFNW1,TNF,TNFSF11 | 4 | 1.16 x10^-2^ |
| cell movement of mononuclear leukocytes | CCL11,CCL20,CCR3,DEFB4A/DEFB4B,DEK,FPR1,FPR2,IFNB1,JAK1,OPRD1,PLAU,PTGDR2,ROCK1,S100A12,SCGB1D4,SELE,SERPINE1,TLR4,TNF,TNFSF11 | 20 | 1.30 x10^-2^ |
| migration of eosinophils | CCL11,CCR3,PTGDR2,TNF | 4 | 1.41 x10^-2^ |
| chemoattraction of T lymphocytes | DEFB4A/DEFB4B,DEK | 2 | 1.46 x10^-2^ |
| transdifferentiation of monocyte-derived dendritic cells | TNF,TNFSF11 | 2 | 1.46 x10^-2^ |
| chemotaxis of leukocytes | CCL11,CCL20,CCR3,CCR6,DEFB4A/DEFB4B,DEK,FPR1,FPR2,OPRD1,PLAU,PTGDR2,ROCK1,S100A12,SBDS,SELE,SERPINE1,TLR4,TNF,TNFSF11 | 19 | 1.55 x10^-2^ |
| cell movement of basophils | CCL11,FPR1,PTGDR2 | 3 | 1.61 x10^-2^ |
| differentiation of peripheral blood monocytes | IFNA1/IFNA13,IFNW1,TNFSF11 | 3 | 1.61 x10^-2^ |
| quantity of lymphocytes | DOCK8,JAK3,LCK,MOG,PIK3R1,PRL,TNF | 7 | 1.93 x10^-2^ |
| activation of monocyte-derived dendritic cells | HSPB8,TLR4,TNF | 3 | 2.58 x10^-2^ |
| differentiation of leukocytes | B4GALNT1,CASP8,CDK6,CTLA4,DEK,EIF2AK4,HDAC9,IFNA1/IFNA13,IFNB1,IFNL3,IFNW1,LCK,RUNX1,RUNX1T1,TLR4,TNF,TNFSF11 | 17 | 2.86 x10^-2^ |
| chemotaxis of myeloid cells | CCL11,CCR6,DEFB4A/DEFB4B,DEK,FPR1,FPR2,OPRD1,PTGDR2,S100A12,SELE,SERPINE1,TLR4,TNF,TNFSF11 | 14 | 3.01 x10^-2^ |
| chemotaxis of dendritic cells | CCL20,CCR3,CCR6,DEFB4A/DEFB4B,TNF | 5 | 3.12 x10^-2^ |
| quantity of T lymphocytes | DOCK8,JAK3,LCK,PRL,TNF | 5 | 3.12 x10^-2^ |
| chemoattraction of leukocytes | CCL20,DEFB4A/DEFB4B,DEK | 3 | 3.16 x10^-2^ |
| differentiation of myeloid progenitor cells | RUNX1,RUNX1T1,TNF | 3 | 3.16 x10^-2^ |
| quantity of phagocytes | CD83,CTLA4,FPR1,TNF | 4 | 3.17 x10^-2^ |
| chemotaxis of basophils | FPR1,PTGDR2 | 2 | 3.40 x10^-2^ |
| stimulation of erythroid cell lines | IFNB1,TNF | 2 | 3.40 x10^-2^ |
| stimulation of granulocytes | CCL11,TNF | 2 | 3.40 x10^-2^ |
| activation of dendritic cells | HSPB8,IFNB1,LTA,TLR4,TNF | 5 | 3.48 x10^-2^ |
| chemotaxis of granulocytes | CCL11,CCR6,DEFB4A/DEFB4B,DEK,FPR1,PTGDR2,S100A12,SELE,TLR4,TNF | 10 | 3.53 x10^-2^ |
| differentiation of antigen presenting cells | CASP8,DEK,EIF2AK4,IFNA1/IFNA13,IFNB1,IFNW1,TLR4,TNF,TNFSF11 | 9 | 3.76 x10^-2^ |
| cell movement of memory T lymphocytes | CCL20,DEFB4A/DEFB4B,PTGDR2 | 3 | 3.81 x10^-2^ |
| chemotaxis of PBMCs | CCL20,FPR1,FPR2 | 3 | 3.81 x10^-2^ |
| chemotaxis of neutrophils | CCR6,DEFB4A/DEFB4B,DEK,FPR1,S100A12,SELE,TLR4,TNF | 8 | 3.95 x10^-2^ |
| migration of granulocytes | CCL11,CCR3,FPR1,PLAU,PTGDR2,TNF | 6 | 4.09 x10^-2^ |
| coagulation of blood | C4BPB,CYP2C9,CYP4F2,F12,F3,TLR4,WASL | 7 | 4.39 x10^-2^ |
| differentiation of monocytes | B4GALNT1,IFNA1/IFNA13,IFNB1,IFNW1,TLR4,TNF,TNFSF11 | 7 | 4.39 x10^-2^ |
| colony formation of erythroid cells | RUNX1,RUNX1T1 | 2 | 4.61 x10^-2^ |
| migration of Langerhans cells | CCL20,TNF | 2 | 4.61 x10^-2^ |
| proliferation of myeloid progenitor cells | CCL20,TNF | 2 | 4.61 x10^-2^ |
| response of peripheral blood leukocytes | IFNA1/IFNA13,TLR4 | 2 | 4.61 x10^-2^ |
| **Hematopoiesis** | **Molecules** | **number** | **p-value** |
| differentiation of monocyte-derived dendritic cells | TLR4,TNF,TNFSF11 | 3 | 2.36 x10^-3^ |
| arrest in differentiation of granulocytes | RUNX1,RUNX1T1 | 2 | 2.60 x10^-3^ |
| expansion of erythroid cells | RUNX1,RUNX1T1 | 2 | 2.60 x10^-3^ |
| differentiation of dendritic cells | DEK,EIF2AK4,IFNA1/IFNA13,IFNB1,IFNW1,TLR4,TNF,TNFSF11 | 8 | 5.13 x10^-3^ |
| osteoclastogenesis of PBMCs | ADM,TNF,TNFSF11 | 3 | 6.12 x10^-3^ |
| colony formation of granulocyte-macrophage progenitor cells | RPS6KA5,TNF | 2 | 7.54 x10^-3^ |
| colony formation of erythroid progenitor cells | RPS6KA5,RUNX1,RUNX1T1,TNF | 4 | 1.16 x10^-2^ |
| differentiation of peripheral blood leukocytes | IFNA1/IFNA13,IFNW1,TNF,TNFSF11 | 4 | 1.16 x10^-2^ |
| transdifferentiation of monocyte-derived dendritic cells | TNF,TNFSF11 | 2 | 1.46 x10^-2^ |
| differentiation of peripheral blood monocytes | IFNA1/IFNA13,IFNW1,TNFSF11 | 3 | 1.61 x10^-2^ |
| differentiation of leukocytes | B4GALNT1,CASP8,CDK6,CTLA4,DEK,EIF2AK4,HDAC9,IFNA1/IFNA13,IFNB1,IFNL3,IFNW1,LCK,RUNX1,RUNX1T1,TLR4,TNF,TNFSF11 | 17 | 2.86 x10^-2^ |
| differentiation of myeloid progenitor cells | RUNX1,RUNX1T1,TNF | 3 | 3.16 x10^-2^ |
| generation of bone marrow cells | TNF,TNFSF11 | 2 | 3.40 x10^-2^ |
| differentiation of antigen presenting cells | CASP8,DEK,EIF2AK4,IFNA1/IFNA13,IFNB1,IFNW1,TLR4,TNF,TNFSF11 | 9 | 3.76 x10^-2^ |
| differentiation of monocytes | B4GALNT1,IFNA1/IFNA13,IFNB1,IFNW1,TLR4,TNF,TNFSF11 | 7 | 4.39 x10^-2^ |
| colony formation of erythroid cells | RUNX1,RUNX1T1 | 2 | 4.61 x10^-2^ |
| proliferation of myeloid progenitor cells | CCL20,TNF | 2 | 4.61 x10^-2^ |
| **Tissue Morphology** | **Molecules** | **number** | **p-value** |
| quantity of dendritic cells | CD83,CTLA4,TNF | 3 | 2.36 x10^-3^ |
| quantity of adipose tissue | ATP1A2,NMB,NR3C1,PLIN1,RUNX1T1,SERPINE1,TNF,UCP1 | 8 | 2.38 x10^-3^ |
| quantity of leukocytes | CD83,CTLA4,DOCK8,FPR1,JAK3,LCK,MOG,PIK3R1,PRL,TNF | 10 | 6.16 x10^-3^ |
| quantity of lymphocytes | DOCK8,JAK3,LCK,MOG,PIK3R1,PRL,TNF | 7 | 1.93 x10^-2^ |
| quantity of T lymphocytes | DOCK8,JAK3,LCK,PRL,TNF | 5 | 3.12 x10^-2^ |
| quantity of phagocytes | CD83,CTLA4,FPR1,TNF | 4 | 3.17 x10^-2^ |
| **Endocrine System Development and Function** | **Molecules** | **number** | **p-value** |
| oxidation of beta-estradiol | CYP2C8,CYP2C9,CYP3A4,CYP3A5 | 4 | 4.00 x10^-4^ |
| oxidation of hormone | CYP2C8,CYP2C9,CYP3A4,CYP3A5,DHRS9 | 5 | 9.33 x10^-4^ |
| metabolism of estrogen | CYP2C9,CYP3A4,CYP3A5,SULT1E1 | 4 | 7.49 x10^-3^ |
| conversion of hydrocortisone | H6PD,HSD11B1 | 2 | 7.54 x10^-3^ |
| hydroxylation of testosterone | CYP3A4,CYP3A5 | 2 | 7.54 x10^-3^ |
| metabolism of testosterone | CYP3A4,CYP3A5 | 2 | 7.54 x10^-3^ |
| metabolism of progesterone | ADM,CYP3A4,DHRS9,SCP2,STC1 | 5 | 1.93 x10^-2^ |
| metabolism of 17-alpha-ethinylestradiol | CYP2C9,CYP3A4 | 2 | 2.35 x10^-2^ |
| metabolism of beta-estradiol | CYP3A4,CYP3A5 | 2 | 2.35 x10^-2^ |
| oxidation of testosterone | CYP3A4,CYP3A5 | 2 | 2.35 x10^-2^ |
| metabolism of hormone | ADM,CYP3A4,CYP3A5,DHRS9,PRL,SCP2,STC1,TNF | 8 | 2.99 x10^-2^ |
| **Connective Tissue Development and Function** | **Molecules** | **number** | **p-value** |
| quantity of adipose tissue | ATP1A2,NMB,NR3C1,PLIN1,RUNX1T1,SERPINE1,TNF,UCP1 | 8 | 2.38 x10^-3^ |
| transdifferentiation of osteoclasts | TNF,TNFSF11 | 2 | 1.46 x10^-2^ |

**Table S13: List of motif decoys used in Z-score calculations**

List of 106 motifs of cluster representatives that were used as decoys in Z-score calculation.

| **Database** | **TF name** | **Database** | **TF name** | **Database** | **TF name** | **Database** | **TF name** |
| --- | --- | --- | --- | --- | --- | --- | --- |
| JASPAR | Tcfcp2l1 | JASPAR | Ar | UniPROBE | Crx | UniPROBE | Gat4 |
| JASPAR | HMRA2 | JASPAR | HNF4A | UniPROBE | Gm4881 | UniPROBE | Pou6f1 |
| JASPAR | RSC30 | JASPAR | CEBPA | UniPROBE | Hoxd11 | UniPROBE | Homez |
| JASPAR | Foxd3 | JASPAR | YAP7 | UniPROBE | IRC900814 | UniPROBE | Srf |
| JASPAR | RORA | JASPAR | NR3C1 | UniPROBE | Tcfap2c | UniPROBE | Cphx |
| JASPAR | SPIB | JASPAR | CEP3 | UniPROBE | Fli1 | UniPROBE | Bhlhb2 |
| JASPAR | CUP2 | JASPAR | REB1 | UniPROBE | Yll054c | UniPROBE | Aro80 |
| JASPAR | CAD1 | JASPAR | SuH | UniPROBE | Six6 | UniPROBE | Hoxa13 |
| JASPAR | AFT2 | JASPAR | ROX1 | UniPROBE | Ndt80 | UniPROBE | Ehf |
| JASPAR | Mycn | JASPAR | STP2 | UniPROBE | Hoxc13 | UniPROBE | Ets1 |
| JASPAR | AFT1 | JASPAR | En1 | UniPROBE | Pdr1 | UniPROBE | Tbf1 |
| JASPAR | IRF1 | JASPAR | NRG1 | UniPROBE | Etv3 | UniPROBE | Xbp1 |
| JASPAR | Pax4 | JASPAR | ttk | UniPROBE | Tec1 | UniPROBE | Hoxd13 |
| JASPAR | TEAD1 | JASPAR | ELK4 | UniPROBE | Pou2f3 | UniPROBE | Myf6 |
| JASPAR | NR2F1 | UniPROBE | Ehf | UniPROBE | Hoxa11 | UniPROBE | Myb |
| JASPAR | HMG-I | UniPROBE | Tcf2 | UniPROBE | Gata3 | UniPROBE | Sfl1 |
| JASPAR | EWSR1-FLI1 | UniPROBE | Tcfap2a | UniPROBE | Tgif1 | UniPROBE | Mybl1 |
| JASPAR | MAC1 | UniPROBE | Otx1 | UniPROBE | Hdx | UniPROBE | HLH-2 |
| JASPAR | z | UniPROBE | HLH-2 | UniPROBE | Hlx1 | UniPROBE | Elk4 |
| JASPAR | T | UniPROBE | Nkx2-3 | UniPROBE | Phd1 | UniPROBE | Gabpa |
| JASPAR | RREB1 | UniPROBE | Etv1 | UniPROBE | Rfx3 | UniPROBE | Rfx4 |
| JASPAR | CREB1 | UniPROBE | Ume6 | UniPROBE | Hoxd12 | UniPROBE | HLH-11 |
| JASPAR | D | UniPROBE | Elf5 | UniPROBE | Leu3 | UniPROBE | Hoxc11 |
| JASPAR | YNR063W | UniPROBE | PF14 | UniPROBE | HLH-2 | UniPROBE | Elk1 |
| JASPAR | ASH1 | UniPROBE | Elf3 | UniPROBE | Nsy-7 | UniPROBE | Etv5 |
| JASPAR | HMG-1 | UniPROBE | Hoxb13 | UniPROBE | Hoxc12 |  |  |
| JASPAR | Klf4 | UniPROBE | Cgd2 | UniPROBE | Elk3 |  |  |
