# Supplementary material for: Prediction of DNA binding motifs from 3D models of transcription factors; identifying TLX3 regulated genes
Source: Nucleic Acids Res. 2014 Nov 26;42(22):13500–12. doi: 10.1093/nar/gku1228 (PMC4267649; doi:10.1093/nar/gku1228)
Supplement: SUPPLEMENTARY DATA [file supp_gku1228_nar-02093-z-2014-File008.docx]

# RosettaDNA command used:

rosettaDNA @predspec.flags -database rosetta_database -run:constant_seed -nodelay

# The binding energy is extracted from the line that contains the calculated in the generated PDB file:

REMARK Binding energy: -17.959

# RosettaDNA files used:

predspec.flags

predspec.script

See contents of these two files on the next page

|  | **contents** |
| --- | --- |
| **predspec.flags** | -in:ignore_unrecognized_res  -file:s MYPDB  -score:weights dna  -use_input_sc  -ex1  -ex2  -ex2:operate  -ex3  -ex3:operate  -ex4  -ex4:operate  -extrachi_cutoff 1  -adducts dna_major_groove_water  -mute all  -unmute protocols.dna.RestrictDesignToProteinDNAInterface protocols.dna.DnaInterfacePacker protocols.dna.Specificity apps protocols.loops core.io.pdb  -mute core.io.database  -jd2:dd_parser  -parser:protocol predspec.script  -overwrite  -out:prefix ros_ |
| **predspec.script** | <dock_design>  <TASKOPERATIONS>  <InitializeFromCommandline name=IFC/>  <IncludeCurrent name=IC/>  <RestrictDesignToProteinDNAInterface name=DnaInt base_only=1 z_cutoff=3.0/>  <OperateOnCertainResidues name=AUTOprot>  <AddBehaviorRLT behavior=AUTO/>  <ResidueHasProperty property=PROTEIN/>  </OperateOnCertainResidues>  <OperateOnCertainResidues name=ProtNoDes>  <RestrictToRepackingRLT/>  <ResidueHasProperty property=PROTEIN/>  </OperateOnCertainResidues>  <OperateOnCertainResidues name=DnaNoPack>  <PreventRepackingRLT/>  <ResidueHasProperty property=DNA/>  </OperateOnCertainResidues>  </TASKOPERATIONS>  <SCOREFXNS>  <DNA weights=dna/>  <DNAGB weights=dna_gb/>  </SCOREFXNS>  <FILTERS>  <FalseFilter name=falsefilter/>  </FILTERS>  <MOVERS>  <DnaInterfacePacker name=DnaPack scorefxn=DNA task_operations=IFC,IC,AUTOprot,ProtNoDes,DnaInt binding=1 probe_specificity=1 minimize=0/>  </MOVERS>  <PROTOCOLS>  <Add mover_name=DnaPack/>  </PROTOCOLS>  </dock_design> |
